# The early childhood inhibitory touchscreen task: A new measure of response inhibition in toddlerhood and across the lifespan

**DOI:** 10.1371/journal.pone.0260695

**Published:** 2021-12-02

**Authors:** Karla Holmboe, Charlotte Larkman, Carina de Klerk, Andrew Simpson, Martha Ann Bell, Leslie Patton, Charis Christodoulou, Henrik Dvergsdal

**Affiliations:** 1 School of Psychological Science, University of Bristol, Bristol, United Kingdom; 2 Department of Experimental Psychology, University of Oxford, Oxford, United Kingdom; 3 Department of Psychology, University of Essex, Colchester, United Kingdom; 4 Centre for Brain and Cognitive Development, Department of Psychological Sciences, Birkbeck, London, United Kingdom; 5 Department of Psychology, Virginia Tech, Blacksburg, VA, United States of America; 6 Nord University Business School, Department of Entrepreneurship, Innovation and Organisation, Bodø, Norway; University of Trento, ITALY

## Abstract

Research into the earliest development of inhibitory control is limited by a lack of suitable tasks. In particular, commonly used inhibitory control tasks frequently have too high language and working memory demands for children under 3 years of age. Furthermore, researchers currently tend to shift to a new set of inhibitory control tasks between infancy, toddlerhood, and early childhood, raising doubts about whether the same function is being measured. Tasks that are structurally equivalent across age could potentially help resolve this issue. In the current report, a new response inhibition task, the Early Childhood Inhibitory Touchscreen Task (ECITT), was developed. This task can be minimally modified to suit different ages, whilst remaining structurally equivalent. In the new task, participants have to overcome a tendency to respond to a frequently rewarded location on a touchscreen and instead make an alternative response. The ECITT was validated in three independent studies (with additional data, *N* = 166, reported in Supporting Information). In Study 1 (*N* = 81), cross-sectional data indicated that inhibitory performance on the task improved significantly between 24 and 30 months of age. In Study 2 (*N* = 38), longitudinal data indicated steady improvement in inhibitory control between 18, 21 and 24 months, with significant stability in individual performance differences between each consecutive age in terms of accuracy (but not in terms of reaction time). Finally, in Study 3 (*N* = 64), inhibitory performance on a faster-paced version of the same task showed a similar developmental course across the lifespan (4–84 years) to other response inhibition tasks and was significantly correlated with Stop-signal performance. The ECITT extends the assessment of response inhibition earlier than previous tasks–into early toddlerhood. Because the task is simple and structurally equivalent across age, future longitudinal studies should benefit from using the ECITT to investigate the development of inhibitory control in a consistent manner across the toddler years and beyond.

## Introduction

Executive functions encompass a set of higher-order abilities, including working memory, inhibitory control, cognitive flexibility, and planning [1–3]. These important functions facilitate adaptation to new and complex situations when highly practiced, habitual, or short-term reward-driven processes are insufficient for goal-attainment or success. Inhibitory control (IC) is the ability to stop a thought, behaviour or action when an alternative response, or no response, is needed for optimal outcome. It is a multi-faceted construct, ranging from: the ability to resist temptation in classic *delay of gratification* and self-restraint tasks, essentially involving ‘waiting’ [4–6]; to ignoring distraction (*interference control*); to resisting distraction in working memory (*cognitive inhibition / resistance to proactive interference*); to inhibiting a prepotent motor response (*response inhibition / motor inhibition*) [6–8]. It is at present unclear to what extent these inhibitory functions are overlapping or distinct, but the evidence supports at least some separability of inhibitory functions in children and adults, at both the behavioural [8–11] and neural level [6] (see also Verbruggen & Logan [12], Box 3). In fact, in recent years evidence from large studies with adult participants has indicated that even purportedly similar IC tasks correlate poorly [10, 13]. This could be due to IC tasks measuring distinct inhibitory functions rather than a general inhibition construct [10]. However, these low correlations could also be due to measurement issues, such as low reliabilities of tasks used to measure inhibitory control [14, 15]. This makes it important to be both clear about which inhibitory function (or functions) is targeted by specific tasks and to establish the reliability of individual IC tasks.

In the present report, our focus is on a type of inhibitory control often referred to as *response inhibition*, which is the ability to stop a highly practiced (i.e., prepotent) motor response; and on how this function can best be measured in very early childhood, while still being comparable to measures later in childhood and during adulthood. At the neural level, response inhibition engages a functional brain network comprised of areas including: the prefrontal cortex, pre-supplementary motor area, parietal cortex and basal ganglia [6] (for review, see [16, 17]). This network develops substantially, with increases in both efficiency and neural specialisation, across the early childhood years and into adolescence [17].

Response inhibition is often assessed using the Go/NoGo task or the Stop-signal task. In a classic Go/NoGo task, participants have to respond as fast as possible to a frequently presented target but withhold their response when a rarer ‘NoGo’ stimulus is presented. Performance on the Go/NoGo task is typically indexed by the frequency of commission errors, i.e., the number of NoGo trials where the participant fails to inhibit the prepotent motor response built up on the Go trials [18]. In the Stop-signal task, participants have to perform a simple forced-choice discrimination task (e.g., press left key if they see a circle and right key if they see a square), but if they see or hear a signal (e.g., a tone) [19] when they are about to respond, they have to withhold that response.

Rapid improvement on the Go/NoGo task is seen in early childhood, particularly between 3 and 6 years [20–22]. Later in childhood, findings have been mixed, with some studies finding improvements in middle childhood [23, 24], but most finding a plateau in the developmental trend with limited or no improvement in performance beyond approximately 9 years of age [18, 25–28]. Cragg and Nation [18] did, however, find continued improvement in response inhibition between younger (5–7 years) and older (9–11 years) school age children when a measure of partial inhibitions was used (instances in which an erroneous NoGo response was initiated but not completed). Finally, most studies comparing adults to children ranging between 6 and 12 years old on the Go/NoGo task have found that adults outperform children, suggesting at least modest performance increments across adolescence [29–31].

One limitation of the Go/NoGo task is that commission errors become relatively infrequent in older children, and this may be one of the reasons that some studies have found little development of response inhibition beyond the early childhood period. The alternative paradigm, the Stop-signal task, differs in terms of the point in time where the prepotent response has to be inhibited; that is to say, whereas in the Go/NoGo task the response is inhibited before response initiation, in the Stop-signal task the response to be inhibited has already been initiated, making the Stop-signal task a somewhat harder task.

Another advantage of the Stop-signal task is that it provides a potentially more sensitive way of measuring the response inhibition process through the so-called ‘stop-signal reaction time’ (SSRT), a derived measure of a person’s speed of inhibition of a motor response despite the absence of overt behaviour [32, 33]. The SSRT is derived from a combination of measurements and is described in more detail in the Procedure section for the Stop-signal Task later in this report. A shorter SSRT, indicates a faster inhibition process. In contrast to the Go/NoGo literature, research employing the Stop-signal task indicates changes in inhibitory performance across the lifespan. This research has found a substantial decrease in SSRT during middle childhood and an increase, of variable magnitude, in old age, suggesting a quadratic, or U-shaped, developmental function across the lifespan [10, 33–37].

A limitation of both the Go/NoGo task and the Stop-signal task is that neither is suitable for very young children. Preschool children have slow reaction times, and it is not always clear whether they understand instructions or are able to maintain them in working memory while performing the task (so-called ‘task set’). Carver et al. [35, 37] found that even with plenty of ‘warning’ (i.e., a short *stop-signal delay*, see the section ‘Stop-signal task (SST)’ below), 4- to 5½-year-olds struggled to inhibit responses in the Stop-signal task, whereas older children improved substantially when given a little extra time to stop. These authors concluded that young children are particularly sensitive to task demands and that more work was needed to make response inhibition tasks like the Stop-signal task age-appropriate for the youngest children.

The Go/NoGo task can be used with children as young as 3 years of age [20], although at this age participants are again very sensitive to task parameters, such as the time given to respond [38], and need to be instructed explicitly not to make the NoGo response [21]. For children younger than 3 years, it becomes increasingly difficult to know whether participants understand and/or can maintain task instructions such as “if-then” rules [39], and this clearly has implications for compliance with the task requirements and how to interpret performance.

In the present report, we were interested in establishing an adequate way to measure response inhibition in toddlers, that is, in children as young as 18 months of age. Although some IC tasks can be used from around 2 years of age, these tasks primarily involve perceptual conflict (for a comprehensive review, see [40]); for example, in the Spatial Conflict task the child has to overcome the tendency to respond based on spatial proximity in order to match two identical images and receive a reward [41, 42] (see also [43]). This type of IC is more similar to interference control, as described by Friedman and Miyake [8], than classically defined response inhibition, which involves the continuous *build-up* of motor prepotency over trials. Perceptual IC tasks also have high working memory and language demands (e.g., in the Spatial Conflict task children are instructed to *match* two images), meaning that they are unsuitable for children under 2 years of age, and that, even in older toddlers, only children with reasonably advanced skills in these domains can perform them. In fact, in their meta-analytic review, Petersen et al. [40] suggested that systematic data missingness for several IC tasks at 25 months of age led to an apparent developmental drop in performance at 30 months. That is to say, rather than this drop being due to developmental decline, Petersen et al. [40] speculated that, at the older toddler age (30 months), less-skilled children, who were not previously able to comprehend the tasks, and were therefore excluded from analyses, could now be included and therefore average IC scores dropped. Similarly, Mulder et al. [39] had to drop a Stroop-like inhibition task from their EF battery for 2½-year-olds because it was too difficult at this young age.

In addition to being able to include the youngest toddlers, we wanted to develop a task that did not need changing in any substantial way to be used across development, i.e., a task that could be increased in difficulty, but was *structurally similar* across different ages. Just as many pre-school IC tasks are too difficult for toddlers, the few infant IC tasks (suitable from 6–9 months of age) that currently exist [44–46] are typically not adequate or are too easy for toddlers. This means that studies of IC development tend to ‘shift’ to a new set of tasks between infancy and toddlerhood, and between toddlerhood and early childhood. For example, Carlson [47] reported on a large data set involving assessment using a range of EF tasks (not exclusively IC tasks) in pre-schoolers aged 2, 3, 4 and 5 to 6 years. Only one out of the 6 tasks deemed suitable at 2 years was also used at 3 years, by which point performance was approaching ceiling on this task. The consequence of this shift in assessment method across age is that we cannot be certain that the same inhibitory function (if even inhibitory) is measured across development, especially given the diversity of definitions and the generally low correlations between tasks measuring different types of IC [8–10, 13, 36]. The lack of structurally equivalent response inhibition tasks that can be used already from early toddlerhood therefore constitutes a significant methodological limitation of the field as it stands at present.

A few IC tasks have been used successfully across the toddler years. These tasks are typically temptation-based, such as delay-of-gratification and prohibition tasks, where the child is asked to not touch, or delay touching, an attractive object [4, 5, 39]. However, such tasks are generally limited to a small number of trials, therefore resulting in reduced variability in terms of individual differences (i.e., either the child can wait or cannot) as well as ceiling effects in older toddlers and pre-schoolers [40]. Perhaps more importantly, there is substantial evidence that this type of task constitutes a ‘hot’ measure of IC, that is, a type of executive functioning that operates in motivationally and emotionally significant contexts, as compared to ‘cool’ aspects of EF, which operate in neutral contexts, e.g., the Go/NoGo task (for review, see [48]). Recent factor analytic work has indicated a better fit of a two-factor model of EF in early childhood, involving two overlapping (r ~ .5) but also separable latent factors: hot and cool EF [11, 39, 49]. Furthermore, hot EF has been shown to have both different neural substrates [6, 50] and different longitudinal outcomes compared to cool EF [11, 39, 49, 51, 52].

As a type of inhibitory control, response inhibition clearly falls within the cool EF domain. As mentioned above, at present, practically all cool IC tasks for toddlers rely on a perceptual conflict to be resolved, with instructions that are too difficult to understand for most children under 2 years of age. Furthermore, in studies with older children, one of the main types of IC investigated is the ability to overcome a prepotent motor response (perhaps most notably in neuroimaging research, see [17]. Therefore, to be able to link IC development across childhood and to study this construct from its earliest emergence, more tasks are needed to cover the early toddlerhood period, particularly tasks which are genuinely comparable to the tasks used in older children. To address this, in the work reported here, we focus specifically on the development of a task which requires response inhibition in the classic sense of involving the inhibition of a repeated motor response, but which can also be used over a relatively wide age range.

The new task, termed the Early Childhood Inhibitory Touchscreen Task (ECITT), was designed to measure the ability to inhibit a prepotent response. The task was presented to the children as an iPad game in which they had to press one of two buttons depending on which one had a ‘happy face’ (smiley) on it. As such, the instructions were simple and required minimal language ability and working memory, the only thing children had to remember was that they needed to press the ‘happy face’. To support toddlers’ understanding of the task requirements, and to maintain attention and motivation, a short animation was played after each correct response. The smiley appeared in one location more often (75%) than the other, thus building up a prepotent response to that particular location (prepotent trials). On a smaller number of trials (25%), the smiley button was switched to the other location (inhibitory trials), requiring the child to inhibit pressing the prepotent location in order to see the animation in the new location. We also developed a faster-paced version for older children and adults (ECITT-A) to be able to investigate the lifespan development of response inhibition using the new task and to more firmly establish validity. Importantly, the toddler and adult versions of the task were structurally very similar. In the adult version we omitted the animations, asked participants to re-centre their response finger between trials, and encouraged them to respond as fast as possible. No other modifications were made to the task, making it essentially the same task, just faster paced.

We investigated the validity, reliability and potential use of the new task in three independent studies, as well as in additional studies reported in the Supporting Information, with participants ranging in age from 15 months to 84 years. As such we had two inter-related aims. Our primary aim was to establish that the new task worked as intended. For example, it was key to establish that there was an effect of trial type (inhibitory vs. prepotent) on accuracy and reaction time. However, given that there is very little knowledge about the development of response inhibition in toddlerhood, we also discuss our age-related cross-sectional and longitudinal findings in terms of their implications for early IC development.

In all studies, we predicted that participants would make more errors and produce slower correct responses on inhibitory trials compared to prepotent trials. We did, however, expect that adults would perform at ceiling in terms of accuracy, especially younger adults. We also conducted internal consistency analyses to establish that participants’ performance was consistent across trials within a session. Based on the logic of the task and the previous literature, we had specific predictions regarding the effect of age on inhibitory performance. In Study 1, we compared performance of a group of 24-month-olds to a group of 30-month-olds. We predicted that, overall, toddlers of both ages would perform worse on the inhibitory trials than on the prepotent trials, as measured by accuracy and reaction time (RT). We also expected to see a significant improvement in inhibitory performance from 24 to 30 months of age. In our next study, Study 2, we assessed a group of toddlers longitudinally at 18, 21, and 24 months of age. We again predicted improvement in inhibitory performance with age. We also expected that individual differences in inhibitory performance would be stable across ages, i.e., that toddlers’ performance would be correlated between the three assessment points (such longitudinal correlations would also approximate a lower bound for test-retest reliability). Finally, Study 3 took a lifespan developmental perspective by comparing mid-primary school children, young adults and older adults on the faster-paced adult version of the task (ECITT-A). In accordance with previous work on the Stop-signal task [10, 33, 34, 36], we predicted a quadratic (i.e., u-shaped) relation between age and inhibitory performance as measured by the ECITT-A. We also directly correlated ECITT-A performance with Stop-signal performance to further establish the construct validity of the new task.

## Supporting information and open materials

All Supporting Information relating to this article is openly available on https://osf.io/ytfdp/. These materials include: additional illustrations of the stimuli, supplementary analyses, the data reported in this article, SPSS syntax to run the analyses, and the ECITT software code. Furthermore, a substantial amount of data was collected in *additional studies* that we have left out of the main report for succinctness, but which can be accessed via the OSF archive. These additional studies and analyses include:

- S1 Supporting Information: Pilot Study of 15 toddlers aged 20–28 months.
- S2 Supporting Information: Reliability of ECITT trial accuracy and reaction time
- S3 Supporting Information: Data from a small group of 15-month-olds. These are a subset of the longitudinal participants in Study 2. Due to the small amount of data (the task was introduced at the end of the 15-month testing wave), this data was not included in the main analyses.
- S4 Supporting Information: ECITT performance in relation to Reverse Categorisation and Prohibition task performance in Study 2.
- S5 Supporting Information: Additional analyses of the Stop-signal task data collected in Study 3.
- S6 Supporting Information: Lifespan study run at public engagement events (Study 4, see Additional Studies section): 140 participants, ranging in age from 17 months to 71 years.
- S7 Supporting Information: Regression analyses of the relationship between age and inhibitory performance which combine all cross-sectional data collected across Studies 1, 3, 4 and the Pilot Study (*N* = 300), including analyses split by task version (ECITT and ECITT-A).

All materials in the OSF archive fall under a CC-BY Attribution 4.0 International license, and the current report must be cited if these materials are used for any commercial or non-commercial purpose.

## Study 1

### Study overview and predictions

In Study 1, we hoped to establish an effect of ECITT condition in toddlers aged 24 and 30 months. We predicted overall lower accuracy on inhibitory trials compared to prepotent trials, and slower median RT on correct inhibitory trials compared to correct prepotent trials. Preliminary pilot data had already indicated that an effect of condition was likely to be present, at least in terms of accuracy (for details on the Pilot Study, see S1 File). In addition to a main effect of condition, we expected developmental progression specifically within the inhibitory domain, i.e., beyond general improvements in performance with age. We therefore predicted that (1) 24-month-olds would make relatively more errors in the inhibitory condition compared to the prepotent condition than would 30-month-olds, and (2) 24-month-olds would be significantly slower on correct inhibitory trials compared to correct prepotent trials than would 30-month-olds.

### Method

#### Participants

Participants consisted of two groups of toddlers: a group of 24-month-olds recruited at the Oxford Babylab in Oxford, UK, and a group of 30-month-olds recruited at the Birkbeck Babylab in London, UK.

*Oxford Babylab sample (24-month-olds)*. Thirty-nine toddlers, 17 girls and 22 boys, were recruited through the Oxford Babylab volunteer database and advertisements placed on the lab’s Facebook page. Most families on the Oxford Babylab volunteer database were recruited on the maternity ward at a regional hospital in Oxford. Other families signed up directly via the lab’s webpage. All child participants were born full-term (at least 36 weeks gestation) and none of them had any diagnosed developmental disorders or other serious health issues. One child (a girl) refused to touch the screen and was therefore excluded from analyses. Demographic data for the sample (and other samples in this report) are presented in Table 1. At the visit, 24-month-olds were on average 744 days old (*SD* = 17, Range = 710–771). The study received ethical approval from the University of Oxford Medical Sciences Interdivisional Research Ethics Committee (Ref. No. R39996/RE001). A parent or guardian provided written informed consent.

**Table 1:** Demographic information for participants in Studies 1, 2 and 3.

Sample	Birkbeck Toddlers	Oxford Toddlers	VT Toddlers (longitudinal)	Children	Young Adults	Older Adults
*N*	47(44)*	39(38)**	38	27	17	20
	*Mean or %*	*Mean or %*	*Mean or %*	*Mean or %*	*Mean or %*	*Mean or %*
Age (months/years)	29.68 mths	24.03 mths	18.27, 21.16 & 24.20 mths	7.93 yrs	22.88 yrs	69.65 yrs
Sex						
% Female	56.82% (25/44)	42.11% (16/38)	44.74% (17/38)	66.67% (18/27)	82.35% (14/17)	55.00% (11/20)
Ethnicity						
White (British, Irish, American or Other)	79.55% (35/44)	76.32% (29/38)	89.47% (34/38)	--	--	--
Asian	0.00% (0/44)	0.00% (0/38)	2.63% (1/38)			
Afro-Caribbean	2.27% (1/44)	0.00% (0/38)	0.00% (0/38)	--	--	--
Other Black Background	0.00% (0/44)	0.00% (0/38)	0.00% (0/38)	--	--	--
Mixed—White and Asian	9.09% (4/44)	5.26% (2/38)	0.00% (0/38)	--	--	--
Mixed—White and Black	2.27% (1/44)	2.63% (1/38)	7.89% (3/38)	--	--	--
Other Mixed Background	6.82% (3/44)	2.63% (1/38)	0.00% (0/38)	--	--	--
Not provided	0.00% (0/44)	13.16% (5/38)	0.00% (0/38)	--	--	--
Highest level of education (adult participants)						
GCSEs	--	--	--	--	0.00% (0/17)	70.00% (14/20)
A-levels	--	--	--	--	82.35% (14/17)	0.00% (0/20)
Degree / Higher National Diploma	--	--	--	--	17.65% (3/17)	15.00% (3/20)
Postgraduate degree / Doctorate	--	--	--	--	0.00% (0/17)	0.00% (0/20)
Not provided	--	--	--	--	0.00% (0/17)	15.00% (3/20)
Total years in education	--	--	--	--	16 (17/17)	12.78 (18/20)
Household Income						
Under £15,000	2.27% (1/44)	5.26% (2/38)	--	3.70% (1/27)	29.41% (5/17)	20.00% (4/20)
£15,000 - £30,000	0.00% (0/44)	7.89% (3/38)	--	25.93% (7/27)	35.29% (6/17)	15.00% (3/20)
£30,000 - £45,000	0.00% (0/44)	7.89% (3/38)	--	14.81% (4/27)	23.53% (4/17)	50.00% (10/20)
£45,000 - £60,000	9.09% (4/44)	7.89% (3/38)	--	7.40% (2/27)	5.88% (1/17)	10.00% (2/20)
Over £60,000	68.18% (30/44)	50.00% (19/38)	--	0.00% (0/27)	0.00% (0/17)	0.00% (0/20)
Not provided	20.45% (9/44)	21.05% (8/38)	--	48.15% (13/27)	5.88% (1/17)	5.00% (1/20)
Maternal Characteristics						
Age (years)***	37.00 (36/44)	36.03 (34/38)	31.21 (38/38)	35.00 (20/27)	--	--
Total years in education	17.86 (44/44)	17.27 (30/38)	17.29 (38/38)	14.42 (19/27)	--	--

*Note*. Numbers in brackets indicate the frequency of a category/characteristic out of the total participant sample. *Three participants were excluded prior to analysis (2 boys and 1 girl), one due to experimenter error and two due to no video being recorded during the session; these participants are not included in the remainder of the table. **One participant (a girl) refused to engage with the Early Childhood Inhibitory Touchscreen task and is not included in the remainder of the table. ***For the Oxford and Birkbeck toddlers, maternal age was reported at the time of the test session (24-month and 30-month session, respectively); for the Virginia Tech (VT) toddlers, maternal age was reported at the child’s birth.—Indicates that this type of demographic data was not collected in this sample. (A larger version of this table can be found in S1 Table).

*Birkbeck Babylab sample (30-month-olds)*. Forty-seven toddlers, 26 girls and 21 boys, who were participating in a longitudinal study on the development of mimicry at the Birkbeck Babylab completed the ECITT during their 30-month visit. Participants were recruited when the children were 4 months old through the Birkbeck Babylab database, which includes details of families who have voluntarily signed up for participation in studies on infant development. Participants had been to the lab for testing at 4, 11, 18 and 24 months before the current session, although 4 of them started their participation at 18 months. All participants were born full-term (minimum 36 weeks gestation), and none had been diagnosed with a developmental disorder or suffered other serious health issues. None of the previous sessions involved any measures of inhibitory control. Three participants were excluded prior to analysis (2 boys and 1 girl): one due to experimenter error and two due to no video being recorded during the session. Demographic data for the sample are presented in Table 1. At the 30-month visit, the children were on average 919 days old (*SD* = 11, Range = 897–943). The study was approved by the Department of Psychological Sciences Research Ethics committee at Birkbeck (Ref. No. 141573). A parent or guardian provided written informed consent.

#### Task order

In the Oxford Babylab sample, 24-month-old toddlers completed one task before the ECITT, which also functioned as a warm-up to using the touchscreen. The task involved a cartoon butterfly being presented on the iPad, which was then left on the table in front of the toddler to see how long they took to touch the screen. (Results from this task do not form part of the present report.) Touching the cartoon butterfly elicited a pleasant sound and made the butterfly flutter to a different location on the screen. After a short play with the butterfly, the session continued to the administration of the ECITT. In the Birkbeck Babylab sample of 30-month-olds, toddlers were also allowed to play with the butterfly (or a similar game with a cartoon frog), but as a simple warm-up, not a formal task. When the child was comfortable interacting with the screen, the ECITT was administered. The ECITT was the first tablet-based task after a series of monitor-based tasks and one behavioural task relating to social cognition. (Data from these other tasks are the topic of several separate articles and are therefore not reported here.)

#### Apparatus and stimuli

**Fig 1:**
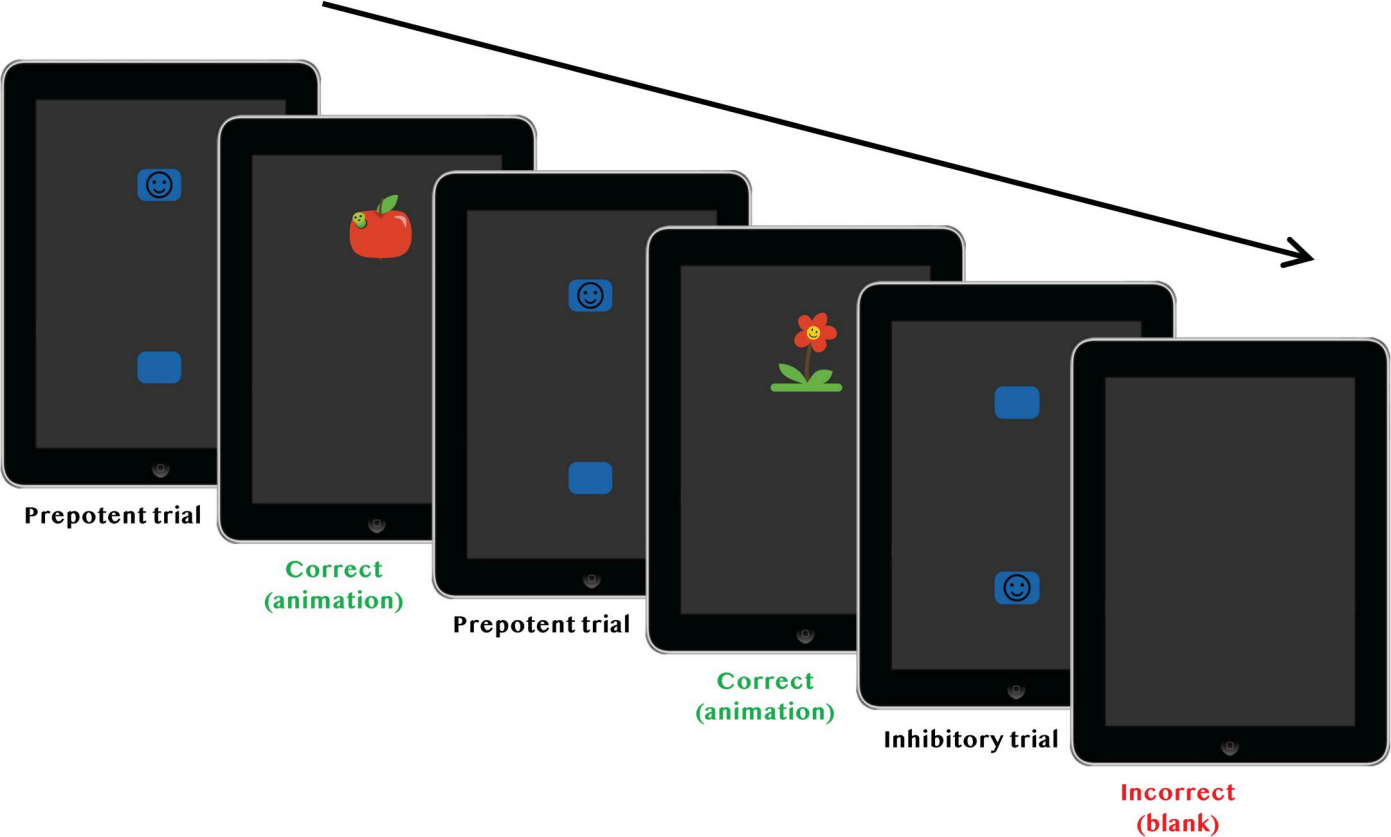
Illustration of the Early Childhood Inhibitory Touchscreen Task (ECITT).

An illustration of the stimuli and procedure used in the ECITT is presented in Fig 1. Stimuli were presented on an Apple iPad tablet (the ‘responder’), with a screen size of 9.7 inches and a resolution of 2048 × 1536 pixels. The experimenter controlled stimulus presentation on the iPad via a smartphone (the ‘controller’) using a wi-fi network. The ECITT step-by-step testing protocol that experimenters used during testing can be found in S1 Protocol (see ‘Software access’ below for information on how to access the software).

The responder was used in a portrait orientation. Stimuli consisted of two 17 × 24 mm, blue, rectangle-shaped touchscreen buttons, positioned 81 mm apart vertically (see S1 Fig). A 14-mm diameter simple “smiley” icon was presented in either the top or bottom button. Stimuli were displayed against a dark grey background. On practice trials, a single blue button with a smiley on, of the same dimensions, was presented in the centre of the screen. The touch sensitive area included a small area around the blue buttons, so that the total response sensitive area was 44 × 44 mm. The response sensitive area was slightly larger than the buttons to accommodate for young children’s often slightly inaccurate touches. Toddlers’ behaviour was recorded using a video camera placed on a tripod stand behind the toddler to allow for offline coding of responses. Short cartoon animations (e.g., a dancing elephant, a worm peeking out of an apple) combined with sound effects were played after each successful response.

The iPad recorded the accuracy and reaction time (in milliseconds) of each response. These were later checked and corrected (where necessary) in the offline coding (see below).

#### Software access

The code for the ECITT software (called the ‘ECITT Web App’) can be downloaded for free at https://figshare.com/articles/software/ECITT_Web_App/13258814. A person with programming expertise will need to set up the software on a web server. The task is also available on https://ecitt.app and can be tested with our guest account. (Username: guest; Password: demo). This account is not suitable for data collection as data will be publicly available. Please read the guidance on https://ecitt.app for further details.

#### Procedure

Toddlers sat on their caregiver’s lap throughout the task. A single experimenter administered the task by holding the iPad in front of the child on each trial using a case with a hand strap.

*Practice trials*. The experimenter attracted the child’s attention, held the iPad out of the child’s reach, and demonstrated pressing the smiley on the single central blue button. As a result, the short animation played while the child was watching. Another trial was then presented, and the child was encouraged to “press the happy face”. If the child was reluctant to press the button, the experimenter demonstrated again until the child was happy to press the button without assistance.

*Experimental trials*. After the practice, a single block of 32 experimental trials was presented. Before test trials began, the caregiver was instructed to avoid pointing to or labelling the buttons. Two buttons were presented on the screen and the child was instructed by the experimenter to “press the happy face”. If the child pressed the correct button, the animation played. Animations lasted between 3.75 and 4 s and ended with a return to the button display (i.e., the next trial started immediately after the animation). If the child pressed the incorrect button, the buttons disappeared from the screen, and the screen was then left blank for 1 s before the next trial started. The smiley appeared in the prepotent location on 24 trials (75% of trials) and in the inhibitory location on 8 trials (25% of trials). The experimental block always started with at least 3 prepotent trials (to establish the prepotent response). On the first test trial, the experimenter pointed to the correct response location–this was done to ensure that the child responded to the prepotent location, which was important for establishing the response prepotency from the outset (the first trial was subsequently discarded from analysis). Trial presentation was automatically randomised at the start of each experimental block using the following constraints: max. 5 prepotent and max. 2 inhibitory trials in a row (with the additional constraint that the first three trials were always prepotent). Whether the prepotent location was at the top or bottom was manually counterbalanced across participants.

Between trials the experimenter took the iPad slightly out of reach to avoid toddlers tapping the screen excessively (a common behaviour in this age group). The screen was then moved back in front of the child as soon as the next trial started to ensure that the child could respond immediately. This procedure avoided the loss of multiple trials due to excessively short reaction times (for details, see the section “Video coding and data cleaning” below).

Toddlers were not put under any time pressure to respond during the task, in order to avoid stress and non-compliance impacting on their performance. The instruction to “press the happy face” was repeated as needed throughout the session, and the experimenter also frequently provided encouraging comments when the reward animation played (e.g., “Look, there’s the dancing elephant again”). This ensured a high level of participant engagement.

#### Data analysis

*Data processing and statistical analysis*. Analyses were carried out in Microsoft Excel and SPSS version 27 and 28 (IBM, 2020, 2021). An alpha level of *p* < .05 was used as the threshold for statistical significance. In all analyses, tests of significance were two-tailed.

*Video coding and data cleaning*. Very young children regularly exhibit extremely short (e.g., repetitive tapping) or long reaction times (e.g., disengaging from the task). To ensure that the data were as clean as possible when comparing performance in the two conditions (prepotent and inhibitory) and between age groups, trials were coded manually from the videos. Coders checked that responses were recorded correctly by the iPad and, if not, corrected them accordingly (this typically happened if a touch response was not detected by the iPad). In addition, a set of criteria for each trial was applied to exclude responses that were unlikely to accurately reflect performance. All trials with a reaction time (RT) shorter than 300 ms were coded as invalid and excluded from analysis. This cut-off was chosen as, in nearly all instances where RT < 300 ms, the child had their finger very close to one of the response locations at trial onset. It follows that if the child’s finger was on one of the buttons (or within the touch sensitive area) at trial onset, the trial was always coded as invalid. Occasionally the parent or experimenter intervened during a trial. If the intervention simply consisted of encouragement to touch the screen, the trial was retained. However, if the parent or experimenter pointed directly to one of the response locations or otherwise clearly indicated the correct or incorrect response (e.g., verbally: “touch the one at the top”), the trial was coded as invalid and excluded. Other instances of parent intervention included nudging towards a response or preventing the child from making an incorrect response. Finally, accidental touches were excluded, e.g., if a child brushed their hand accidentally over the screen while turning to the parent.

For accuracy analyses, all trials retained after the above exclusions were included. However, for RT analyses, two additional criteria were applied: (1) the response on the trial had to be correct and (2) RT had to be less than 5000 ms. The latter criterion was applied to avoid including excessively long RTs, e.g., when a child got distracted for a longer period of time.

Data from all participants in Study 1 (*N* = 82, one 30-month-old was later excluded due to experimenter error) and the Pilot Study (*N* = 15, see S1 File) were coded, and a subset of 31% of these (including the entire Pilot sample) were coded by two independent coders to establish adequate intercoder reliability. Intercoder reliability based on 30 participants (1050 trials) was excellent (*κ* = .85 for validity; *κ* = .98 for accuracy).

*Exclusions*. A small number of toddlers did not understand or cooperate with the task instructions, therefore a criterion of more than 60% correct on prepotent trials was applied for inclusion in the analyses. This was done because if the child was performing randomly on the prepotent trials, a prepotent response tendency would not be built up on these trials, meaning that performance on the task was unlikely to reflect response inhibition. This performance pattern happened rarely: 5 out of 81 participants (two 24-month-olds and three 30-month-olds) were excluded because they were not over 60% correct on the prepotent trials.

*Dependent measures*. For the accuracy analysis, the percentage correct was calculated separately for the inhibitory and prepotent condition for each child. For the RT analysis, the median RT was calculated separately for the two conditions for each child. Median RT was used in the analyses, as is common in developmental research (e.g., [53]), because medians are less distorted by outliers. Accuracy and RT were analysed in 2 × 2 mixed ANOVAs with Age as a between-subjects factor and Condition as a within-subjects factor.

*Individual performance measures*. To enable us to look at developmental progression in inhibitory performance with age, two inhibitory scores were calculated. For accuracy, the inhibitory score was calculated as follows: prepotent condition % correct minus inhibitory condition % correct. A higher accuracy difference (AccD) score indicated that accuracy in responding to inhibitory trials was lower compared to prepotent trials; therefore, the larger the (positive) difference, the *poorer* inhibitory control. For RT, a difference score was again derived as an indicator of inhibitory performance: median RT on inhibitory trials minus median RT on prepotent trials (RT difference: RTD). A higher RTD score was taken to indicate *poorer* inhibitory control. To compare age groups, Welch’s t test was used (with AccD or RTD as the dependent variable) because this test of mean differences between two independent groups is more robust than Student’s t test when sample sizes and group variances are unequal, which is often the case in psychological research [54].

### Results

#### ECITT accuracy

**Fig 2:**
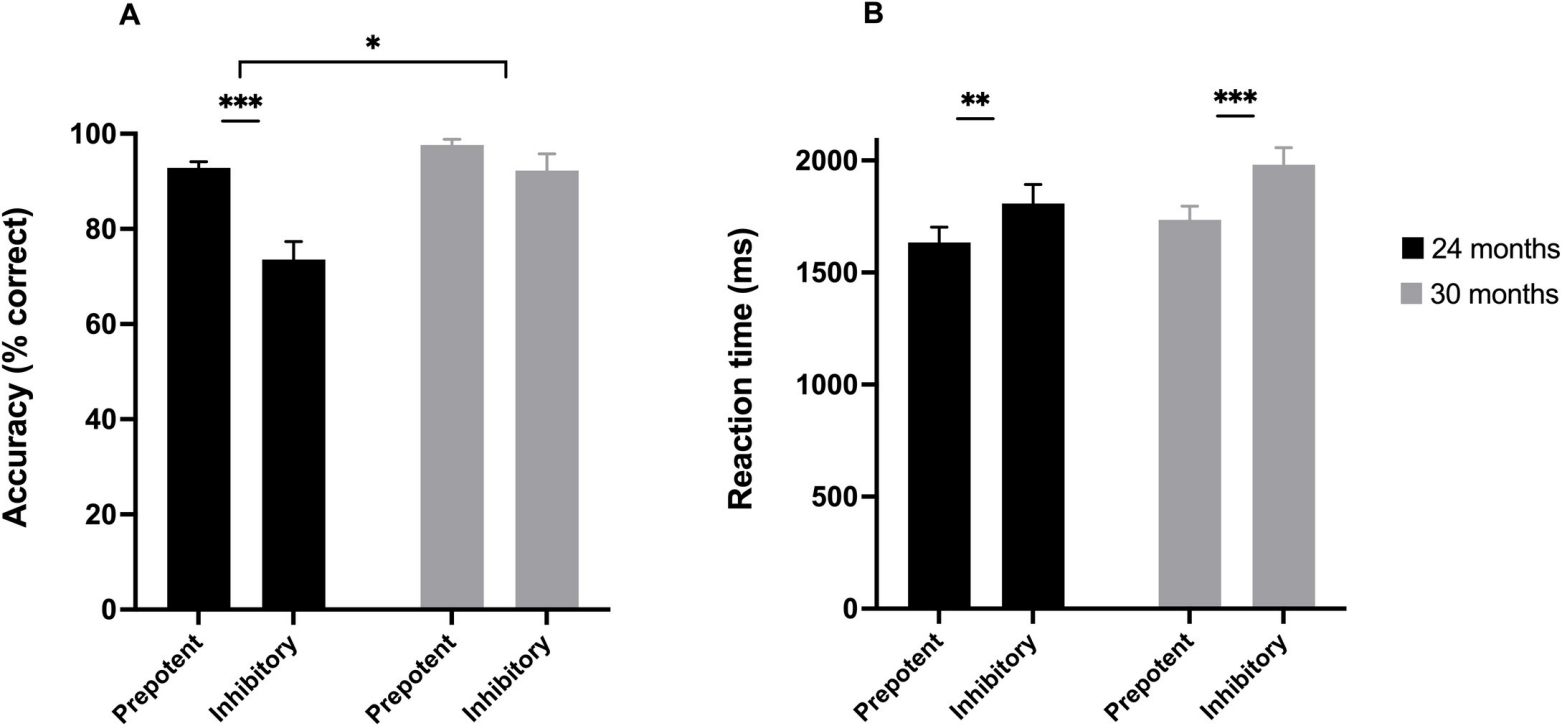
(A) Mean accuracy (% correct) and (B) mean median reaction time in milliseconds for inhibitory and prepotent trials in the Early Childhood Inhibitory Touchscreen Task at 24 and 30 months of age. The bracket at the top in Fig 2A indicates the significant cross-age comparison of mean accuracy difference (AccD) score. Error bars indicate the standard error. *** *p* < .001, ** *p* < .01, * *p* < .05.

Mean accuracy on prepotent and inhibitory trials in 24- and 30-month-old children is illustrated in Fig 2A. Data were analysed using a 2 × 2 mixed ANOVA. The results indicated significant main effects of Age, F(1,74) = 15.39, p < .001, η_p_^2^ = .17 and Condition, F(1,74) = 24.57, p < .001, η_p_^2^ = .25. Overall, the children performed better on the prepotent trials than on the inhibitory trials, and 30-month-olds performed better on the task than 24-month-olds. The Age × Condition interaction was significant, F(1,74) = 7.83, p = .007, η_p_^2^ = .10, indicating a differential effect of Condition at the two ages. To confirm our prediction that the developmental progression was primarily due to the younger toddlers performing particularly poorly in terms of inhibitory control (or conversely, the older toddlers having mastered a higher level of inhibitory control), we ran a planned comparison of the AccD score in the two age groups. The comparison indicated that 24-month-olds performed particularly poorly on the inhibitory trials relative to the prepotent trials (M = 19.29%; SD = 29.72%) compared to the 30-month-olds (M = 5.37%; SD = 10.64%), t(41.43) = 2.63, p = .012, d = 0.64.

As the intention is for the AccD to be useful as an index of individual differences in response inhibition, it is important to consider that, especially in younger toddlers with low performance on the prepotent trials, the AccD could provide an over-estimate of their inhibitory performance. For example, a toddler performing at 62.5% correct in prepotent trials and 50% correct in inhibitory trials (AccD = 12.5%) is clearly not performing as well as a toddler who is 100% correct in prepotent trials and 87.5% correct in inhibitory trials (AccD = 12.5%); in fact, the second toddler builds up a stronger prepotency on the (correct) prepotent trials, which they nevertheless manage to overcome (therefore demonstrating better response inhibition). This can be corrected for by dividing the AccD by % correct in prepotent trials (a similar method to the one used for calculating Spatial Conflict interference scores, see [41]). Running the planned age comparison using adjusted AccD scores led to nearly identical results. Twenty-four-month-olds had a larger adjusted AccD (*M* = 20.61%; *SD* = 31.31%) than 30-month-olds (*M* = 5.75%; *SD* = 12.13%), *t*(42.67) = 2.64, *p* = .011, *d* = 0.65. This indicates that, in this age group, the AccD is a suitable measure of inhibitory performance, although, depending on the population, the adjusted AccD may be preferred.

#### ECITT reaction time

Two additional toddlers (both 24-month-olds) were excluded from the RT analyses, as they did not have any correct responses on inhibitory trials. Mean median RT (ms) on prepotent and inhibitory trials in 24-month-old and 30-month-old children is illustrated in Fig 2B. A 2 × 2 mixed ANOVA indicated a significant effect of Condition, F(1,72) = 37.77, p < .001, η_p_^2^ = .34, but no significant effect of Age, F(1,72) = 1.99, p = .162, η_p_^2^ = .03. There was also no interaction between Age and Condition, F(1,72) = 1.11, p = .295, η_p_^2^ = .02: both 24-month-olds and 30-month-olds made slower correct responses on inhibitory trials (p = .001, η_p_^2^ = .14 and p < .001, η_p_^2^ = .29, respectively). Accordingly, the planned comparison between RTD in 24-month-olds and 30-month-olds was not significant, t(70.65) = 1.06, p = .29, d = 0.25.

#### Internal consistency

A full overview of internal consistency (Cronbach’s alpha) in Studies 1–3 can be found in S2 File. It was not feasible to analyse internal consistency of trial RT in toddlers (for details, see S2 File), so these analyses focused on inhibitory trial accuracy, which is most likely to tap into the construct of interest (i.e., response inhibition). When only participants with 8 valid inhibitory trials were included (82.9% of 24-month-olds and 92.9% of 30-month-olds), Cronbach’s alpha values for inhibitory trial accuracy were high in both 24-month-olds (⍺ =. 0.86) and in 30-months-olds (⍺ = 0.75). When only 6 trials were included in the internal consistency analysis, all children could be included, but alpha dropped substantially in the 30-months-olds (⍺ = 0.44). This suggests that in older toddlers (where performance is high), 8 trials are needed to obtain reliable individual differences in inhibitory performance. It is worth noting that most 30-months-olds (92.9%) had all 8 inhibitory trials available for analysis.

### Discussion

A direct comparison between a group of 24-month-olds and a group of 30-month-olds indicated that the younger toddlers made significantly more errors than the older toddlers. Furthermore, as predicted, a particularly substantial improvement with age was observed in the inhibitory condition, suggesting that the ECITT is sensitive to improvements in inhibitory control even at this young age. Performance on inhibitory trials was highly consistent within sessions, although, in older toddlers, 8 inhibitory trials were needed to retain a high level of internal consistency. Toddlers in both age groups also showed sensitivity to the inhibitory demand of the task in their reaction times–on correct inhibitory trials they slowed down significantly compared to correct prepotent trials. However, against our prediction, no selective decrease in RT on inhibitory trials compared to prepotent trials (which would indicate an increasingly faster inhibition process) was observed with age. Therefore, although the overall RT difference between conditions validates the inhibitory demand of the task, these results suggest that RT might not pick up age differences as well as accuracy in toddlers. One possible interpretation is that both younger and older toddlers are *broadly* able to slow down on successful inhibitory trials, but that the variability of these reaction times is too high to discern developmental change–perhaps it is only as children start producing faster and more consistent motor responses that these differences become apparent. The lack of inhibition-specific age progression in RT found here in toddlers is similar to findings with perceptual IC tasks in 2- to 6-year-old children; these have indicated that the ability to modulate reaction time in response to inhibitory demands typically emerges later than improvements in accuracy [42, 53]. Further research is needed to establish when reaction time becomes a suitable measure to assess developmental change.

A limitation of Study 1 is that the two age groups were tested in different labs. The participant samples were recruited from two major cities in South East England, and the two groups of children did not differ in terms of maternal years in education, *t*(47.68) = -0.84, *p* = .40, a commonly used proxy for socio-economic status. Nevertheless, we cannot rule out subtle differences between the samples that could have contributed to the condition and age effects we observed in Study 1. Furthermore, no cross-sectional study can address change over time, it can only provide a snapshot of what children can do, at the group level, at a particular age. In Study 2 we addressed this question by assessing a group of toddlers longitudinally using the ECITT.

## Study 2

### Study overview and predictions

In Study 2, we investigated the longitudinal development of performance on the ECITT between 18 and 24 months of age. This allowed us to rule out performance differences between age groups being due to unmeasured characteristics of those groups. With this study, we also hoped to demonstrate that the ECITT can be used with toddlers younger than 24 months of age, as presently the lower age boundary for most inhibitory control tasks is 2 years. As in Study 1, we predicted a main effect of condition, that is, lower accuracy and longer correct RT on inhibitory trials compared to prepotent trials across all ages. We also predicted that toddlers would show substantially more improvement in their accuracy on inhibitory trials with age than on prepotent trials, thereby showing developmental progression specific to the inhibitory condition. We were less certain that we would observe developmental progression in terms of RT, as Study 1 indicated a stable RT difference between conditions across the older toddler ages.

### Method

#### Participants

Thirty-eight toddlers, 17 girls and 21 boys, who were participating in a longitudinal study on the development of executive functions in toddlerhood at the CAP Lab at Virginia Tech in the United States, completed the ECITT during their 18-, 21- and 24-month visits to the lab. A subset of 11 of these participants was also administered the ECITT at 15 months (the ECITT was introduced towards the end of this data collection wave). The 15-month data set was so small that we excluded it from the analyses reported below, but we report these results in S3 File. Children in the toddler study were a subset of a larger group of 48 infants, which was assessed monthly from 5 to 12 months of age. During the infant (5 to 12 month) phase, the study focused on two visual inhibitory control tasks: the looking A-not-B and Freeze-Frame tasks [55, 56] (these data are the topic of separate manuscripts). At the completion of the 12-month visit, families were invited to continue participation by also becoming part of the toddler study. Participants were recruited as infants via announcements on the University daily email, local listservs, the lab Facebook page, and the lab participant database. Demographic data for the sample are presented in Table 1. At the 18-month visit children were on average 556 days old (*SD* = 6, Range = 544–569), at the 21-month visit children were on average 644 days old (*SD* = 7, Range = 630–664), and at the 24-month visit children were on average 736 days old (*SD* = 5, Range = 722–752). The toddler study was approved by BRANY Commercial IRB (protocol VT18-647-568). A parent or guardian provided written informed consent.

#### Task order

Six executive function tasks, broadly assessing inhibitory control, working memory and cognitive flexibility skills, were administered at the 18-, 21-, and 24-month visits, in the same order for each visit. The ECITT was always administered second to last in the task sequence and before the only ‘hot’ EF task in the battery (the ‘Wand’ Prohibition task).

#### Apparatus, stimuli and procedure

Toddlers sat in a highchair with their parent seated to their right and within view. The apparatus, stimuli and procedure were the same as in Study 1, with the exception that, if the child pressed the incorrect button, nothing happened. We made this small change because, on rare occasions, toddlers found the disappearance of the blank button rewarding, or appeared to press it repeatedly to get through the task faster (non-compliance). By making the blank button completely unresponsive, there was no reward associated with it, and the next trial would not be presented before the child made the correct response. This may have reduced the inhibitory demand a little (because the correct inhibitory response had to be made after an incorrect response to the prepotent location on an inhibitory trial), but we hoped that this small change would allow us to obtain data from almost all children.

The prepotent location was counter-balanced between participants at the first visit, and each child kept their originally allocated prepotent location for the later visits.

#### Data analysis

*Data processing and statistical analysis*. This was the same as for Study 1.

*Video coding*, *data cleaning and exclusions*. The video coding protocol for Study 2 was the same as described for Study 1, although, due to the inclusion of younger toddlers, the protocol was slightly refined to make the description of the different invalidity codes more precise (for the full coding protocol, see S2 Protocol). All data in Study 2 were coded by a highly trained coder. Twenty-four sessions (767 trials) were independently double-coded by a second trained coder. These sessions were distributed such that 6 sessions at each of the ages (15, 18, 21 and 24 months) were coded, with different children coded at each age to avoid any individual child’s data being over-represented in the reliability set. Intercoder reliability was excellent for both validity (*κ* = .93) and accuracy (*κ* = .94). Reaction time corrections were needed on 94 trials in the reliability set (only valid trials with a correct response had RT corrected (when needed), as RT for incorrect responses was not analysed) and coding of these was also highly reliable (intra-class correlation (single measures) = .90). Three exclusions (out of 95 sessions, i.e., 3%) were made where a child was less than 60% correct on the prepotent trials in one of their sessions. One child fell below this criterion at 15 months and 21 months (note that the 15-month data are presented in S3 File), and another child fell below the criterion at 18 months.

*Dependent measures and statistical design*. Accuracy and RT measures were calculated in the same way as in Study 1. Accuracy and RT were then analysed in 2 × 3 repeated-measures ANOVAs with Age and Condition as within-subjects factors. These were followed up with planned comparisons of the AccD score at the three ages, with the expectation that we would see improvement in AccD (represented by lower scores) with age. Results from Study 1 indicated no improvement in RTD with age, so we had no specific prediction regarding this index at these younger toddler ages. Age-to-age paired-samples t tests were also carried out, with AccD/RTD as the dependent measure. This was done to check that results of the repeated-measures ANOVAs were not influenced by attrition at individual ages. That is to say, in the repeated-measures ANOVA, a child with a single missing data point at one age is excluded from all three age comparisons, whereas in t tests comparing two individual ages, missing data at the third age will not lead to exclusion from the comparison at the two other ages; therefore, the t tests allowed us to confirm the results from the ANOVAs with minimal data attrition. Cronbach’s alpha was used to assess internal consistency (see S2 File). Finally, the AccD and RTD were used as individual scores in correlation analyses assessing longitudinal stability in performance on the ECITT.

### Results

#### ECITT accuracy

**Fig 3:**
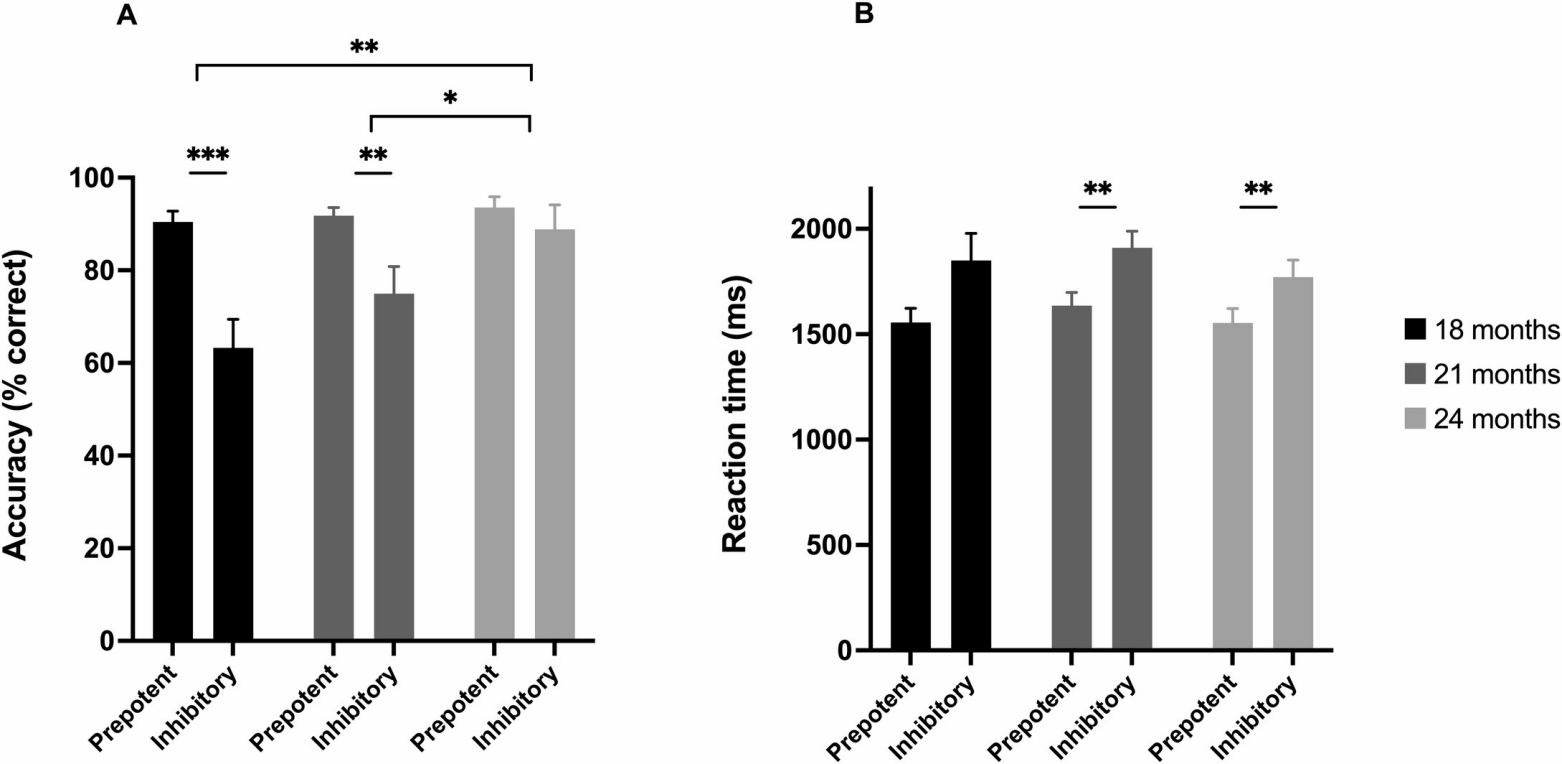
(A) Mean accuracy (%) and (B) mean median reaction time in milliseconds for inhibitory and prepotent trials in the Early Childhood Inhibitory Touchscreen Task, assessed longitudinally at 18, 21 and 24 months of age. The brackets at the top in Fig 3A indicate the significant cross-age comparisons of mean accuracy difference (AccD) score. Error bars indicate the standard error. *** *p* < .001, ** *p* < .01, * *p* < .05.

The 2 × 3 repeated-measures ANOVA indicated significant main effects of Age, *F*(2,36) = 7.53, *p* = .002, *η*_*p*_^*2*^ = .30 and Condition, *F*(1,18) = 15.93, *p* = .001, *η*_*p*_^*2*^ = .47. The Age × Condition interaction was also significant, *F*(2,36) = 8.95, *p* = .001, *η*_*p*_^*2*^ = .33, indicating a differential effect of condition with age. The mean accuracy on prepotent and inhibitory trials at 18, 21 and 24 months is illustrated in Fig 3A. To follow-up on the interaction between Age and Condition, a repeated-measures one-way ANOVA with Age as a single within-subjects factor (with 3 levels) and the AccD as the dependent measure was carried out, and the posthoc tests of age-to-age differences were taken to indicate which ages differed from each other. These planned comparisons indicated that, at 24 months of age (AccD: *M* = 4.75%; *SD* = 18.71%), children performed significantly better than at 18 months (*p* = .001; AccD: *M* = 27.15%; *SD* = 25.22%) and 21 months (*p* = .014; AccD: *M* = 16.85%; *SD* = 22.19%) of age. Children’s AccD scores did not improve significantly between 18 and 21 months of age (*p* = .075). Separate paired samples t tests, carried out to include participants with missing data at one of the age points, confirmed these results: Children improved between 18 and 24 months, *t*(18) = 3.78, *p* = .001, *d* = 0.87, and between 21 and 24 months, *t*(23) = 2.35, *p* = .028, *d* = 0.48. However, AccD scores did not improve significantly between 18 and 21 months, *t*(22) = 1.79, *p* = .087, *d* = 0.37.

#### ECITT reaction time

It was not possible to calculate the RTD for 4 individual sessions (one at 18 months, two at 21 months and one at 24 months) because the child responded incorrectly on all inhibitory trials in these sessions. The 2 × 3 repeated-measures ANOVA indicated that there was only a significant effect of Condition, *F*(1,15) = 26.73, *p* < .001, *η*_*p*_^*2*^ = .64. The paired-samples t tests comparing the RTD between each age was consistent with this (all *p*s > .20). This indicates that children were slower to respond on correct inhibitory trials than on correct prepotent trials, but there was no developmental change in this difference. The mean median reaction times on prepotent and inhibitory trials at 18, 21 and 24 months of age are illustrated in Fig 3B.

#### Internal consistency

Cronbach’s alpha values for inhibitory trial accuracy at 18, 21 and 24 months can be found in (Table 1A) in S2 File. All alpha values (both for participants with 8 and participants with 6 inhibitory trials available) were above 0.60 and ranged between 0.64 and 0.81. The mean alpha value across age for 8 inhibitory trials (inclusion of 77.3% of participants) was 0.75, and the mean alpha value for 6 inhibitory trials (inclusion of 96.1% of participants) was 0.70.

#### Individual performance correlations

We used Pearson correlations to establish whether ECITT accuracy (AccD) and RT (RTD) performance was stable across age. The AccD longitudinal correlations can be seen in Table 2. One participant had an RTD score more than 3 standard deviations above the mean at 18 months (for details, see next section). S2 Table presents AccD correlations after exclusion of this participant (results were similar to when the participant was included). As can be seen from Table 2, AccD at 18 months was significantly correlated with AccD at 21 months, *r* = .638, *p* = .001; and AccD at 21 months was significantly correlated with AccD at 24 months, *r* = .641, *p* = .001. However, AccD at 18 months did not correlate significantly with AccD at 24 months, *r* = .339, *p* = .155. This suggests that performance on the ECITT, as measured by AccD, is stable across a 3-month period during the second year of life, although the stability across the longer time interval between 18 and 24 months did not reach statistical significance. (A power calculation performed in G*Power version 3.1 [57] indicated that a sample size of *N* = 68 would be needed to detect an effect size of *r* = .339 with a one-tailed alpha level of .05 and 90% power).

**Table 2:** Correlations between accuracy difference (AccD) scores at 18, 21 and 24 months of age in the longitudinal sample in Study 2 (95% confidence intervals in brackets, using bootstrapping with 1000 samples).

	AccD 21 months	AccD 24 months
**AccD 18 months**	*r* = .638** (.255; .820) *p* = .001 *n* = 23	*r* = .339 (-.449; .646) *p* = .155 *n* = 19
**AccD 21 months**		*r* = .641** (.028; .859) *p* = .001 *n* = 24

** *p* < .01.

The RTD longitudinal correlations can be seen in Table 3. On the whole, the RTD was not stable across age. There was an unexpected negative correlation between RTD at 18 months and RTD at 24 months. This association indicated that toddlers with a larger RTD at 18 months had a smaller RTD at 24 months. However, the association should be interpreted with caution as it was driven largely by a single outlier at 18 months who had a large median RTD of 2025 ms (over 3 SD above the group mean). Without this outlier, the correlation dropped to *r* = -.381, *p* = .161). The RTD correlation table with this participant excluded is presented in S3 Table.

**Table 3:** Correlations between reaction time difference (RTD) scores at 18, 21 and 24 months of age in the longitudinal sample in Study 2 (95% confidence intervals in brackets, using bootstrapping with 1000 samples).

	RTD 21 months	RTD 24 months
**RTD 18 months**	*r* = .051 (-.192; .518) *p* = .836 *n* = 19	*r* = -.679** (-.888; .101) *p* = .004 *n* = 16
**RTD 21 months**		*r* = .106 (-.216; .555) *p* = .638 *n* = 22

** *p* < .01.

#### Exploratory analysis: Associations between the ECITT and other IC tasks

Participants in Study 2 also completed a broader set of executive function tasks. These tasks were part of a separate longitudinal study and the ECITT was not added to the protocol before 18 months (with a small number of children also completing the task at 15 months). As such, these EF tasks were not included as validation tasks for the ECITT and it would be statistically inadvisable to correlate all of these other tasks with the ECITT for validation purposes, especially as some of them targeted other constructs than response inhibition. The risk of spurious findings would be high with such a large number of tests in a sample of 38 children. Nevertheless, two of the tasks, a Reverse Categorisation task [58] and a Prohibition task [5], could potentially tap into similar inhibitory mechanisms, so we explored associations between the ECITT and these two tasks. The results of these analyses are presented in full in S4 File. A large proportion of toddlers had floor effects on the Reverse Categorisation task, so this correlation analysis was dropped. As regards the Prohibition task, no significant associations were found, although there were a couple of trends which would be interesting to follow up on in future work; these are discussed briefly in S4 File. Further exploratory analyses can be run on data in the Study 2 data file, which is available on OSF (https://osf.io/ytfdp/). Developmental progression and associations between the full set of infant and toddler EF measures in Study 2 is the topic of a separate report.

### Discussion

In Study 2 we investigated longitudinal development in ECITT performance between 18 and 24 months of age. As in Study 1, we found a significant effect of condition, both in terms of accuracy and RT. Toddlers made more errors on inhibitory trials and were slower to respond on correct inhibitory trials. Importantly, we also established developmental progression in performance on the task in children younger than 2 years of age. Whereas accuracy performance on the prepotent trials changed little across age (> 90% at all ages), we observed significant improvement on the inhibitory trials within the same group of children between 18 and 24 months of age. This suggests that the ECITT is sensitive specifically to the development of response inhibition, even in young toddlers. Toddlers also generally responded consistently on the inhibitory trials within each session (mean ⍺ for 8 inhibitory trials > 0.70). Finally, individual differences in accuracy performance on the task were stable between each age point, indicating that the task is promising in terms of picking up stable individual differences in inhibitory performance already during the second year of life.

In study 3, we sought to broaden the applicability of the task to older age groups. We did this partly for validation purposes, but also to demonstrate that it is possible to develop inhibitory control tasks that retain the same structure across the lifespan.

## Study 3

### Study overview and predictions

In Study 3, we used the Early Childhood Inhibitory Touchscreen Task–Adult version (ECITT-A) to further validate the task and investigate performance on the task across the lifespan. An important aim of Study 3 was to demonstrate that it is possible to create a response inhibition task that is structurally equivalent across the lifespan, and which also demonstrates the expected condition and age effects. For this reason, the ECITT-A had an identical task structure to the ECITT and differed only in superficial features that enabled a faster, and therefore more challenging, task administration for older children and adults. Several different elements of a response inhibition task can be manipulated to make the task more difficult, such as increasing the working memory load, the amount of perceptual interference, the number of ‘Go’/prepotent trials and the time pressure [18, 31, 38, 59]. We did not want to complicate the task design by increasing demands on other cognitive functions, such as working memory or perceptual interference, as it could potentially muddle what the task was measuring. That left us with the option of either increasing task speed or lowering the ratio of inhibitory to prepotent trials in the ECITT-A (i.e., relatively more prepotent trials). We opted for speed for two reasons. Firstly, we know that slower speeds (e.g., imposed delays) make the prepotency of a dominant response dissipate and therefore easier to inhibit [60–63], and that moderately increasing trial speed in a button-press Go/NoGo task increases inhibitory demand [38], presumably by increasing the prepotency of the Go-response. By making task administration faster in the ECITT-A (i.e., no animations, plus encouragement to respond as fast as possible), prepotent responses would have less time to dissipate and the task would therefore be harder and more age-appropriate for older children and adults. Secondly, from a pragmatic perspective it was preferable to increase speed instead of the number of prepotent trials relative to inhibitory trials. This is because a lower ratio of inhibitory to prepotent trials would result either in fewer inhibitory trials for analysis or the need for more trials to be administered overall, a particular concern when working with child participants, who are more limited in how many trials they can complete before disengaging with the task.

In Study 3, a sample of primary school age children, young adults and older adults performed the ECITT-A first, then a Simple Reaction Time task, followed by the Stop-signal task. It was predicted that, in line with previous research on the Stop-signal task [10, 33, 34, 36], performance on the ECITT-A, as assessed by AccD and RTD, would show a quadratic (u-shaped) relation with age. That is, we predicted peak performance in young adulthood, but lower performance in children and older adults. We also predicted that performance on the ECITT-A would be significantly correlated with performance on the Stop-signal task, even when controlling for processing speed (Simple RT) and age, consistent with inhibitory control being the shared function between the two tasks.

### Method

#### Participants

Sixty-four participants were recruited from Norfolk, Suffolk, and Essex in the United Kingdom: 27 children (9 males, 18 females; 6–9 years, *M* = 7.93 years, *SD* = 0.73), 17 younger adults (3 males, 14 females; 20–30 years, *M* = 22.88 years, *SD* = 3.37) and 20 older adults (9 males, 11 females; 58–84 years, *M* = 69.65 years, *SD* = 6.50). Children were recruited from Essex primary schools; younger adults via social networking websites; and older adults from church social groups in Norfolk and Suffolk. Further demographic details can be found in Table 1. There were no exclusion criteria, and the only inclusion criteria were participants’ availability and willingness to take part in the research. No information was collected on whether children had learning or developmental disabilities, which is a limitation of this sample, however, all children in Study 3 attended mainstream schools. The study received ethical approval from the Faculty Ethics Committee at the University of Essex (Ref. No. KH1403). Adult participants provided written informed consent. A parent or guardian provided written informed consent for child participants (the child provided verbal assent).

#### ECITT-A

*Apparatus and stimuli*. An iPad of the same dimensions as in Study 1 and 2 was used in Study 3. Most of the stimuli were also the same (any differences in stimuli between the ECITT and the ECITT-A are detailed in the following). An illustration of the stimuli and procedure used in the ECITT-A is presented in S2 Fig. In this version of the task, a red dot was displayed in the centre of the screen at all times. The dot was used to re-centre the response finger between trials. The red dot was at a 38-mm distance from each of the two blue response buttons. No animations were presented after correct responses in the ECITT-A, the stimuli simply disappeared after both correct and incorrect responses. However, after 32 experimental trials a short animation played to indicate the end of the block.

*Procedure*. Participants sat at a desk facing the iPad, which was placed on a tablet stand at a slight angle, while the experimenter stood behind them controlling the initiation of each block. First, four practice trials were presented. Participants were instructed to place their index finger on the red dot and to press the “happy face” as fast as possible before returning to the dot. The buttons were then presented on the screen. Each subsequent trial was presented automatically 1000 ms after the previous response. Trials did not ‘time out’–the two buttons remained on the screen until a response was made, as was the case in the toddler version. The smiley appeared in the prepotent location on all practice trials. After the practice, participants completed 3 blocks of 32 experimental trials (96 trials in total) with a short break between each block. In each block, the smiley appeared in the prepotent location on 24 trials and in the inhibitory location on 8 trials. Each experimental block always started with at least 3 prepotent trials. The randomisation procedure and constraints were the same as detailed for Study 1. Whether the prepotent location was at the top or bottom was manually counterbalanced across participants. After each block, the participant’s mean RT was displayed on the screen and the participant instructed: “Please try to respond faster in the next block. But also remember to respond as accurately as possible.”

*Data analysis*. ECITT-A practice trials were excluded from analysis. Incorrect responses were removed from the RT analyses, as were RTs below 200 ms or above 5000 ms. A lower cut-off for fast responses was chosen due to the faster manual responses of school-age children and adults compared to toddlers; a 200-ms cut-off is in line with other research on response inhibition in school-age children [59, 64]. Responses in the inhibitory and prepotent conditions were then aggregated to provide mean accuracy (%) and median RT (ms) measures for each participant in each condition. As in Study 1, a difference score was calculated for both accuracy (AccD) and RT (RTD) to obtain individual inhibitory control scores. Cronbach’s alpha was used to assess internal consistency.

Accuracy and RT data were initially analysed in 2 × 3 mixed ANOVAs, with Age as the between-subjects factor (child, younger adult, older adult) and Condition as the within-subjects factor (prepotent, inhibitory). Subsequently, to confirm predicted Age × Condition interactions, we ran planned pairwise comparisons on AccD and RTD to establish that (1) children had poorer inhibitory control than young adults and (2) older adults had poorer inhibitory control than young adults. (A supplementary analysis was also run to confirm, in our data set, the original age differences found by Williams et al. [34] using the Stop-signal task, see S5 File). Finally, to assess the construct validity of the newly developed ECITT-A, Pearson correlation coefficients were computed between AccD and stop-signal reaction time (SSRT) and between RTD and SSRT across the entire sample. To rule out the possibility that basic RT and age differences could account for the predicted positive correlation between the ECITT-A and the Stop-signal task, partial correlations were also run, controlling for median simple reaction time (SRT) and participant age.

#### Simple Reaction Time task (SRT)

*Apparatus and stimuli*. Stimuli for the SRT task were presented on a Cambridge Cognition touchscreen computer (screen size 11.8 inches) and came from the CANTAB cognitive assessment battery (CANTAB Research Suite 6; for further information, see http://www.cambridgecognition.com). The computer was placed on a desk stand, tilted at a slight angle in front of the participant. The computer was equipped with a response box (156 mm × 33 mm × 95 mm), featuring two 15 mm × 11 mm square buttons. The response box was positioned flat on the desk with the buttons presented vertically away from the participant for the SRT task (only the lower button was used for responding). The only stimulus presented on the screen during the task was a white square (46 mm × 46 mm) in the centre of the screen against a black background.

*Procedure*. Participants were instructed to press the button on the response box as fast as possible when the white square appeared on the screen. After each response, the next trial appeared after a variable delay, between 750 and 1500 ms, to avoid anticipatory responses. Participants completed 24 trials. The median RT was used in the analyses.

#### Stop-Signal Task (SST)

*Apparatus and stimuli*. An illustration of the Stop-signal procedure can be found in S3 Fig. Stimuli for the SST were presented using the same equipment and setup as for the SRT task, except for the response box, which was positioned horizontally to allow participants to respond using both buttons. The stimulus for the SST was a white arrow (62 mm × 55 mm), pointing either left or right, positioned inside a white central fixation circle (92 mm diameter). On each trial, the arrow appeared within the circle after a fixed 500 ms delay. Stimuli were displayed against a black background. The stop-signal was a 100 ms 300 Hz tone generated by the computer.*Procedure*. The task consisted of two parts. Standardised instructions were read aloud to the participant by the experimenter.

Part 1 (practice): Participants were instructed to press the left-hand button when they saw a left-pointing arrow and the right-hand button when they saw a right-pointing arrow. There was one block of 16 practice trials.

Part 2: Participants were instructed to continue responding as before, but if they heard a ‘beep’, which occurred at a variable delay from the presentation of the arrow (the *stop-signal delay*—SSD), they should not respond. Part 2 consisted of 5 blocks of 64 trials. Each block contained four sub-blocks of 16 trials: 12 go-trials and 4 stop-trials. Sub-blocks were not evident to the participant. All 16 trials within each sub-block were presented in a random order. The timing of the stop-signal changed throughout the test, depending on performance. Successfully inhibited responses increased the SSD by 50 ms on the subsequent stop-signal trial, whereas failure to stop decreased the SSD by 50 ms. The SSD eventually stabilised so that stopping occurred on approximately 50% of trials.

*Performance measure*. Each participant’s SSRT was calculated by subtracting their SSD (at 50% correct stopping) from their median reaction time on go-trials. The measure was based on the second half of sub-blocks to ensure that the SSRT was calculated from the point where the SSD had stabilised (Cantab Research Suite 6: Test Administration Guide, 2014, p. 290–291).

#### Data processing and statistical analysis

The same software was used for data processing and statistical analysis as in Study 1 and 2.

### Results

#### ECITT-A accuracy

**Fig 4:**
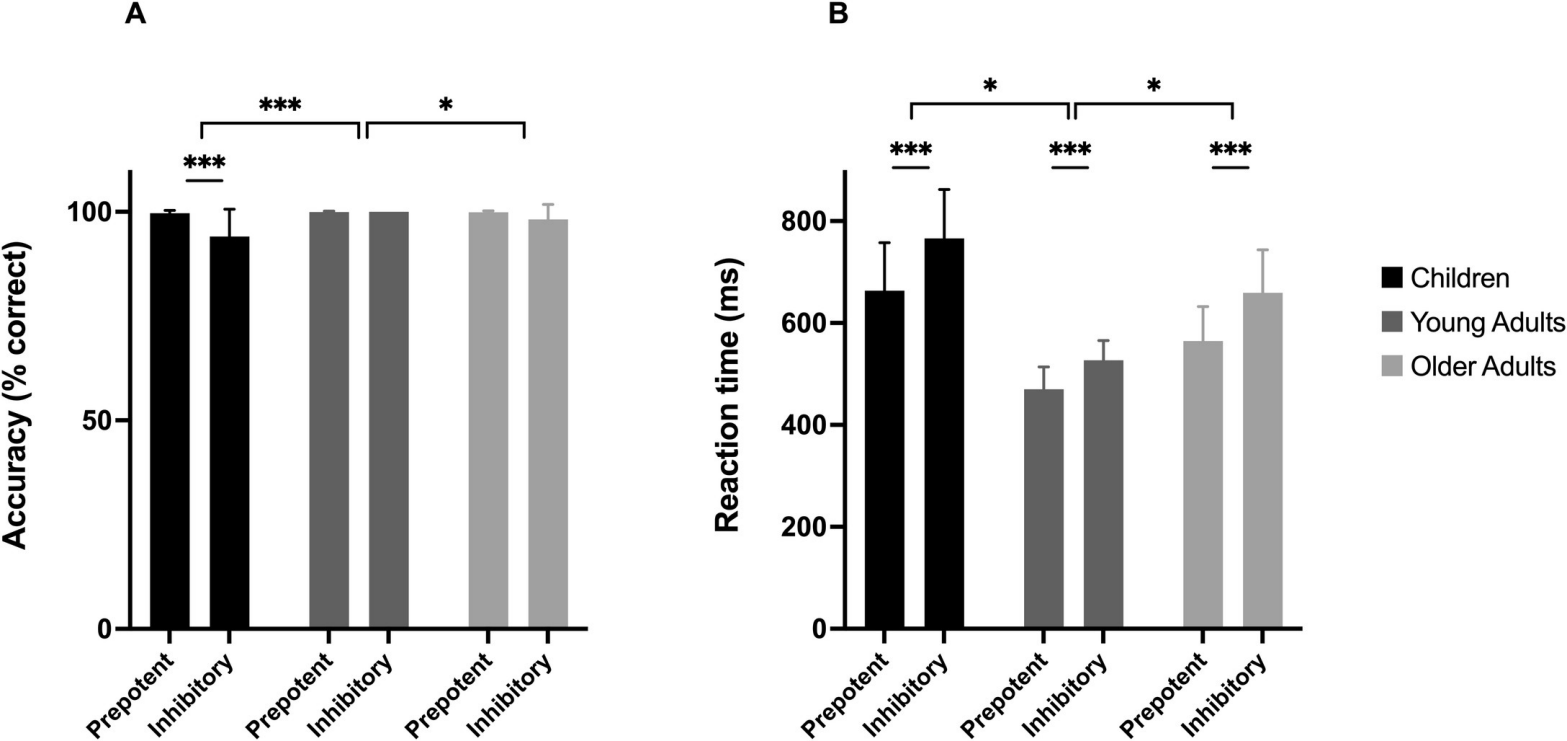
(A) Mean accuracy (%) and (B) mean median reaction time in milliseconds for inhibitory and prepotent trials in the Early Childhood Inhibitory Touchscreen Task–Adult version (ECITT-A) in Study 3. Brackets at the top indicate significant planned contrasts for the mean accuracy (AccD) and reaction time (RTD) difference scores. Error bars indicate the standard error. *** *p* < .001, * *p* < .05.

Mean accuracy scores (%) and their standard error for inhibitory and prepotent conditions of the ECITT-A are presented in Fig 4A. To analyse the data, a 2 × 3 (Condition by Age) mixed ANOVA was conducted with mean accuracy as the dependent variable. The results showed that accuracy was significantly higher in the prepotent condition (*M* = 99.81%, *SD* = 0.5%) than in the inhibitory condition (*M* = 96.94%, *SD* = 5.31%), *F*(1,61) = 15.79, *p* < .001, *η*_*p*_^*2*^ = .21. The main effect of Age was also significant, indicating that accuracy differed between age groups, *F*(2,61) = 10.14, *p* < .001, *η*_*p*_^*2*^ *=* .25. The Age × Condition interaction was statistically significant, *F*(2,61) = 8.22, *p* = .001, *η*_*p*_^*2*^ = .21, indicating that accuracy in the two trial types changed differentially as a function of age. As performance on the prepotent trials was generally very high, this suggested that the differences primarily reflected lifespan developmental changes in inhibitory control (i.e., performance on the inhibitory trials). To establish this, the interaction was followed up with the planned contrasts using the AccD score. As predicted, there was a significant difference in AccD scores between children (*M* = 5.59%, *SD* = 6.66%) and young adults (*M* = -0.06%, *SD* = 0.24%), *t*(26.11) = 4.41, *p* < .001, *d* = 1.08. There was also a significant difference in AccD scores between younger and older adults (*M* = 1.70%, *SD* = 3.50%), *t*(19.22) = 2.24, *p* = .037, *d* = 0.68, with older adults making more inhibitory (relative to prepotent) errors than younger adults. Taken together, these results suggest that the proportion of errors made in inhibitory, relative to prepotent, trials changes as a function of age. Young adults were at ceiling in both conditions, whereas children and older adults made more errors in inhibitory trials.

#### ECITT-A reaction time

Mean median RT in milliseconds (ms) and its standard error for inhibitory and prepotent trials are presented in Fig 4B. The effects of Age and Condition on median RT (henceforth, referred to as ‘RT’) were examined using a 2 × 3 mixed ANOVA. The results showed that RT was significantly faster in the prepotent condition (*M* = 581 ms, *SD* = 109 ms) than in the inhibitory condition (*M* = 669 ms, *SD* = 126 ms), *F*(1,61) = 139.19, *p* < .001, *η*_*p*_^*2*^ = .70. The main effect of Age was also significant, indicating that overall RT differed between the age groups, *F*(2,61) = 46.24, *p* < .001, *η*_*p*_^*2*^ = .60. The Age × Condition interaction was also statistically significant, *F*(2,61) = 3.65, *p* = .032, *η*_*p*_^*2*^ = .11, indicating that the differences in RT between the two conditions changed with age. The interaction was followed up with the planned contrasts between children and young adults and between young adults and older adults, using the RTD as the dependent measure. As predicted, the children had a larger RTD (*M* = 102 ms, *SD* = 66 ms) than young adults (*M* = 57 ms, *SD* = 40 ms), *t*(41.98) = 2.88, *p* = .006, *d* = 0.80. Older adults (*M* = 95 ms, *SD* = 54 ms) also had a larger RTD than young adults, *t*(34.30) = 2.44, *p* = .020, *d* = 0.79. Thus, using RT as the outcome measure, inhibitory control ability improved from childhood to young adulthood and diminished between early and late adulthood.

#### Internal consistency

Due to the low number of errors in young adults, internal consistency analyses in Study 3 focused on RT. A full overview of these results can be found in (Table 1B) in S2 File. All alpha values for inhibitory and prepotent trial RT were over 0.70, with only one value being under 0.80 (⍺ = 0.77). When the number of trials included in the analysis was reduced to 20 inhibitory trials and 68 prepotent trials, in order to include nearly all participants in the calculation of alpha (mean participant inclusion of 98.8% and 100% for inhibitory and prepotent trials, respectively), all alpha values were > .80. This indicates a high level of internal consistency for both inhibitory and prepotent trial RT.

#### Stop-signal task

A full analysis of the Stop-signal data is provided in S5 File.

#### Planned correlation analyses

Correlation analysis indicated that AccD and SSRT were positively correlated, *N* = 62, *r* = .35, 95% CI [0.15, 0.54], *p* = .005. (All 95% confidence intervals reported in this section were estimated using bootstrapping with 1000 samples.) A scatterplot showing this correlation can be found in S4 Fig. A partial correlation analysis showed that this correlation remained significant, with only a slight reduction in effect size, when controlling for Age, *r* = .32, 95% CI [0.12, 0.52], *p* = .013, and median SRT (as a proxy for processing speed), *r* = .32, 95% CI [0.09, 0.54], *p* = .011. When both Age and median SRT were entered into the analysis, the correlation between AccD and SSRT also remained significant, *r* = .31, 95% CI [0.09, 0.51], *p* = .016. This finding suggests that a longer SSRT (i.e., poorer response inhibition) is associated with more errors in inhibitory, relative to prepotent, trials on the ECITT-A, even when the participants’ age and baseline differences in RT are taken into account. The correlation between RTD and SSRT, however, was not significant, *r* = .20, 95% CI [-0.03, 0.41], *p* = .13, indicating that the association was specific to the accuracy measure.

### Discussion

Study 3 investigated the lifespan development of performance on the new ECITT-A. To further validate the new task, we investigated whether a similar pattern of increase and decrease in inhibitory performance would be seen on the ECITT-A as has previously been observed for the Stop-signal task across the lifespan. We also directly correlated performance on the ECITT-A with performance on the Stop-signal task. The results indicated that mid-primary school children did indeed have significantly poorer inhibitory control, as assessed by the ECITT-A, than young adults; and older adults also showed a decrement in inhibitory performance compared to young adults. Young adults performed at ceiling in terms of accuracy, but even in this group a significant reaction time difference was observed between prepotent and inhibitory trials, in the predicted direction (see Fig 4B). Furthermore, performance on the ECITT-A, at least in terms of accuracy, was significantly correlated with performance on the Stop-signal task, indicating that the two tasks to some extent measure the same inhibitory function. Together, these results provide further validation of the new task as an adequate measure of response inhibition, and one which can be used across the lifespan. The unique feature of the ECITT-A, compared to the Stop-signal task, is that it is structurally very similar to the toddler version of the task.

## Additional studies

A further study, Study 4, was undertaken to replicate the lifespan results from Study 3. Study 4 was carried out in a very different setting, at public engagement events, with the aim of establishing that the lifespan condition and age effects were present even under more noisy conditions. Participants under 4 years were administered the ECITT whereas participants over 4 years were administered the ECITT-A. The study sample consisted of 140 participants, ranging in age from 17 months to 71 years. The full results are reported in S6 File. In brief, the results of Study 3 were broadly replicated. The condition effect was solidly replicated across age. Furthermore, developmental progression in response inhibition, as assessed by both AccD and RTD, was found between the 4-7-year-old group and the 8-15-year-old group (the largest groups taking part in Study 4)–age groups that were not compared in Study 3. Thus, Study 4 confirmed that the ECITT-A is a suitable task for lifespan studies of inhibitory control.

We also undertook regression analyses of all cross-sectional data collected in this programme of research, i.e., the data from Studies 1, 3, 4 and the Pilot Study, to establish the association between age and inhibitory control performance, as assessed by the ECITT and ECITT-A, in a highly powered sample. The results from these analyses are reported in S7 File. In summary, for children under 4 years of age (*N* = 100, not including the longitudinal toddler participants in Study 2), a linear relationship between age and accuracy performance (AccD) accounted best for the data (*p* = .003). There was, however, no association between age and reaction time (RTD) performance (*p* = .935) in children under 4 years, consistent with Studies 1 and 2. In participants aged 4 years and older (*N* = 193), a quadratic (u-shaped) relation between age and performance (AccD and RTD) accounted best for the data (*p*s < .01). These analyses therefore confirm significant linear improvement in ECITT performance across toddlerhood and a u-shaped developmental function in ECITT-A performance across the lifespan.

## General discussion

Inhibitory control is considered a core executive function that allows us to function adaptively in everyday life. There are many types of inhibitory control, including ‘cool’ aspects, relating to overcoming strong response tendencies within both cognitive and motor domains, and ‘hot’ aspects, relating to resisting temptation. In the present report, we have focused on the development of response inhibition, the ability to overcome an over-learnt or prepotent response tendency. Although response inhibition has been extensively investigated in pre-school and school age children [e.g., 18, 20, 21, 22, 35, 37], relatively few studies exist investigating this function in children younger than 3 years of age. This is partly due to the lack of age-appropriate response inhibition tasks for toddlers. Furthermore, no existing response inhibition tasks can be used across toddlerhood and into the childhood and adult years without major modification, raising questions about the equivalence of the construct measured at different ages. We believe that using structurally similar tasks across age is important for enabling researchers to track the development of response inhibition from its beginnings in the first two years.

We therefore developed the Early Childhood Inhibitory Touchscreen Task (ECITT), a tablet-based task where participants need to respond more frequently to one location than the other, thus building up a prepotent response that needs overcoming on the rarer inhibitory trials at the other location. Since the ECITT was designed to detect the earliest response inhibition capabilities, factors which could potentially add to the complexity of the task and mask the emergence of this ability were minimised, such as language and working memory demands. All toddlers had to understand was that they needed to press the ‘happy face’, an easy rule to remember, and substantially less complex than discriminating between allowed and prohibited responses in the commonly used Go/NoGo and Stop-signal tasks, which are not appropriate for children under 3 years of age. To further validate the new task, we also adapted it for use with older children and adults (version ECITT-A). This allowed us to test whether a similar lifespan developmental pattern in response inhibition as seen for the Stop-signal task [33, 34, 36] could be detected with the new task.

In three independent studies (as well as additional studies reported in the Supporting Information), we investigated initial validity, reliability, and applicability of the new task. Our aim with these studies was primarily methodological in that we wished to demonstrate that the ECITT and ECITT-A showed the expected condition and age effects across the lifespan. This was essential to ensure that the task worked as intended, and, consequently, for it to be a useful tool for developmental research and other applications. However, given the limited research on response inhibition in toddlerhood, in our discussion of the findings, we also reflect on the developmental implications of the changes we observe.

First, we established that 24- and 30-month-olds were capable of performing the task and exhibited the expected effect of condition, i.e., made more errors and responded slower on inhibitory trials. Importantly, we also found that between 24 and 30 months of age children improved significantly in their performance on the inhibitory trials relative to the prepotent trials. Performance on prepotent trials remained high across age (> 90% correct) whereas performance on inhibitory trials increased from 74% to 92% between 24 and 30 months. This indicates that the developmental progression on the task is due to improvements in inhibitory control, rather than in task understanding or in the general cognitive processing involved in response selection and execution (i.e., when a simple repeated response needs to be carried out, as in the prepotent trials).

Second, we replicated these effects in a group of toddlers younger than 2 years of age, who were followed longitudinally at 18, 21 and 24 months of age. In this study, we again found that toddlers performed significantly worse on inhibitory than prepotent trials. Consistent with the first study, we observed a developmental progression on the task that was highly specific to the inhibitory condition, that is, young toddlers performed consistently high on the prepotent trials (> 90% correct), whereas performance on the inhibitory trials increased from 63% to 89% correct between 18 and 24 months. Because this improvement in inhibitory performance was observed longitudinally, we can be confident that the differences between ages are not due to unmeasured background variables between age groups (a limitation of cross-sectional studies). In our longitudinal sample, we were also able to establish the stability of inhibitory performance across the second half of the second year, and found that children’s performance was significantly correlated between consecutive assessment points. This is promising in terms of using the ECITT to measure individual differences in inhibitory control in toddlerhood. However, it is worth noting that, in both Study 1 and 2, only the accuracy measure (AccD) was sensitive to developmental progression. Although toddlers were consistently slower on correct inhibitory trials than on correct prepotent trials, this effect was constant across age. Furthermore, longitudinal stability was low for the RTD measure. For this reason, the ECITT AccD measure may be preferred in studies involving toddlers.

Third, we established that older children and adults show a condition effect on the adult version of the task (ECITT-A); although, we did find that young adults were generally at ceiling in terms of accuracy, as might be expected for such a simple task. Reaction time may therefore be a more appropriate measure to consider in this age group, although caution is warranted as we cannot be sure that accuracy and RT measure exactly the same inhibitory function; more research (preferably longitudinal) is needed to establish this. Despite the accuracy ceiling effect in young adults, the results of Study 3 were encouraging in terms of task validation: both accuracy and RT measures showed a similar u-shaped development of response inhibition in the ECITT-A as has been previously demonstrated with the Stop-signal task [33, 34]. Performance on the ECITT-A was also found to be significantly correlated with Stop-signal performance, even when age and simple RT were partialled out, suggesting inhibitory control as the common functional substrate. These findings, along with additional data presented in S6 and S7 Files, provide further validation of the new task as a suitable measure of response inhibition across the lifespan, although further work is needed to reduce ceiling effects in adult populations.

In addition to the longitudinal data in Study 2, which indicated significant stability in accuracy performance across 3-month intervals (this can be considered a lower bound for test-retest reliability), internal consistency was also generally acceptable to high (see S2 File). Cronbach’s alpha ranged between 0.61 and 0.86 for inhibitory trial accuracy in toddlers in Studies 1 and 2. The only exception to this was that, in the 30-month-olds in Study 2, if only 6 trials were considered (which was necessary to be able to include 100% of participants in that study), then alpha dropped to 0.44. In older children and adults in Study 3, alpha ranged between 0.77 and 0.93 for inhibitory trial RT. (Prepotent trial RT had even higher alpha values, all > 0.90). These analyses suggest that participants generally perform consistently within the same test session, with the only caveat being that at 30 months, where toddlers start to have high accuracy on the ECITT inhibitory trials, 8 inhibitory trials are needed to obtain a reliable estimate of inhibitory performance.

Taken together, these findings provide evidence that the Early Childhood Inhibitory Touchscreen task demonstrates the expected condition and age effects, which is the first step in validating the task as an adequate measure of response inhibition. Individuals of all ages were slower to respond when they had to switch their response from the prepotent to the inhibitory location. Furthermore, children and older adults made more errors specifically when the inhibitory response had to be produced, a finding consistent with the previous literature using the gold-standard Stop-signal task [33, 34]. Individual differences in performance were broadly consistent both within sessions and across time (internal consistency and longitudinal stability), although in toddlers this was only the case for accuracy performance. Importantly for the aims of the present study, significant developmental progression was observed in terms of response inhibition between 18 and 30 months of age, an age where only a few inhibitory control tasks are currently available, and those that do exist are typically too difficult for a large proportion of toddlers [39, 40, 65]. Consequently, we did not see the ‘dip’ in performance between 2 and 2½ years observed by Petersen et al. [40] in their meta-analysis of early IC tasks, and we were able to successfully assess toddlers down to 18 months of age. This suggests that the ECITT is easy to understand even for younger toddlers, providing a measure that is sensitive to inhibitory ability and relatively free from the attrition resulting from high language and memory demands.

Other research has, in line with the structural similarity principle that we argue for here, successfully adapted various IC tasks to be identical or highly consistent from 4 years of age and up to adulthood [66–68]. However, we believe that with simpler tasks that can be gradually increased in difficulty, such as the ECITT, we can study response inhibition (as well as other types of IC) successfully from a much earlier age. Such tasks will allow us to circumvent at least some of the interpretational difficulties involved in switching between different IC tasks every 1–2 years across the toddler and early childhood years [40, 47]. Being able to track IC with consistent measures across toddlerhood will be extremely useful, as it will eventually enable us to look at developmental trajectories in IC, and their outcomes in longitudinal research (using, for example, growth curve models, which require identical measures over time [69]). This includes the potential identification of maladaptive IC trajectories at an earlier point in development, which may be useful in clinical and intervention research. Such tasks will also allow us to relate IC development more precisely to other key domains during the toddler and pre-school years (e.g., social function, language). The ECITT is an initial effort to create such a structurally equivalent task, although more than one task is of course needed to gain a comprehensive picture of early IC development.

As is the case for any study, the current study had a number of limitations. Although the differences between the toddler and adult version of the ECITT were minimised as much as possible, further validation is needed. For example, longitudinal research demonstrating stability of individual differences in inhibitory performance on the task between toddlerhood (ECITT) and middle childhood (ECITT-A) would further strengthen the task’s construct validity and usefulness. Furthermore, in Study 1, the 30-month-olds performed close to ceiling level, even in the inhibitory condition, and, when assessed longitudinally, toddlers in Study 2 approached ceiling performance already at 24 months of age. This suggests that an intermediate version of the task, perhaps involving shorter animations (and thereby faster trial presentation), is needed to cover the pre-school range between approximately 2.5 and 5 years. Similarly, the ECITT-A is clearly too easy for young adults, who were at ceiling in terms of accuracy on the task. As such, although including the ECITT-A for validation purposes provided useful data, and RT results confirmed our hypotheses in terms of condition and age effects, for a true estimation of response inhibition ability in adolescents and adults, the Stop-signal task is still preferable. Lagattuta and colleagues [66] demonstrated that simple changes in stimulus features can substantially widen the applicable age range for a Stroop-like IC task. Therefore, in future, task parameters of the ECITT-A could be further adjusted. For example, in the current investigation, we adjusted time pressure to make the task harder, however, another variable that could be adjusted to increase difficulty is the proportion of prepotent trials [31, 59]–a ratio of 1 inhibitory trial to 9 prepotent trials would likely be more challenging for adults.

In addition to adjusting the task to smoothly assess response inhibition across all ages, more work is needed to establish the task’s reliability. Based on the presented evidence, we consider the group level effect of condition robust. However, as demonstrated in recent research on inhibitory control in adults [14, 15], as well as more generally in the field of infancy research [70], robust experimental effects do not necessarily translate into reliable individual differences. Such tasks with robust condition effects can in fact be detrimental to correlational research if the range of individual variation is restricted [15] or if it is smaller than the trial-level measurement error [13]. We believe, however, that the current set of studies show that there is plenty of variation in young children’s ECITT performance. Toddlers’ performance on inhibitory trials ranged from all correct to all incorrect, and the reliability analyses (internal consistency and longitudinal stability) confirmed that toddlers who make many inhibitory mistakes do so consistently within a session and across a 3-month period. This is despite the robust group-level effects and substantial developmental change. We do however acknowledge that the current report presents no evidence for test-retest reliability in the classical sense of establishing that performance is stable across a short period of time, typically 1–2 weeks. ECITT accuracy test-retest reliability has been assessed in infants, and was found to be significant but modest [71, 72]. We expect test-retest reliability to be higher in toddlers where data is generally less noisy, but no data is presently available on short-term test-retest reliability in this age group. It will also be important to establish test-retest reliability of performance on the ECITT-A in the future, as some of the issues relating to low reliability of inhibitory task performance pertains particularly to the use of RT difference scores [13, 15].

More broadly speaking, it is important to bear in mind that the ECITT was developed specifically to tap into *response inhibition* and therefore cannot be considered a universal measure of IC for toddlers. In order to truly assess the development of a complex construct such as IC, multiple tasks are needed. In particular, factor analytic work using a range of both ‘hot’ and ‘cool’ IC tasks from the early toddler years onwards will be needed to fully delineate the development of different types of IC. As such, the ECITT is just one out of many tasks needed in this type of research. On a related note, others have argued for the importance of heterotypic continuity in IC measures–the idea that different tasks *can* in fact measure the same function and that, by using structural equation modelling within a longitudinal design, the trajectory in this underlying function is trackable by careful task selection [40]. We do not argue against this idea; having a range of different tasks, and being able to establish the underlying core functions and their development, will only benefit research. Having said that, we believe that the current report provides evidence for the feasibility of developing structurally equivalent tasks across a wide age span, starting even at the youngest ages, and we hope that this is an approach that can be transferred to other task development efforts in the field.

Finally, in the analyses presented here, the youngest toddlers were 18 months old, but previous research suggests that basic response inhibition abilities emerge even earlier than this age. In fact, research has demonstrated that some inhibitory control abilities start to emerge as early as 6 months of age and continue to strengthen during the second half of the first year of life [44, 46, 73, 74]. The paradigms used to assess IC in infancy have typically relied on either visual responses or have involved components of both response inhibition and working memory (such as in the A-not-B task [44]), and none have been developed into tasks that maintain the same structure into the toddler and pre-school years. However, both in the supplemental analyses to the current report and in more recent research, we have found evidence that the ECITT can be used already in infancy. In the present study, even in the small sub-sample of longitudinal participants in Study 2 who had data available at 15 months (*N* = 11), we observed a significant effect of condition (*p* = .008, see S3 File), with participants making more errors on the inhibitory trials (54% correct), while still being correct on 89.9% of prepotent trials. Furthermore, in a recent study of 70 infants followed longitudinally at 10 and 16 months, we found a robust ECITT condition effect already from 10 months of age. Ten-month-old infants were 85.2% correct on prepotent trials in contrast to only 49.4% correct on inhibitory trials, demonstrating the substantial difficulty infants have with response inhibition. This study also indicated that, by 16 months of age, performance on the ECITT correlates significantly with performance on the A-not-B task [71]. In a separate sample of 135 infants, we used the ECITT in combination with functional near-infrared spectroscopy and found evidence that, already at 10 months, infants activate classic response inhibition areas of the brain while performing the task [72].

In conclusion, the ECITT is a novel response inhibition task that can be used from as early as 18 months of age (with recent additional evidence suggesting that it can be used even earlier) and which maintains its structure across the lifespan. It is our hope that, in combination with other tasks, it will provide a useful tool to developmental researchers and others interested in assessing inhibitory control skills in the early toddler years, an age where this domain is still relatively understudied. Due to the structural equivalence of the task at different ages, with the potential to add further levels of difficulty in future versions, the task is also ideally suited for longitudinal research where the aim is tracking individual developmental trajectories over time in order to establish the correlates and outcomes of early inhibitory control development.

## Supporting information

S1 File Pilot Study. (DOCX)

S2 File Reliability of ECITT trial accuracy and reaction time. (DOCX)

S3 File Analysis of ECITT data collected at 15 months in the longitudinal sample in Study 2. (DOCX)

S4 File ECITT performance in relation to Reverse Categorisation and Prohibition task performance in Study 2. (DOCX)

S5 File Replication of Stop-signal task age effects found by Williams et al. (1999). (DOCX)

S6 File Public engagement study (Study 4). (DOCX)

S7 File Regression analyses of pooled cross-sectional data sets (Studies 1, 3, 4 and Pilot Study). (DOCX)

S8 File ECITT demo instructions. (DOCX)

S1 Protocol ECITT testing protocol. (DOCX)

S2 Protocol ECITT coding protocol. (DOCX)

S1 Fig Stimulus parameters in the Early Childhood Inhibitory Touchscreen Task. (DOCX)

S2 Fig Illustration of the Early Childhood Inhibitory Touchscreen Task–Adult version (ECITT-A). (DOCX)

S3 Fig Illustration of the Stop-signal task procedure. (DOCX)

S4 Fig Scatterplot showing the correlation between ECITT accuracy difference (AccD) score and Stop-signal reaction time (SSRT) in children and adults in Study 3. (DOCX)

S1 Table Demographic information for participants in Studies 1, 2 and 3 (large version). (DOCX)

S2 Table Correlations between accuracy difference (AccD) scores at 18, 21 and 24 months of age in Study 2 with one participant excluded due to having a reaction time difference (RTD) score more than 3 standard deviations above the group mean at 18 months. (DOCX)

S3 Table Correlations between reaction time difference (RTD) scores at 18, 21 and 24 months of age in Study 2 with one participant excluded due to having a RTD score more than 3 standard deviations above the group mean at 18 months. (DOCX)

S4 Table Hierarchical regression analyses of age as a predictor of Early Childhood Inhibitory Touchscreen Task (ECITT) and Early Childhood Inhibitory Touchscreen Task–Adult version (ECITT-A) accuracy difference (AccD) and reaction time difference (RTD) scores in participants with age in months available across Studies 1, 3, 4 and the Pilot Study. (DOCX)
