# Supplementary material for: The early childhood inhibitory touchscreen task: A new measure of response inhibition in toddlerhood and across the lifespan
Source: PLoS One. 2021 Dec 2;16(12):e0260695. doi: 10.1371/journal.pone.0260695 (PMC8638877; doi:10.1371/journal.pone.0260695)
Supplement: S1 File — (DOCX) [file pone.0260695.s001.docx]

S1 Supporting Information: Pilot Study

Study overview and predictions

In the pilot study, a small group of toddlers, ranging in age from 20 to 28 months, performed the Early Childhood Inhibitory Touchscreen Task (ECITT) to establish that the expected condition effect was present. We predicted that (1) toddlers would make more errors on inhibitory trials compared to prepotent trials, and (2) would respond slower on correct inhibitory trials compared to correct prepotent trials.

Method

*Participants*

Parents who had signed up to the University of Essex Babylab volunteer database were contacted if they had a child around 2 years of age (for the initial pilot study, the age range was allowed to be wide). If parents were happy to take part, a visit to the lab was scheduled. Fifteen toddlers, 10 girls and 5 boys, were recruited this way. Toddlers ranged in age from 20 to 28 months (*M* = 25.12 months, *SD* = 2.34). Participants were from the Colchester area in Essex, United Kingdom. Demographics of the sample can be found in Table 1 below. The study received ethical approval from the Faculty Ethics Committee at the University of Essex (Ref. No. KH1403).

Apparatus, stimuli and procedure

Toddlers first completed a short warm-up game to familiarise them with the touchscreen (same as in Study 1, but not presented as a task, simply a game). Stimuli were presented on an Apple iPad tablet, with a screen size of 9.7 inches and a resolution of 1024 × 768 pixels. In contrast to Study 1, each ECITT trial was presented individually by a second experimenter (using the controller device). The iPad was placed in a desk stand, tilted at a slight angle in front of the participant. In the pilot study, the screen was not moved out of reach between trials. Other than that, the stimuli and procedure were the same as in Study 1.

Data analysis

Video coding, data cleaning and calculation of accuracy and RT measures followed the same procedures as detailed in Study 1. In the Pilot Study, 2 toddlers were less than 60% correct on prepotent trials and were therefore excluded from the analyses. Accuracy and RT measures were analysed using planned paired-samples t tests.

Results

ECITT Accuracy

On average, toddlers were 95% correct on prepotent trials (*SD* = 7%) and 81% correct on inhibitory trials (*SD* = 19%). A paired-samples t test indicated that the difference between conditions was statistically significant in the predicted direction, *t*(12) = 2.99, *p =* .011, *d* = .83.

ECITT Reaction Time

The mean median reaction time (RT) on correct prepotent trials was 1506 ms (*SD* = 319 ms), and the mean median RT on correct inhibitory trials was 1751 ms (*SD* = 625 ms). A paired-samples t test indicated that the difference between conditions was approaching significance in the predicted direction, *t*(12) = 2.13, *p* = .055, *d* = .59.

Discussion

In an initial pilot sample of 15 children aged 20 to 28 months we established that, as predicted, toddlers made significantly more errors on inhibitory trials than on prepotent trials. There was a trend in the same direction for RT, with toddlers taking approx. 250 ms longer to respond on inhibitory trials. These findings provided preliminary evidence that the ECITT is suitable for assessing response inhibition in toddlers, at least when accuracy is used as the dependent measure.

|  | *Mean* or % |
| --- | --- |
| **Child Characteristics:** |  |
| *N* | 15 |
| Age (months) | 24.79 |
| Sex |  |
| % Female | 66.67% (10/15) |
| Ethnicity |  |
| White-British | 60.00% (9/15) |
| Mixed - White and Asian | 0.00% (0/15) |
| Afro-Caribbean | 0.00% (0/15) |
| White - British/Irish | 0.00% (0/15) |
| Other White Background | 0.00% (0/15) |
| Other Mixed Background | 13.33% (2/15) |
| Other Black Background | 0.00% (0/15) |
| Mixed - White and Black African | 0.00% (0/15) |
| Not provided | 26.67% (4/15) |
| **Maternal Characteristics:** |  |
| Age (years) | 37.00 (12/15) |
| Total years in education | 15.82 (12/15) |
|  |  |

**Table 1.** Demographic data for participants in the Pilot Study.
