# Supplementary material for: The early childhood inhibitory touchscreen task: A new measure of response inhibition in toddlerhood and across the lifespan
Source: PLoS One. 2021 Dec 2;16(12):e0260695. doi: 10.1371/journal.pone.0260695 (PMC8638877; doi:10.1371/journal.pone.0260695)
Supplement: S2 File — (DOCX) [file pone.0260695.s002.docx]

**S2 Supporting Information: Reliability of ECITT trial accuracy and reaction time**

**Internal consistency analyses**

Whereas measurement reliability is usually reported for questionnaire measures, it is much less commonly reported for experimental tasks ([1](#_ENREF_1)). Nevertheless, it is recommended that measurement reliability is reported for experimental tasks ([2](#_ENREF_2)), including in developmental research ([3](#_ENREF_3)). Not only is measurement reliability important for establishing that individual differences can be reliably measured with the task, reliability also constrains the associations that can be detected between the task in question and other tasks. Very large samples are needed to establish a true correlation between two tasks with low measurement reliability ([3](#_ENREF_3), [4](#_ENREF_4)).

Whereas test-retest reliability assesses the consistency of individual differences across two separate sessions (usually spaced about a week apart), internal consistency assesses the consistency of individual differences within a single session. (Test-retest at longer time intervals, e.g., several months or even years, becomes an estimate of longitudinal stability). Two methods for assessing internal consistency are often used, the Spearman-Brown split-half coefficient and Cronbach’s alpha. In most cases, Cronbach’s alpha is preferred because it assesses the mean of all possible split halves ([5](#_ENREF_5)).

Table 1a presents Cronbach’s alpha values for inhibitory trial accuracy in Study 1 (24 and 30 months, cross-sectional) and Study 2 (18, 21 and 24 months, longitudinal). We focussed on inhibitory trial accuracy for these analyses because performance on these trials is most likely to tap into the core function that we were interested in, namely, response inhibition. Whereas the consistency of a difference score would have been useful, there is no accurate way of determining the internal consistency of a difference score because Cronbach’s alpha is based on performance on each individual trial in the session and trials cannot be ‘combined’ for this analysis. We also did not analyse toddler inhibitory reaction times (RTs) because some toddlers responded incorrectly on most inhibitory trials, meaning that only a small subset (toddlers who performed well on inhibitory trials) would have been included in the internal consistency analysis for RT (for a discussion of this issue, see Green et al. ([2](#_ENREF_2))).

Cronbach’s alpha can only be calculated for participants who have all trials available. For Studies 1 and 2 this means that participants would have to have 8 valid inhibitory trials available to be included. For this reason, Table 1a presents Cronbach’s alpha values both for participants who have a score (correct or incorrect) for all inhibitory trials (8 in total) and for participants who have at least 6 inhibitory trials available for analysis. Whereas the second column contains alpha values based on 8 trials (but fewer participants are contributing to those values), the third column contains alpha values based on most of the participants, but this estimate is based on fewer trials (6 trials).

**Table 1a.** Reliability for ECITT inhibitory trial accuracy in Studies 1 and 2.

|  | **8 trials** | | **6 trials** | |
| --- | --- | --- | --- | --- |
|  | *n included^a^/total* | *Cronbach's ⍺* | *n included^a^/total* | *Cronbach's ⍺* |
| **Study 1 (cross-sectional)** |  |  |  |  |
| 24-month-olds | 29/35 (82.9%) | 0.859 | 35/35 (100%) | 0.774 |
| 30-month-olds | 39/42 (92.9%) | 0.745 | 42/42 (100%) | 0.437^b^ |
| *Mean for Study 1* | *87.9%* | *0.802* | *100.0%* | *0.606* |
| **Study 2 (longitudinal)** |  |  |  |  |
| 18-month-olds | 15/25 (60.0%) | 0.800 | 23/25 (92.0%) | 0.665 |
| 21-month-olds | 24/28 (85.7%) | 0.796 | 27/28 (96.4%) | 0.636 |
| 24-month-olds | 25/29 (86.2%) | 0.663 | 29/29 (100%) | 0.810 |
| *Mean for Study 2* | *77.3%* | *0.753* | *96.1%* | *0.704* |
| ***Grand mean*** | ***82.6%*** | ***0.778*** | ***98.1%*** | ***0.655*** |

*Note.* As in the main analyses (see Method of Study 1), participants who were less than 60% correct on prepotent trials were excluded. ^a^ Cronbach's alpha (⍺) can only be calculated for participants with all trials (8 or 6, depending on column) available. ^b^ With 7 trials available (all 30-month-old participants had at least 7 trials available), Cronbach’s alpha increased to .609.

In general, a Cronbach’s alpha value over 0.70 is considered sufficiently reliable ([5](#_ENREF_5)). As can be seen from Table 1a, alphas were mostly acceptable for participants who had 8 trials available (mean alpha across assessments = 0.778). However, when the assessment was based on only 6 trials, several alphas dropped below 0.70. Most of these values stayed above 0.60, but Cronbach’s alpha at 30 months (Study 1) dropped down to only 0.437 when based on 6 trials (0.609 when based on 7 trials, as per the table note). This is likely due to toddlers starting to show ceiling effects in performance on the ECITT at around 30 months (see the General Discussion for a discussion of ceiling effects). Ceiling performance results in low between-subjects variability, and, as demonstrated in recent research with both adults and infants, low between-subjects variability has the effect of lowering reliability ([3](#_ENREF_3), [4](#_ENREF_4)). Nevertheless, it is encouraging that at least when 8 trials are available (which is the case for 92.9% of the 30-month-old group), there appears to be enough between-subjects variability in the data to obtain a reliable within-session measure of performance. Taken together, the internal consistency analyses of Study 1 and 2 suggest that experimenters should aim to obtain at least 8 inhibitory trials from toddlers to ensure reliable measurement of inhibitory performance, especially at 30 months.

Table 1b presents internal consistency analyses for the Study 3 ECITT-A data from school age children, adults and older adults. For these analyses, we looked at RT because young adults were at ceiling in terms of accuracy (meaning no individual differences, and for this reason Cronbach’s alpha could not be calculated). RT therefore constituted a more meaningful measure in terms of assessing internal consistency across all age groups in Study 3. Again, it is not possible to investigate a difference score because the analysis is done at the trial level within a single session. We therefore present analyses of both inhibitory trial RT and prepotent trial RT. As for the data from Study 1 and 2, Table 1b presents Cronbach’s alpha for participants with all trials available (24 inhibitory and 72 prepotent trials, second column) and for participants with a smaller number of trials available (20 inhibitory and 68 prepotent trials, third column).

| **Inhibitory trial RT** | **24 trials** | | **20 trials** | |
| --- | --- | --- | --- | --- |
|  | *n included^a^/total* | *Cronbach's alpha* | *n included^a^/total* | *Cronbach's alpha* |
| Children | 9/27 (33.3%) | 0.771 | 26/27 (96.3%) | 0.887 |
| Young adults | 17/17 (100.0%) | 0.933 | 17/17 (100.0%) | 0.915 |
| Older adults | 14/20 (70.0%) | 0.876 | 20/20 (100.0%) | 0.814 |
| *Mean (all age groups)* | *67.7%* | *0.860* | *98.8%* | *0.872* |
| **Prepotent trial RT** | **72 trials** | | **68 trials** | |
| Children | 16/27 (59.3%) | 0.958 | 27/27 (100.0%) | 0.960 |
| Young adults | 17/17 (100.0%) | 0.954 | 17/17 (100.0%) | 0.951 |
| Older adults | 15/20 (75.0%) | 0.965 | 20/20 (100.0%) | 0.957 |
| *Mean (all age groups)* | *78.1%* | *0.959* | *100.0%* | *0.956* |
| ***Grand mean*** | ***72.9%*** | ***0.910*** | ***99.4%*** | ***0.914*** |

**Table 1b.** Reliability for inhibitory trial RT and prepotent trial RT in Study 3.

*Table note.* As in the main analyses (see Method of Study 3), only correct responses with a reaction time (RT) over 200 ms and under 5000 ms were included in the analyses. ^a^ Cronbach's alpha (⍺) can only be calculated for participants with all trials (8 or 6, depending on column) available.

As can be seen from Table 1b, Cronbach’s alpha was above 0.70 for RT in both trial types and at all ages in Study 3. This indicates that individual differences in RT on both inhibitory and prepotent trials in children, young adults and older adults were highly consistent within the same session.

**References**

1. Enkavi AZ, Eisenberg IW, Bissett PG, Mazza GL, MacKinnon DP, Marsch LA, et al. Large-scale analysis of test–retest reliabilities of self-regulation measures. Proc Natl Acad Sci U S A. 2019;116(12):5472-7.

2. Green SB, Yang Y, Alt M, Brinkley S, Gray S, Hogan T, et al. Use of internal consistency coefficients for estimating reliability of experimental task scores. Psychon Bull Rev. 2016;23(3):750-63.

3. Byers-Heinlein K, Bergmann C, Savalei V. Six solutions for more reliable infant research. PsyArxiv [Internet]. 2021 April 8. Available from: <https://psyarxiv.com/u37fy/>.

4. Hedge C, Powell G, Sumner P. The reliability paradox: Why robust cognitive tasks do not produce reliable individual differences. Behav Res Methods. 2018;50(3):1166-86.

5. Johnson A. Reliability, Cronbach’s alpha. 2017. In: The SAGE Encyclopedia of Communication Research Methods [Internet]. [1415-7].
