# Supplementary material for: The early childhood inhibitory touchscreen task: A new measure of response inhibition in toddlerhood and across the lifespan
Source: PLoS One. 2021 Dec 2;16(12):e0260695. doi: 10.1371/journal.pone.0260695 (PMC8638877; doi:10.1371/journal.pone.0260695)
Supplement: S3 File — (DOCX) [file pone.0260695.s003.docx]

**S3 Supporting Information: Analysis of ECITT data collected at 15 months in the longitudinal sample in Study 2**

A subset of 11 of participants in the longitudinal sample reported on in Study 2 were also administered the ECITT at 15 months. The reason that most of the participants were not administered the task at this age was that it was introduced into the protocol toward the end of the 15-month data collection wave. The amount of data was too small to include in the repeated-measures ANOVA, and we therefore report the data separately here. As this was the first time these 11 participants completed the ECITT, the data can be considered cross-sectional.

10 out of the 11 participants tested at 15 months met the criterion of being correct on at least 60% of the prepotent trials (see Study 1 Method). The participant who did not meet this criterion was excluded from the analysis. Children were on average 15.33 months old (*SD* = 0.30).

A paired-samples t test of the proportion of correct responses indicated that 15-month-olds made significantly more errors on the inhibitory trials (54.4% correct) compared to the prepotent trials (89.9% correct), *t*(9) = 3.41, *p* = .008, *d* = 1.08. The difference between correct inhibitory and correct prepotent trials in terms of median RT was not significant, *t*(6) = -2.22, *p* = .068, *d* = 0.84 (*N* = 7 for this analysis, as 3 participants did not have any correct inhibitory trials or RTs that met the inclusion criteria).
