# Supplementary material for: The early childhood inhibitory touchscreen task: A new measure of response inhibition in toddlerhood and across the lifespan
Source: PLoS One. 2021 Dec 2;16(12):e0260695. doi: 10.1371/journal.pone.0260695 (PMC8638877; doi:10.1371/journal.pone.0260695)
Supplement: S4 File — (DOCX) [file pone.0260695.s004.docx]

**S4 Supporting Information: ECITT performance in relation to Reverse Categorisation and Prohibition task performance in Study 2**

Participants in Study 2 were taking part in a longitudinal study of executive functions across infancy and toddlerhood (for further details about this study, see the Method section of Study 2). The toddlerhood tasks were (in administration order): Looking A-not-B with invisible displacement ([1](#_ENREF_1)), Reverse Categorisation ([2](#_ENREF_2)), Shape Stroop ([3](#_ENREF_3)), 3 Boxes at 18 months ([1](#_ENREF_1)) / 6 Boxes at 21 and 24 months ([4](#_ENREF_4)), ECITT, and Prohibition task ([5](#_ENREF_5)). The aim of the broader study was to start assessing toddlers as early as possible on each task, so that development in the skills tapped by the tasks could be investigated as they emerge. The executive function tasks that toddlers completed at 15, 18, 21 and 24 months were *not* selected with task validation of the ECITT in mind; in fact, the ECITT was not added to the protocol before towards the end of the 15-month data collection wave. Correlating the ECITT with all of these EF tasks would not be statistically appropriate because the large number of correlational tests could easily result in spurious associations (Type 1 errors). Furthermore, some of the tasks tapped other EF constructs, for example the 3 Boxes / 6 Boxes task is considered a working memory task.

Nevertheless, two of the tasks administered to the Study 2 sample are thought to measure inhibitory skills, and although not traditionally considered response inhibition tasks, these tasks could potentially measure inhibitory components that are overlapping with the construct that the ECITT was developed to measure, i.e., response inhibition. We therefore considered them in a set of exploratory analyses.

The first task of interest was the Prohibition task ([5](#_ENREF_5)). The full administration protocol for the Prohibition task can be found at the end of this document. In brief, in this task toddlers are presented with an attractive toy (a glittery wand), but then told not to touch it. The longer the child can wait before touching the wand (up to 30 s), the better their Prohibition score (the latency to touch). Although this task is generally considered a hot IC task, it does involve an element of response inhibition (the child needs to inhibit the impulsive urge to grab the wand). The second task was a version of the Reverse Categorisation task ([2](#_ENREF_2)). However, preliminary analyses of this task indicated that most toddlers were at floor in the baseline condition (not involving IC), even at 24 months. The floor effect meant that we would have max. 11 participants available for the correlation analysis. Therefore, we did not analyse associations between the Reverse Categorisation task and the ECITT.

Exploratory analyses of the association between the ECITT and the Prohibition task at 18, 21 and 24 months indicated no significant associations between the two tasks at these ages, although there was a trend towards a negative correlation between ECITT AccD and Prohibition latency at 24 months, *N* = 29, *r* = -.35, 95% CI [-0.64, 0.04], *p* = .065, and a positive correlation between ECITT RTD and Prohibition latency at 18 months, *N* = 22, *r* = .38, 95% CI [0.04, 0.60], *p* = .079. These potential associations are somewhat contradictory, with the first association suggesting that better ECITT accuracy performance (smaller AccD) is associated with better Prohibition performance at 24 months, and the second association suggesting that better RT performance at 18 months (smaller RTD) is associated with poorer Prohibition performance at 18 months. A recent study in infants found no significant association between ECITT and Prohibition performance in 10- and 16-month-old infants ([6](#_ENREF_6)). We therefore suggest that more research is needed in a larger sample of toddlers before firm conclusions about the associations between these two tasks can be drawn.

**Prohibition task procedure**

The task was based on the “Glitter Wand” task first presented by Friedman et al. ([5](#_ENREF_5)). The child was seated in a highchair pulled up to a table facing the seated experimenter. The parent was seated beside and slightly behind the child and instructed to not respond during the task. The experimenter showed the child a glitter wand (a clear wand filled with clear liquid and glitter, 12” long, ½” diameter), rotating the wand to show movement of the liquid and glitter. While manipulating the wand, the experimenter talked about how pretty and fun it looked while making sure the child was looking at the wand. The experimenter told the child that they were now going to put the wand on the table and not to touch it. The experimenter then placed the wand on the table within easy reach of the child, made eye contact with the child, and said “Now, (child’s name), don’t touch”. The “don’t touch” was combined with the experimenter making prohibition hand movements (i.e., hands flat, palms down, crossing one hand over the other). The experimenter subsequently turned her body away (approx. 90-degree angle) and made no eye contact with the child. The experimenter ended the task by saying “It’s OK, you can touch it now” after either 30 s of no touching or light touches by the child or after the child picked up the wand. The measure of interest was the latency to first touch after the experimenter’s prohibition (0-30 s).

**References**

1. Diamond A, Prevor MB, Callender G, Druin DP. Prefrontal cortex cognitive deficits in children treated early and continuously for PKU. Monogr Soc Res Child Dev. 1997;62(4).

2. Carlson SM, Mandell DJ, Williams L. Executive function and theory of mind: Stability and prediction from ages 2 to 3. Dev Psychol. 2004;40(6):1105-22.

3. Kochanska G, Murray K, Jacques TY, Koenig AL, Vandegeest KA. Inhibitory control in young children and its role in emerging internalization. Child Dev. 1996;67(2):490-507.

4. Hughes C, Ensor R. Executive function and theory of mind: Predictive relations from ages 2 to 4. Dev Psychol. 2007;43(6):1447-59.

5. Friedman NP, Miyake A, Robinson JL, Hewitt JK. Developmental trajectories in toddlers’ self-restraint predict individual differences in executive functions 14 years later: A behavioral genetic analysis. Dev Psychol. 2011;47(5):1410-30.

6. Hendry A, Greenhalgh I, Bailey R, Fiske A, Dvergsdal H, Holmboe K. Development of directed global inhibition, competitive inhibition and behavioural inhibition during the transition between infancy and toddlerhood. PsyArXiv [Internet]. 2021 March 30. Available from: <https://psyarxiv.com/mhkaj/>.
