# Supplementary material for: The early childhood inhibitory touchscreen task: A new measure of response inhibition in toddlerhood and across the lifespan
Source: PLoS One. 2021 Dec 2;16(12):e0260695. doi: 10.1371/journal.pone.0260695 (PMC8638877; doi:10.1371/journal.pone.0260695)
Supplement: S5 File — (DOCX) [file pone.0260695.s005.docx]

S5 Supporting Information: Replication of Stop-signal task age effects found by Williams et al. (1999)

Analyses were run to confirm, in our data set, the original age differences found by Williams, Ponesse, Schachar and Tannock ([1](#_ENREF_1)) using the Stop-signal task. Mean Stop-signal reaction time (SSRT) in children, young adults and older adults in Study 3 is illustrated in Figure 1 below. A one-way ANOVA, with Age as the between-subjects factor and SSRT as the dependent variable, showed a significant effect of Age on SSRT, *F*(2,59) = 10.55, *p* < .001, *η_p_^2^* = 0.26. Planned comparisons were performed, using Welch’s independent-samples t test, to examine the differences in SSRT between age groups. These showed a significant difference in mean SSRT between children (*M* = 259 ms, *SD* = 90 ms) and young adults (*M* = 160 ms, *SD* = 48 ms), with children being approximately 100 ms slower at stopping than young adults, *t*(39.72) = 4.70, *p* < .001, *d* = 1.30. Additionally, as expected, there was a significant difference in SSRT between young adults and older adults (*M* = 200 ms, *SD* = 57 ms), *t*(33.88) = 2.30, *p* = .028, *d* = 0.76, with older adults being approximately 40 ms slower at stopping than young adults. In accordance with Williams et al. ([1](#_ENREF_1)), these results indicate that the ability to inhibit prepotent responses improves between childhood and young adulthood and then diminishes slightly in older adulthood.

***

*

**Figure 1.** Mean Stop-signal reaction time (SSRT) in milliseconds (ms) in children (*N* = 26), young adults (*N* = 17) and older adults (*N* = 19). Error bars indicate the standard error. Brackets at the top indicate planned contrasts. *** p < .001, * p < .05.

Reference

1. Williams BR, Ponesse JS, Schachar RJ, Logan GD, Tannock R. Development of inhibitory control across the life span. Dev Psychol. 1999;35(1):205-13.
