# Supplementary material for: The early childhood inhibitory touchscreen task: A new measure of response inhibition in toddlerhood and across the lifespan
Source: PLoS One. 2021 Dec 2;16(12):e0260695. doi: 10.1371/journal.pone.0260695 (PMC8638877; doi:10.1371/journal.pone.0260695)
Supplement: S6 File — (DOCX) [file pone.0260695.s006.docx]

S6 Supporting Information: Public engagement study (Study 4)

Study overview and predictions

Study 4 was mainly a replication study, which took place in a very different setting compared to Studies 1-3: at public engagement events. As such, data in Study 4 was collected in a much busier and noisier setting and the sample was largely an opportunity sample (e.g., museum visitors). The study received ethical approval from the University of Oxford Medical Sciences Interdivisional Research Ethics Committee (Ref. No. R50563/RE005). The aims of Study 4 were to:

1. Replicate the ECITT and ECITT-A condition effect in children and adults, i.e., participants were predicted to make more errors on inhibitory trials and respond slower on correct inhibitory trials.
2. Broadly replicate and extend the developmental effects observed in Studies 1-3: We expected (1) children under the age of 4 years to make significantly more errors and respond significantly slower on (correct) inhibitory trials than children aged 4-7, (2) children aged 4-7 to have significantly worse response inhibition than older children and adolescents (aged 8-15), and (3) middle aged to older adults to have significantly worse response inhibition than young adults. (We had no specific prediction about a difference between older children/adolescents and adults, as we had no prior evidence as to when during the teenage years ECITT-A performance reaches adult levels).

It should be noted that due to the nature of testing at public events, we were not able to match participants to the exact ages tested in Studies 1-3. In particular, the youngest age group and adults were under-represented. Nevertheless, broad confirmation of the effects of condition and age on ECITT/ECITT-A performance in a more natural and noisy environment would provide further evidence for the validity and applicability of the task across a range of settings.

Method

Participants

140 participants were recruited at public engagement events in Oxfordshire, UK. Three of the events took place at a public museum in central Oxford, one of the events was an indoor public event in Banbury (Oxfordshire), and one event took place outdoors at a street festival in central Oxford. Given the context – people briefly stopping by the stall to try the task – limited demographic information was collected in this study. Furthermore, at the first two events, we did not have ethical approval to collect any date of birth information, so only age in years was collected (*N* = 33) except for two toddlers under 2 years where age was collected in months. At the last three events, we were permitted to collect month and year of birth, and from this information, age in months was automatically calculated by the ECITT app. Participant sex was collected at two of the events (one of the museum events and at the street festival). Of these participants (*N* = 81, 58% of the total public engagement sample), 46 (57%) were male and 35 (43%) were female.

Participants were divided into the following age groups: Under 4s (*N* = 11, 17 – 45 months, *M* = 33.00 months (2.75 years), *SD* = 9.83), 4 – 7 years (*N* = 54, *M* = 71.63 months (5.97 years), *SD* = 12.91), 8 – 15 years (*N* = 53, *M* = 122.15 months (10.18 years), *SD* = 21.77), young adults (*N* = 11, 26 – 38 years, *M* = 30.39 years, *SD* = 3.43), and middle aged and older adults (40 – 71 years, *N* = 11, *M* = 58.68 years, *SD* = 11.77). The under 4s were administered the toddler version of the task (ECITT), whereas all participants aged 4 years or older were administered the adult version of the task (ECITT-A). Of note, the middle aged and older adults group in Study 4 was substantially younger than the older adults group in Study 3. Since any grouping of age can affect and potentially misrepresent results, we also ran regression analyses of the combined samples from all cross-sectional studies reported in this paper and associated supporting information (*N* = 300), including analyses of all under 4s separately from older children and adults (see S7 Supporting Information). The regression analyses used age as a continuous variable, which avoids the issue with categorising the age variable.

Apparatus and stimuli

The iPad was identical to the one used in Study 1 (an Apple iPad Air 2). The ECITT was identical to the version used in Study 1 and the ECITT-A was identical to the version used in Study 3. A single experimenter administered the task. The experimenter held the response iPad in their hand using a hand strap with the screen facing the participant (rather than placing it on a stand).

Procedure

Under 4s were administered the ECITT using the same procedure as described in Study 1. One or two practice trials (with only a single blue button in the center of the screen) were administered before 32 test trials were presented, to ensure that children understood the task before taking part. All participants aged 4 years and above were administered the ECITT-A using the same procedure as in Study 3. Participants completed 4 practice trials (2 locations, the smiley only appearing in the prepotent location) followed by a single block of 32 test trials. Only 32 trials were administered because of time constraints. Given the setting, test sessions were not recorded on video; instead the raw response data collected by the app was used in the analyses.

Data analysis

Accuracy and reaction time (RT) analyses followed the same format as those conducted for Studies 1-3. Practice trials were excluded from the analysis. Incorrect responses were removed from the RT analyses, as were RTs below 200 ms for children and adults (below 300 ms for children aged less than 4 years), or above 5000 ms.

In order to investigate the development of response inhibition as assessed by the ECITT/ECITT-A across the full age spectrum, data from all groups were analysed together. As children under 4 years completed the easier toddler version of the task, this could potentially reduce age differences between the under 4s and the 4- to 7-year-olds, however, this was deemed more appropriate than administering the ECITT-A to toddlers and preschoolers, who often do not respond well to being put under time pressure, and because a key point of the development of the ECITT was to adjust the task without changing its fundamental structure. Despite the ECITT being easier than the ECITT-A, we expected the under 4s to struggle most with the inhibitory demand and therefore perform relatively worse on inhibitory trials compared to older children and adults.

Accuracy and RT measures were analysed using 2 × 5 Age (*Under 4s, 4 – 7 years, 8 – 15 years, young adult, middle aged and older adult)* by Condition (*inhibitory, prepotent*) mixed ANOVAs with mean accuracy (%) and median RT as the dependent variables, respectively. As in Studies 1-3, AccD and RTD scores were calculated and planned contrasts (Welch’s t test) carried out to confirm the predicted developmental effects.

Results

Accuracy

Mean and standard errors for accuracy (%) in inhibitory and prepotent trials of ECITT/ECITT-A at each age are presented in Figure 1 below. A 2 × 5 mixed ANOVA was conducted with mean accuracy as the dependent variable. The results showed that accuracy was significantly higher in the prepotent condition (*M* = 99.60%, *SD* = 1.53) than in the inhibitory condition (*M* = 93.78%, *SD* = 12.16), *F* (1, 135) = 26.18, *p* < .001, *η_p_^2^* = 0.16. The main effect of Age was also significant, indicating that overall accuracy differed between age groups, *F* (4, 135) = 5.79, *p* < .001, *η_p_^2^* = 0.15. This was qualified by a significant Age × Condition interaction, indicating that accuracy in the two conditions changed differentially as a function of age, *F* (4, 135) = 4.99, *p* = .001, *η_p_^2^* = 0.13. The planned contrasts confirmed that 4- to 7-year-olds (*M* = 7.91%, *SD* = 11.10) had significantly larger accuracy difference scores (AccD) than 8- to 15-year-olds (*M* = 3.03%, *SD* = 6.94), *t*(89.26) = 2.74, *p* = .008, *d* = 0.53. However, against our prediction, although the AccD was nominally larger in under 4s (*M* = 17.13%, *SD* = 26.55) than in 4- to 7-year-olds (*M* = 7.91%, *SD* = 11.10), this difference was not statistically significant, *t*(10.72) = 1.13, *p* = .283, *d* = 0.63. The difference between young adults (*M* = 2.27%, *SD* = 5.06) and middle aged and older adults (*M* = 1.14%, *SD* = 3.77) was also not significant, *t*(18.49) = 0.60, *p* = .557, *d* = 0.26. An exploratory analysis indicated that there was also no difference in AccD between the oldest children and young adults, *t*(18.81) = 0.42, *p* = .679, *d* = 0.11.

**Figure 1.** Mean accuracy (%) for inhibitory and prepotent trials in the Early Childhood Inhibitory Touchscreen Task (ECITT) and the Early Childhood Inhibitory Touchscreen Task – Adult version (ECITT-A) in Study 4. The dashed line indicates a change to a more difficult task version (ECITT to the left of the line, ECITT-A to the right of the line). The bracket at the top indicates a significant planned contrast for the mean accuracy difference (AccD) score. Error bars indicate the standard error. *** *p* < .001, ** *p* < .01, * *p* < .05.

Reaction time

Mean median RTs (ms) and their standard error for inhibitory and prepotent trials are presented in Figure 2. The effects of Age and Condition on RT were examined using a 2 × 5 mixed ANOVA. The main effect of Condition was significant, with RT being significantly faster in the prepotent condition (*M* = 794 ms, *SD* = 392) than in the inhibitory condition (*M* = 912 ms, *SD* = 514), *F*(1, 135) = 46.32, *p* < .001, *η_p_^2^* = .26. The main effect of Age was also significant, indicating that overall RT differed between the age groups, *F*(4, 135) = 104.31, *p* < .001, *η_p_^2^* = .76. The Age × Condition interaction was statistically significant, *F*(4, 135) = 7.52, *p* < .001, *η_p_^2^* = .18, indicating that the differences in RT between the two conditions changed with age. Planned contrasts using the RTD as the dependent variable confirmed that that 4- to 7-year-olds had significantly larger RTD (*M* = 132 ms, *SD* = 182) than 8- to 15-year-olds (*M* = 71 ms, *SD* = 61), *t*(65.10) = 2.32, *p* = .023, *d* = 0.45. However, against our predictions, there was no significant difference in RTD between under 4s (*M* = 398 ms, *SD* = 530) and 4- to 7-year-olds, *t*(10.49) = 1.65, *p* = .129, *d* = 0.99, or between young adults (*M* = 49 ms, *SD* = 37) and middle aged and older adults (*M* = 66 ms, *SD* = 64), *t*(16.01) = 0.80, *p* = .438, *d* = 0.34. An exploratory analysis indicated that there was also no difference in RTD between the oldest children and the young adults, *t*(23.23) = 1.61, *p* = .121, *d* = 0.39.

**Figure 2.** Mean median reaction time in milliseconds (ms) for inhibitory and prepotent trials in the Early Childhood Inhibitory Touchscreen Task (ECITT) and the Early Childhood Inhibitory Touchscreen Task – Adult version (ECITT-A) in Study 4. The dashed line indicates a change to a more difficult task version (ECITT to the left of the line, ECITT-A to the right of the line). The bracket at the top indicates a significant planned contrast for the mean reaction time difference (RTD) score. Error bars indicate the standard error. *** *p* < .001, ** *p* < .01, * *p* < .05.

Discussion

Study 4 was carried out at five public engagement events, and involved the administration of a single ECITT/ECITT-A block to 140 participants. The results solidly replicated the effect of condition for both accuracy and RT, indicating poorer performance on inhibitory compared to prepotent trials. Developmental effects were somewhat weaker in this study, with only the predicted differences (in AccD and RTD) between 4- to 7-year-olds and 8- to 15-year-olds reaching significance. To some extent, this is likely due to the large variation in age group sizes in this opportunity sample (the sample was solely determined by the people attending public events who were interested in taking part) – the 4- to 7-year-old and 8- to 15-year-old groups were substantially larger than the other age groups (both had *N* > 50), providing more statistical power to detect effects. In contrast, the group of children under 4 years was small (*N* = 11), resulting in limited power to detect age progression in inhibitory performance between this group and the 4- to 7-year-olds. Having said that, the finding of significant developmental progression in response inhibition (as assessed by both AccD and RTD) between early primary and late primary school age does add further evidence for the suitability of the task as an appropriate developmental measure of IC, as this age range was not covered by Studies 1-3. This finding further strengthens the evidence for the task’s suitability for measuring developmental change in inhibitory control over a wide age range and in different contexts.
