# Supplementary material for: The early childhood inhibitory touchscreen task: A new measure of response inhibition in toddlerhood and across the lifespan
Source: PLoS One. 2021 Dec 2;16(12):e0260695. doi: 10.1371/journal.pone.0260695 (PMC8638877; doi:10.1371/journal.pone.0260695)
Supplement: S7 File — (DOCX) [file pone.0260695.s007.docx]

**S7 Supporting Information: Regression analyses of pooled cross-sectional data sets (Studies 1, 3, 4 and Pilot Study)**

**Analysis overview**

To get a full overview of developmental effects, a final set of regression analyses investigated the relation between age and inhibitory control as assessed by the ECITT and ECITT-A across the participant samples tested in Studies 1, 3, 4 and the Pilot Study (see S1 Supporting Information). Note that only cross-sectional data was included in this analysis; the longitudinal participants from Study 2 were not included to avoid mixing between-subjects and within-subjects data in the analysis. Pooling data not only maximised our sample size, but also allowed us to retain more information within our data, as participants were not divided into categorical age groups. We tested both a linear and a quadratic relation between Age and AccD/RTD. In accordance with Williams et al. ([1](#_ENREF_1)), we expected the quadratic relation to account better for the data, except in an analysis of data from children under 4 years only. A linear relation was likely to account better for the latter data because it covered a relatively short age span where only increases in performance were expected.

**Method**

Thirty-three of the public engagement participants only provided age in years (all > 4 years), therefore, we ran the regression analyses both with (*N* = 293) and without (*N* = 260) these participants. However, as this made little difference to the results (see S4 Table), we report the regression results from the full data set below. Seven toddlers were excluded from all analyses, as they were less than 60% correct on prepotent trials (for details on this exclusion criterion, see the Method section of Study 1). Coded data was used from lab-based studies and raw data was used from the public engagement study (applying the RT filters described in Study 1 and 3). Participants under 4 years were always administered the ECITT, whereas participants aged 4 years or older were administered the ECITT-A.

**Results**

*Accuracy difference (AccD)*

The results of the regression analyses are summarised in Table 1 below (all participants) and in S4 Table (excluding participants with age in years only). Scatterplots of the association between age and AccD can be seen in Figures 1a (participants < 4 years) and 1b (participants >= 4 years). As expected, across the entire sample, the quadratic function of age was a significant predictor of AccD, *β* = 0.84, *t*(290) = 3.66, *p* < .001. When the sample was split into under 4s (ECITT, *N* = 100, consisting primarily of toddlers from Study 1 and the Pilot Study) and older children and adults (ECITT-A, >= 4 years, *N* = 193), age effects on AccD were still evident. In under 4s, there was a significant linear relation between Age and AccD scores, *β* = -0.30, *t*(98) = -3.12, *p* = .002, indicating significant improvement in inhibitory control with age. In children aged 4 years or older, adults and older adults, a quadratic function accounted for an additional 4.7% of the variance in AccD, *β* = 1.05, *t*(190) = 3.13, *p* = .002, over and above the linear association (*β* = -0.20, *t*(190) = -2.80, *p* = .006). Overall, this pattern of results suggests that inhibitory control improved from childhood to adulthood and then diminished from younger to older adulthood.

**Table 1.** Hierarchical regression analyses of age as a predictor of Early Childhood Inhibitory Touchscreen Task (ECITT) and Early Childhood Inhibitory Touchscreen Task – Adult version (ECITT-A) accuracy difference (AccD) and reaction time difference (RTD) scores in all participants in Studies 1, 3, 4 and the Pilot Study. All participants under 4 years were administered the ECITT, and all participants aged 4 years or older were administered the ECITT-A. Seven toddlers were excluded from the sample because they had < 60% accuracy on prepotent trials.

**Figure 1a.** Scatterplot of ECITT accuracy difference (AccD) scores as a function of age across all toddler participants (< 4 years) in the Pilot Study, Study 1 and Study 4.

**Figure 1b.** Scatterplot of ECITT-A accuracy difference (AccD) scores as a function of age across all participants aged 4 years or older in Studies 3 and 4.

*Reaction time difference (RTD)*

As expected, across the entire sample the quadratic function of age was a significant predictor of RTD, *β* = 0.91, *t*(288) = 3.94, *p* < .001. When the sample was split into toddlers (ECITT) and older children and adults (ECITT-A), age effects on RTD were present only in the latter group. In children aged 4 years and older, adults and older adults, the data fit a quadratic function, *β* = 1.10, *t*(190) = 3.22, *p* = .001, with the quadratic function of age accounting for 5.1% of the variance in RTD. This relation can be seen in Figure 2. However, in children under 4 years of age, there was no linear relation between age and RTD scores, *β* < 0.01, *t*(96) = 0.01, *p* = .99.

**Figure 2.** Scatterplot of ECITT-A reaction time difference (RTD) scores as a function of age across all participants aged 4 years or older in Studies 3 and 4.

**Robust regression analyses**

*Tests for heteroscedasticity*

Inspection of Figures 1a, 1b and 2 suggests that the assumption of homoscedasticity may have been violated in the current data sets. Heteroscedasticity is indicated where the variability of the outcome variable (i.e., the error term) in the regression is unequal across the range of the predictor variable(s). Heteroscedasticity is indicated in Figure 1a because there is more variability in performance in younger toddlers than in older toddlers. In Figures 1b and 2, heteroscedasticity is indicated by higher variability in children and (to a lesser extent) older adults. If heteroscedasticity is present, robust regression provides less biased parameter estimates than ordinary least squares (OLS) regression ([2](#_ENREF_2)). We therefore tested whether the homoscedasticity assumption was indeed violated in the full data set (all cross-sectional participants included), as well as separately in children under 4 years and in participants aged 4 years or older. Note that where two predictors were investigated, i.e., the linear and quadratic age terms, both were entered into the analysis testing for heteroscedasticity.

Results indicated that for the full data set there was highly significant heteroscedasticity present for the AccD variable, according to both the modified Breusch-Pagan test (linear association between predictors and residuals assumed; 𝜒^2^(1, *N* = 293) = 12.70, *p* < .001) and White test (linear association between predictors and residuals not assumed; 𝜒^2^(4, *N* = 293) = 21.93, *p* < .001). Similarly, for RTD, the modified Breusch-Pagan test (𝜒^2^(1, *N* = 291) = 18.64, *p* < .001) and White test (𝜒^2^(4, *N* = 291) = 30.37, *p* < .001) were highly significant.

In children under 4 years, only the linear effect of age was entered (in accordance with our predictions for this age group). Again, for AccD, the modified Breusch-Pagan test (𝜒^2^(1, *N* = 100) = 5.74, *p* = .017) and White test (𝜒^2^(2, *N* = 100) = 7.70, *p* = .021) indicated significant heteroscedasticity. For RTD, the modified Breusch-Pagan test (𝜒^2^(1, *N* = 98) = 0.85, *p* = .77) and White test (𝜒^2^(2, *N* = 98) = 1.49, *p* = .48) were not significant, indicating the absence of heteroscedasticity. However, note that there was also no significant linear effect of age on RTD in children under 4 years of age (see Table 1).

Finally, in the regression analysis involving participants aged 4 years and older (ECITT-A), there was also significant heteroscedasticity. For AccD, the modified Breusch-Pagan test (𝜒^2^(1, *N* = 193) = 12.65, *p* < .001) and White test (𝜒^2^(4, *N* = 193) = 17.32, *p* = .002) were significant. For RTD, the modified Breusch-Pagan test (𝜒^2^(1, *N* = 193) = 4.64, *p* = .031) and White test (𝜒^2^(4, *N* = 193) = 10.44, *p* = .034) were also significant, although heteroscedasticity was less pronounced than for the AccD variable.

*Robust parameter estimates*

Because heteroscedasticity was an issue in most of the regression analyses presented in Table 1 above, we re-ran these analyses using robust regression. The results of this analysis are presented in Table 2. The robust parameter estimates can be compared to the OLS estimates in Table 1 to gain an idea of how seriously these estimates are biased by heteroscedasticity. As can be seen, although there were slight differences in the t-values, and the standard errors were smaller with robust estimation, all significant effects remained. In fact, in all cases where the linear and/or quadratic function of age were significant predictors of ECITT/ECITT-A performance, the effect was more significant with robust estimation (all significant *p*s <= .001 in Table 2, compared to mostly *p*s < .01 in Table 1). We therefore conclude that the linear and quadratic effects of age described above (in the sections ‘Accuracy difference (AccD)’ and ‘Reaction time difference (RTD)’) are robust even in the presence of significant heteroscedasticity.

**Table 2.** Robust regression parameter estimates for age as a predictor of Early Childhood Inhibitory Touchscreen Task (ECITT) and Early Childhood Inhibitory Touchscreen Task – Adult version (ECITT-A) accuracy difference (AccD) and reaction time difference (RTD) scores in all participants in Studies 1, 3, 4 and the Pilot Study. All participants under 4 years were administered the ECITT, and all participants aged 4 years or older were administered the ECITT-A. Seven toddlers were excluded from the sample because they had < 60% accuracy on prepotent trials. ^a^ See Table 1 (above) for other parameter estimates using ordinary least squares regression; ^b^ Using HC3 estimator.

**References**

1. Williams BR, Ponesse JS, Schachar RJ, Logan GD, Tannock R. Development of inhibitory control across the life span. Dev Psychol. 1999;35(1):205-13.

2. Hayes AF, Cai L. Using heteroskedasticity-consistent standard error estimators in OLS regression: An introduction and software implementation. Behav Res Methods. 2007;39(4):709-22.
