# Supplementary material for: The early childhood inhibitory touchscreen task: A new measure of response inhibition in toddlerhood and across the lifespan
Source: PLoS One. 2021 Dec 2;16(12):e0260695. doi: 10.1371/journal.pone.0260695 (PMC8638877; doi:10.1371/journal.pone.0260695)
Supplement: S8 File — (DOCX) [file pone.0260695.s008.docx]

**S8 Supporting Information: ECITT demo instructions**

You will require two separate devices (tablet (with a sturdy casing and tempered glass screen protector) and smart phone recommended, but a computer/laptop can also be used) to run the ECITT, using one to control the task (‘the Controller’) and the other to collect responses from participants (‘the Responder’).

1. On both chosen devices, open a web browser and navigate to <https://ecitt.app>

Additional setup for first-time use:

If using an iPad as the Responder, you will need to set up a screen lock for ‘Guided Access’. This is especially important when testing infants and young children as it prevents them from being able to press anything other than the task stimuli. Before running the task, triple-press the home button to bring up the Guided Access page. Circle around the search bar and tabs, ensuring all these areas are included in the final grey area. Select ‘Start’, then enter a passcode and verify. Guided access will be started, and you will no longer be able to interact with your locked area. To end guided access, triple-press the home button again and enter the passcode, then select ‘End’. Whenever guided access is subsequently used, it will automatically lock your selected area.

It is also recommended that the top and bottom on the iPad and case are covered to prevent young children getting distracted by the case and search/tool bar. Once you have navigated to the webpage and no longer need to interact with the search bar, use thick duct tape to cover the top (including the camera and search bar) and bottom (including the home button and edge of the case) (see image below for an example of a cased and taped iPad used as Responder).


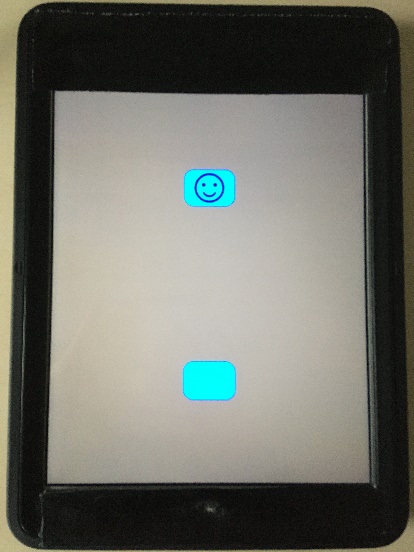


**iPad preparation:** example casing and taping of iPad used as Responder, with vertical ECITT task

1. On both devices, enter the username ‘guest’ and the password ‘demo’, then select ‘Sign in’
2. Select ‘Controller’ on the device you have chosen to be your controller, and ‘Responder’ on the device you have chosen to be your responder
3. You will be presented with a pairing code on the Responder – input this into the Controller, then select ‘Connect’ (this avoids different users interfering with each other, which can cause disconnection issues).
4. On the Controller: select ‘Tasks’. In the drop-down menu, select ‘demoProject’ and ‘Enter’. In the drop-down menu, select ‘demoTestSet’ and ‘Enter’. If the participant is already in the app, select them from the drop-down list, otherwise:

- Select ‘Add new’ and input participant details (participant ID, date of birth, and sex) and ‘Save’.
- **NB: Any data collected via the ECITT demo app will be publicly available. Do not use for real data collection. We strongly suggest entering dummy information for date of birth as this is potentially identifying.**

1. On the Controller: in the drop-down menu, select the appropriate test and ‘Enter’.

- ‘ecittv’ is the vertical version, ‘ecitth’ is the horizontal version, and ‘ecittav’ and ‘ecittah’ are the adult versions of the vertical and horizontal ECITT, respectively

1. On the Controller: select the required Trial Set.

- Participants are assigned a specific location to build a prepotent response (‘the prepotent location’)
- In the vertical versions, ‘Test PR Top’ will present the prepotent location as the top button and ‘Test PR Btm’ will present the prepotent location as the bottom button
- In the horizontal versions, ‘Test PR Lft’ will present the prepotent location as the left button and ‘Test PR Rgt’ will present the prepotent location as the right button

**ECITT (infant/young child)**

Administration guidance:

For all trials, hold the tablet at a slight angle to make it easy for the child to reach both locations (see images below showing both the vertical and horizontal version administration). Move it back during the cartoon so they do not accidentally/impulsively touch before the next trial, but try to have the screen back in front of them by the time the next trial starts *(this is essential for getting accurate RTs and for retaining valid trials)*. If the child is very eager/“grabby”, place the iPad gently on top of the child’s hand as you bring the screen back (to prevent a premature touch). If a child produces very few (1-2) impulsive responses, it is okay to keep the screen in front of them, but if they start responding impulsively revert to taking the screen back during the cartoon.

1. On the Controller: Select ‘x1’ under ‘Practice Demo’. While pointing to the smiley face, say “Can you see the happy face? Look what happens when we touch it”. Touch the blue button and show the child the cartoon.
2. On the Controller: Select ‘x1’ under ‘Practice Trial’. Say “Can you touch/press the happy face?”. If the child is very reluctant to touch, add 1 or 2 practice trials after the first one.
3. On the Controller: Select ‘x32’ under ‘Test Trial’. As soon as blue buttons appear on the first test trial, clearly cue (point to) the correct location with your finger, making sure that the child attends. No further cueing on subsequent trials.
4. (Horizontal version only (Hendry et al., 2021, <https://psyarxiv.com/mhkaj/>): If the child shows a strong side bias (selecting the incorrect location even on the first trial with cueing), restart the test trials and swap the prepotent side to align with the child’s initial bias.)
5. In the original version of the ECITT (Holmboe et al., 2021, <https://psyarxiv.com/k7g4a/>, Study 1), if the child makes an incorrect response, both buttons disappear and the next trial is presented. In later versions of the task (e.g., Study 2), the incorrect button remains visible after touching, and the child has to press the correct button before the next trial is presented.

There is a total of 32 trials. It is best to try and get the participant through all 32 trials, especially if they are over 2 years old. Instruction (“can you touch the happy face”) can be repeated as needed. If perseveration is very strong on a specific trial (i.e., for 30+ sec) and the child starts disengaging from the task because of it, cue the correct response to keep them interested (this trial will be coded as invalid). If still no touch after 5 seconds, show them (touch the correct location) – this should rarely be needed.


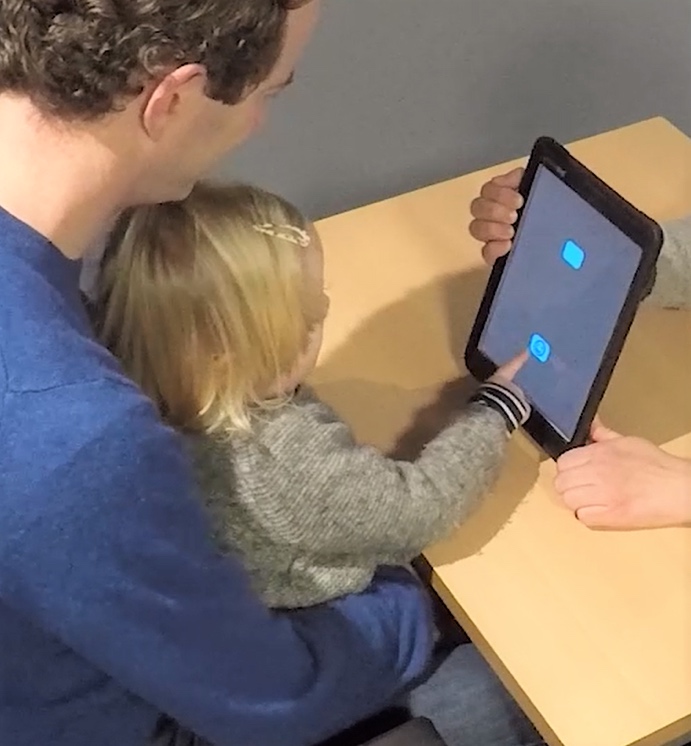

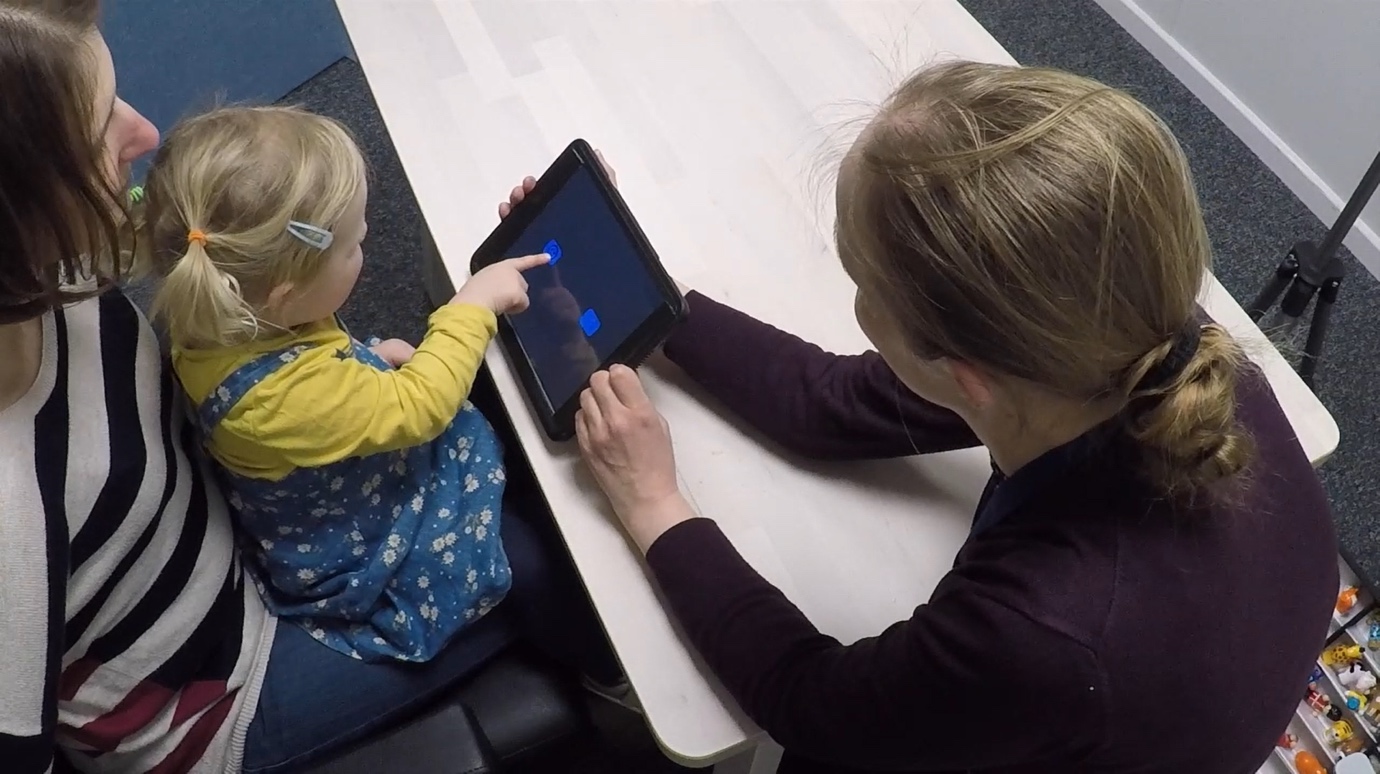


**ECITT administration:** Left picture: vertical stimulus presentation. Right picture: horizontal stimulus presentation. In Holmboe, Larkman, de Klerk, Simpson, Bell, Patton, Christodoulou, & Dvergsdal (2021) only the *vertical* version was used.

**ECITT-A**

Administration guidance:

For all trials, place the tablet on a stand at an angle or hold the tablet at a slight angle in front of participants. Keep the iPad steady and in the same location throughout the trials.

1. Instruct participants “Put your finger on the red dot. When you see the buttons, press the happy face as fast as you can. Then return to the red dot”
2. On the Controller: Select ‘x4’ under ‘Prtc PR *Top/Btm/Lft/Rgt*’ and allow the participant to complete the 4 practice trials.
3. On the Controller: Select ‘End’ then reselect the test set so that the instructions and red dot reappear. Select ‘x32’ under ‘Test PR *Top/Btm/Lft/Rgt*’ and allow the participant to complete the test trials. The task will automatically end after 32 trials, when participants will be shown their reaction time along with a short animation. If more than one block is administered, say to participants: “Please try to respond faster in the next block. But also remember to respond as accurately as possible.”

Controller count:

On all versions of the ECITT task, the Controller will display the total number of trials as well as the number of correct and incorrect trials while the task is being completed. This is displayed next to each trial set. The white number represents the total number of trials completed, the green number represents the number of correct responses, and the red number represents the number of incorrect responses.

For questions relating to the ECITT, ECITT-A and the administration of these tasks, please contact Dr. Karla Holmboe on [karla.holmboe@bristol.ac.uk](mailto:karla.holmboe@bristol.ac.uk)
