# Supplementary material for: The early childhood inhibitory touchscreen task: A new measure of response inhibition in toddlerhood and across the lifespan
Source: PLoS One. 2021 Dec 2;16(12):e0260695. doi: 10.1371/journal.pone.0260695 (PMC8638877; doi:10.1371/journal.pone.0260695)
Supplement: S1 Protocol — (DOCX) [file pone.0260695.s009.docx]

**S1 Protocol: ECITT testing protocol**

1. Open the Responder app on the iPad (downloadable from the Apple store) and navigate to <https://ecitt.app> on the iPhone (or other device used as ‘Controller’). The responder can also be run directly in a browser (i.e., without downloading the Responder app).
2. Sign in to both the iPad and iPhone using the same log in details (e.g., ‘guest’ and ‘demo’)
3. On the iPad, select ‘Make Available’ and on the iPhone select ‘Connect’.

NB: if the iPad does not make a noise when you select ‘Make Available’, there will be no audio. Sign out and back in again.

1. On the iPhone, select ‘Tests’, then navigate to the correct Project and Test Set and press ‘Enter’.
2. If the participant details have not been entered into the app before, select ‘Add new’ and enter the participant ID, date of birth and gender, then ‘Save’. If the participant has previously been saved in the app, search for the participant ID and select from the drop-down list. Press ‘Enter’. *Please do not use identifying information when using the demo version of the ECITT Web App, data will be publicly available.*
3. Select task version from the drop-down list, and then ‘Enter’.
4. The participant is assigned a location to build up a prepotent response (prepotent location). Select the appropriate option for up/down or left/right (there is currently a horizontal and vertical version of the task).

Administration guidance:

For all trials, hold the tablet at a slight angle to make it easy for the child to reach both locations (see images below showing both the vertical and horizontal version administration). Move it back during the cartoon so they do not accidentally/impulsively touch before the next trial, but try to have the screen back in front of them by the time the next trial starts *(this is essential for getting accurate RTs and for retaining valid trials)*. If the child is very eager/“grabby”, place the iPad gently on top of the child’s hand as you bring the screen back (to prevent a premature touch). If a child produces very few (1-2) impulsive responses, it is okay to keep the screen in front of them, but if they start responding impulsively revert to taking the screen back during the cartoon.

1. On Controller: Select ‘x1’ under ‘Practice Demo’. While pointing to the smiley face, say “Can you see the happy face? Look what happens when we touch it”. Touch the blue button and show the child the cartoon.
2. On Controller: Select ‘x1’ under ‘Practice Trial’. Say “Can you touch/press the happy face?”. If the child is very reluctant to touch, add 1 or 2 practice trials after the first one.
3. On Controller: Select ‘x32’ under ‘Test Trial’. As soon as blue buttons appear on the first test trial, clearly cue (point to) the correct location with your finger, making sure that the child attends. No further cueing on subsequent trials.
4. (Horizontal version only (Hendry et al., 2021, <https://psyarxiv.com/mhkaj/>): If the child shows a strong side bias (selecting the incorrect location even on the first trial with cueing), restart the test trials and swap the prepotent side to align with the child’s initial bias.)
5. In the original version of the ECITT (Holmboe et al., 2021, <https://psyarxiv.com/k7g4a/>, Study 1), if the child makes an incorrect response, both buttons disappear and the next trial is presented. In later versions of the task (e.g., Study 2), the incorrect button remains visible after touching, and the child has to press the correct button before the next trial is presented.

There is a total of 32 trials. It is best to try and get the participant through all 32 trials, especially if they are over 2 years old. Instruction (“can you touch the happy face”) can be repeated as needed. If perseveration is very strong on a specific trial (i.e., for 30+ sec) and the child starts disengaging from the task because of it, cue the correct response to keep them interested (this trial will be coded as invalid). If still no touch after 5 seconds, show them (touch the correct location) – this should rarely be needed.


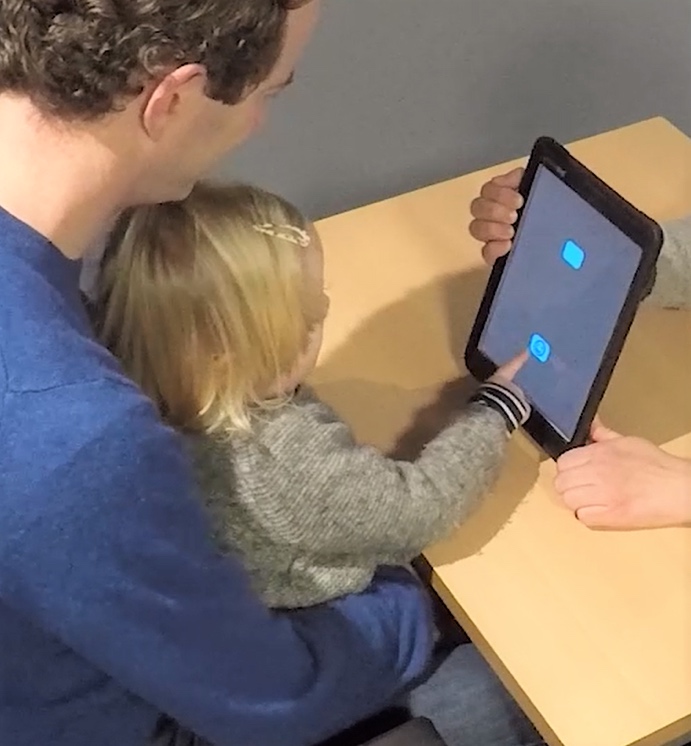

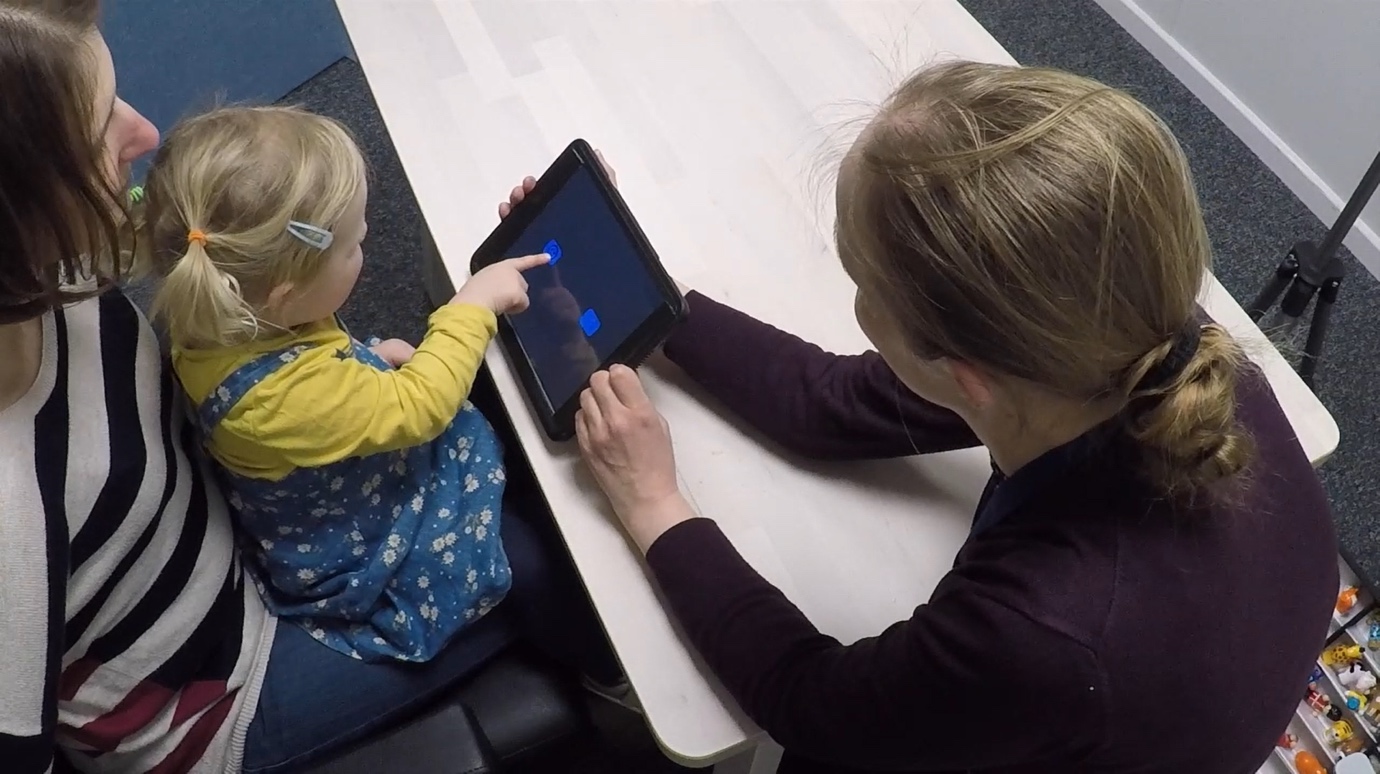


**ECITT administration:** Left picture: vertical stimulus presentation. Right picture: horizontal stimulus presentation. In Holmboe, Larkman, de Klerk, Simpson, Bell, Patton, Christodoulou, & Dvergsdal (2021) only the *vertical* version was used.
