# Supplementary material for: The early childhood inhibitory touchscreen task: A new measure of response inhibition in toddlerhood and across the lifespan
Source: PLoS One. 2021 Dec 2;16(12):e0260695. doi: 10.1371/journal.pone.0260695 (PMC8638877; doi:10.1371/journal.pone.0260695)
Supplement: S2 Protocol — (DOCX) [file pone.0260695.s010.docx]

**S2 Protocol: ECITT coding protocol**

This protocol can be used for coding test sessions with children from 15 months of age. The protocol has been used down to 10 months of age for the horizontal version of the task (although RTs are not coded at this age, except to check for validity).

Add new data to be coded

1. Export the latest data from <https://hdim.no/ecitt/datamgr/>Responses
2. Open a new Excel workbook
3. Copy and paste column headers from existing sheets or follow instructions below to set up new workbook
4. Save as YourName_ECITT_Study

Pre-coding prep

**Column V**: mark the location of the target on the first test trial (prepotent location)

**Column W:** ‘Validity_basedonRT’. Paste in the formula =IF(S2<300,0,1) and drag down

- - Check the results make sense - trials with a respTime less than 300 should show as 0 in the valid column. All other trials should be 1.

**Column X:** ‘Accuracy_basedonvideo_XX’ (where XX are your initials)

**Column Y**: ‘Validity_basedonvideo_XX’

**Column Z:** Reason invalid (see codes below)

**Column AA:** ‘Terminated early?’ *Fill in at the final trial completed*

- - Input 0 if 20 trials were completed
  - Input 1 if there were fewer than 20 trials

**Column AB:**  ‘Reason terminated?’

Indicate reason (see below)

**Column AC:** ‘Held back?’. Input L if left hand/arm held by parent. Input R if right arm/hand held by parent. If not held leave blank.

**Column AD:** ‘Override software’. Input Y for yes, leave blank if no.

**Column AE:** ‘Reason overwritten’

Select from list of reasons for overwriting software **(see codes below)**

***Column AF:*** *‘Trial start (frame time)’ - On Webcoder (this refers to custom-made frame-by-frame video coding software), navigate to the first frame of the task trial (e.g. the first frame in which the two blue squares appear for ECITT) and note down the frame number.*

***Column AG:*** *‘Response (frame time)’ - Navigate to the first frame in which the baby makes their response (e.g. the first frame in which the baby touches one of the squares in ECITT) and again note down the frame number.*

**Column AH:** ‘Corrected RTs.’ Calculate correct RT in cases where software does not detect first response (see notes below). *If Webcoder was used (I.e. if Column AF and AG is filled), type “= 33*(AG4-AF4)” to calculate the correct RT.*

**Column AI:** ‘Retain RT as >5000ms’ Type =IF(S2>5000,0,1) into first row and drag down. Helps distinguish long RTs as these won’t be included in RT analysis, but will be included in accuracy.

**Column AJ:** ‘Notes’ - make notes on any difficult trials that you can then discuss with another team member.

**Column AK:** ‘Final corrected RT’- copy the data from column S ‘respTime’ and paste here, change the data in this column when you correct any reaction times

**Column AL:** ‘Coder initials’ add initials and drag down so that every row has coder initials

Coding protocol

*Note that if the infant is incorrect the trial does not end until the happy face is touched…. this can take some time. Only start coding accuracy and validity again for the next trial (i.e. after a cartoon has been played).*

Starting from the first test trial (trialType tpr, which would normally begin on row 4), fill in columns X and Y (and Z if invalid – see possible reasons below).

**Accuracy** (column X) is either 0 (incorrect) or 1 (correct). Accuracy is taken from the first response (defined as bash, touch, tap, scratch etc.) to or near a target (blue button with or without happy face)

- Note: the response could count as dragging their hand over the screen, scratching/scraping the screen, bashing with their full hand consistently at one of the target locations.
- If the infant touches the middle or any other area OUTSIDE of the target area this is not considered a RESPONSE. CODE NEXT RESPONSE.
- **If the child taps outside a hit area and then seems to change direction, we have to take the first response from the first tap within the hit area.**
- When the infant touches the wrong location for balance but is clearly looking at the correct location and touches it on their next response, code their second response as opposed to their first touch (i.e. the balancing touch) and overwrite software accuracy.

**Validity**: 0 = invalid. 1 = valid. If invalid specify reason code in column Z.

- IMPORTANT: If child has finger on one of the buttons (or within the touch sensitive area) at stim onset, the trial is always invalid.
- **First trial is ALWAYS invalid.**
- **Validity (based on RT):** Paste in the formula =IF(S2<300,0, 1) and drag this down the column. Check the results make sense - trials with a respTime less than 300 should show as 0 in the valid column. All other trials should be 1.

**Reaction Time Correction:**

Column AG: ‘Retain RT as >5000ms’; Type =IF(S2>5000,0,1) into first row and drag down. Check the results make sense – trials with a respTime more than 5000 should be shown as 0 in the column, all other trials shown as 1.

Note that long RTs (>5000) are excluded from analysis but included in accuracy data (column AG)

*The corrected RT is the latency from onset (blue box first appearing) until offset (touching the hit area of the screen). Aim for accuracy to the nearest frame (recording at 30 fps (frames per second) this gives an accuracy to within 33ms).*

Reaction times need to be corrected if:

- - An accuracy override code was used (Y – A, B, C, D, E)
  - The software did not detect child’s first touch
  - The software did not register the first touch and researcher has to press to trigger animation (only on correct trials)

Reaction times do not need to be corrected if:

- - The trial is already <300ms (I.e. 0, 3) (NB: if AFTER correcting RT the trial is <300ms, then this would make the trial invalid)
  - Hand was on screen during stimulus onset (I.e. 0, 12). In this case, correct RT to 0ms.
  - If touch time and response time are very close (1-3 frames apart)

When a RT is corrected:

- Override the original reaction time with the corrected reaction time in column **AF and AI.**
- Highlight the correct RT in green (Accent 6, lighter 60%) in column **AF and AI.**
- Highlight the Column AG: ‘Retain RT as >5000ms’ in green (Accent 6, lighter 60%) ONLY if the new RT then will change the outcome of the exclusion criteria. This makes it clear as to whether new RT impacts exclusion RT criteria.

Corrected RT could either be taken from the:

- **TOUCH TIME:**
  - This occurs when the first tap is the first intentional response.
  - An intentional response is defined as the first response (bash, touch, tap, scratch etc.) to, or near, a target area (blue button with or without happy face)**.**
  - This differs from an unintentional or accidental response, which is where a response is made to, or near, a target area, but this has happened by accident. Examples of accidental responses are detailed under Invalidity Reason 4.
- **RT SOFTWARE:**
  - When the first tap is not the first intentional response: use RT software to find trial start frame and trial end frame. Imput data into columns AG and AH, and work out final RT for column AH.
  - When touch time and response time are both over 5000ms: work out the response time to see if the corrected time is under 5000ms, and therefore could be corrected. If it is very obvious that the first touch is well over 5000ms (e.g. if a break was taken) then there is no need to correct, but mention the reason for not correcting in the coding notes.
  - When researcher presses to trigger animation as software did not detect child’s response (despite being accurate): use RT software to count frames and work out ms for corrected RT. This is because the RT recorded by software will be the researcher’s touch and not the child’s touch. In some cases, touch time can be used however RT software may be needed in others.

**Reason invalid**

**1 =** Taps with 2 hands simultaneously (as an intentional response, i.e., touching a response area, rather than the edges or case of the screen)

- Use this sparingly, often one hand does slightly lead and the other one is just being used for balance. Go with what is touched first and don’t use invalidity code.
- Code accuracy as per the software.
- If an intentional 2-hand response is made where both hands simultaneously touch the screen, then it is invalid. This is because it is impossible to tell what response the participant wanted to make. Use only when very clear that response was intentional and simultaneous.
- *Note*: A child’s preferred response style may be a bi-manual response to the screen. This is an intentional response, and the child will often respond this way across a number of trials. It is likely that the experimenter would have asked the parent to gently hold back one of the child’s hands at this point to be able to distinguish responses. This is different from two-handed bashing to the screen which is an unintentional/accidental response, and is done as a result of fussing or boredom (see Invalidity Code 4).

**2** = Code no longer in use (do not use)

**3** = Still had hand in position from previous tap (or already reaching before the new set of stimuli had been shown), so the response is too quick to be in response to the stimuli.

- Note that this will often be accompanied by a short RT: Only exclude as invalid if <300ms

**4** = Infant clearly not attending and tapped by **accident.**

- Examples of accidental tapping include but are not limited to: looking at E1 or mum when making a response, touched the screen when bashing and fussing, fidgeting with the tape around the tablet (used to cover home button and camera on iPad), not looking at the screen when responding, touching the screen to balance, catching the screen with hand when moving tablet or when E1 moves tablet.
- (At 10m – this often happens when infant is trying to push/pull tablet away and then accidentally draws hand across screen – thus there is a response, but it's not deliberately directed to a stimulus)
- Bimanual bashing (often multiple times), if child is bashing/touching screen while not looking (e.g., head turned at least 90° away from the screen) should be coded as invalid (accident).

**5** = Parent interference

- Points/nudges or otherwise indicates which one to touch

**6** = Researcher pressed

- This may occur during the first few trials if the researcher thought they needed another cue, or by accident later in the task

**7** = Reaching behaviour obscured

- (e.g. child stands on mum’s lap, so screen and reach are just out of range of video).
- ONLY USE WHEN RESPONSE IS STILL UNCLEAR ON CAMERA 2 or 3 – often you can use Camera 2 or 3 to check the looking behaviour/motor response of the infant to guide judgement. If infant is clearly orienting towards the top or bottom of the tablet, use this to deduce whether they made a correct / incorrect response^[[1]](#footnote-2)^

**8** = Used parent’s hand

**9** = Experimenter interference

- Experimenter directly points to or says which location is correct/incorrect (includes initial prompt on the first trial)
- Experimenter stops incorrect response in progress (child about to make response but stops because the experimenter indicates that this is incorrect).

**10** = On first prepotent trial infant tapped the opposite location so the researcher then restarted with the opposite location marked as prepotent

**11 =** Experimenter changes prepotent location halfway through the trial – trial numbers in column M will reset to number 1

**12 =** If child has finger/hand/both hands on one, or both, of the stimuli (or within a touch sensitive area) **at or before stimulus onset** then the trial is **always** invalid. Override software when needed.

**13 =** Child touches middle of screen. This is a clear intentional response (e.g., not an accident) but it is impossible to tell which stimuli the child intended to touch. Software will make its own decision. Code accuracy with software, but invalid.

**14** = Infant responds using area of body that is not their hands (e.g. nose, forehead, foot, mouth, elbow etc.)

**Notes on Validity**

- It’s okay for the child to turn away for any duration, including at the beginning of trials (e.g., at stim onset).
- Trials with long RTs will be excluded in Step 2 for RT analyses, but for Accuracy it is okay to include these trials.
- If the infant gets stuck on an inhibitory trial (i.e. they make an incorrect response, but they can’t/won’t move on), the experimenter may tap the correct location to trigger the cartoon and the next trial. This is fine as this response doesn’t affect the accuracy data.
- If a trial is invalid, still code the accuracy as if it had been valid (for example if the infant inadvertently swipes the tablet, record whether it was on the correct location or not)

**Reason Overriding iPad recorded Data**

*NB: Child’s actual behaviour observed on the video will ALWAYS override software. It is sometimes the case that the software does not detect the child’s first response to a target area – mostly in this case (if accurate), the experimenter will touch for the child. Always keep comments in the ‘Notes’ column when this happens*

In cases where you need to manually override the software, record a ‘Y’ for yes in column ‘AD’

Provide a reason for overwriting the software in column ‘AE’

Highlight where you have overwritten in your own coding columns and also in ‘accu’ column on software (Column P) - so that we can check this with another coder.

**A** = Undetected touch as hand was still on screen: touching the animation/before the next stimulus was presented/before stimulus onset.

**B** = View obscured – parent’s head or hair blocking the view of the infant

**C =** Software did not detect first touch

**D =** Part of the infant’s hand touched a stimulus sensitive area (due to hand position or swiping on screen e.g. little finger catches the other stimulus or hand swiped across stimulus to make response), but clearly aiming for opposite stimuli – only use when this is very clearly the case.

**E =** infant touches the wrong location for balance but is clearly looking at the correct location and touches it on their next response (e.g., immediately after). Code their second response as opposed to their first touch (i.e. the balancing touch) and overwrite software accuracy. This code is not used for the vertical version of the task because participants are very unlikely to touch one of the locations for balance with the vertical stimulus arrangment.

**Reason Terminated Early**

**1** = **Refused to respond to touchscreen** [use this code if they stop touching the screen]

**2** = **Fussiness** [use this code if the infant was still responding but the researcher could see they were tired or upset so stopped anyway]

**3** = **Equipment error** [e.g. tablet stopped registering responses or app crashed]

**4** = **Experimenter error** [e.g. no obvious infant/equipment reason, but the experimenter stopped before 20 trials]

The touch sensitive response area around the buttons is as follows:

**Button size on the iPad: 24 x 13 mm**

**Response area around the buttons (where touches are detected): 44 x 44 mm**


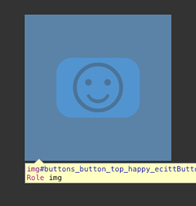


1. Only relevant when also using a front-facing camera and an additional elevated side-view camera. [↑](#footnote-ref-2)
