# Supplementary figures and images for: The early childhood inhibitory touchscreen task: A new measure of response inhibition in toddlerhood and across the lifespan
Source: PLoS One. 2021 Dec 2;16(12):e0260695. doi: 10.1371/journal.pone.0260695 (PMC8638877; doi:10.1371/journal.pone.0260695)

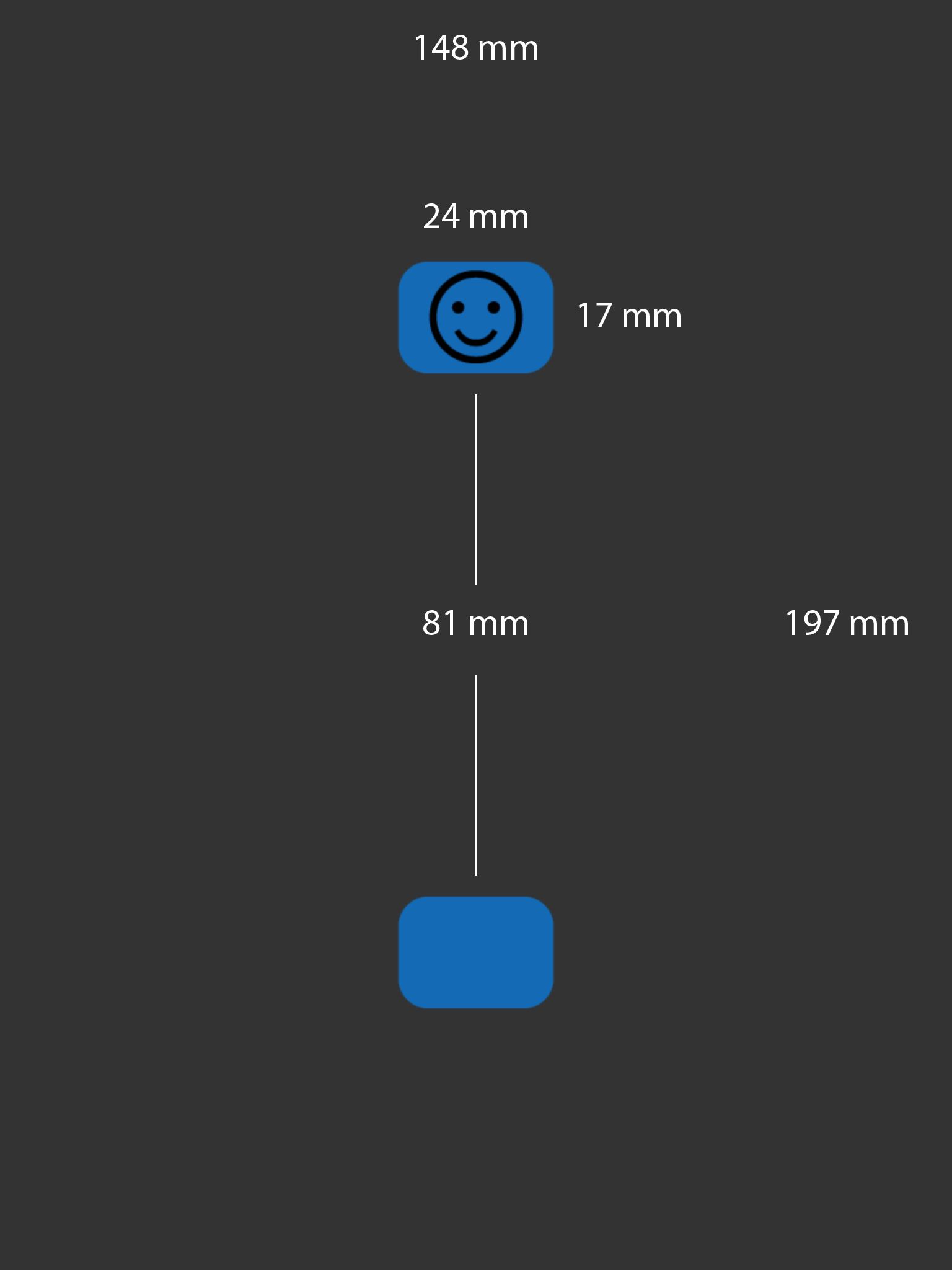


**S1 Figure.** Stimulus parameters in the Early Childhood Inhibitory Touchscreen Task

Supplement: S1 Fig — (DOCX) [file pone.0260695.s011.docx]
