# Supplementary material for: The early childhood inhibitory touchscreen task: A new measure of response inhibition in toddlerhood and across the lifespan
Source: PLoS One. 2021 Dec 2;16(12):e0260695. doi: 10.1371/journal.pone.0260695 (PMC8638877; doi:10.1371/journal.pone.0260695)
Supplement: S2 Fig — (DOCX) [file pone.0260695.s012.docx]

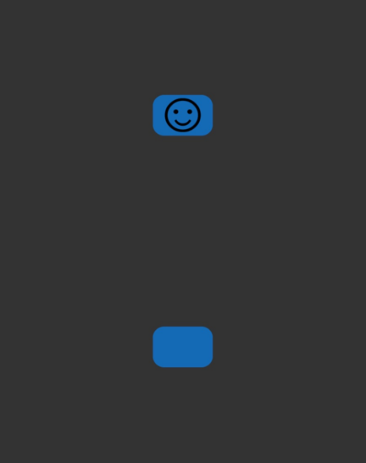

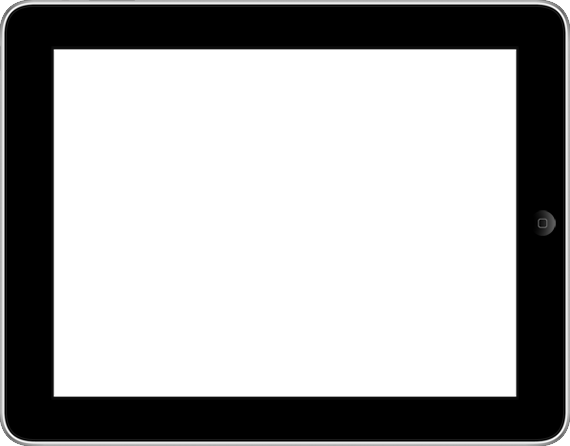

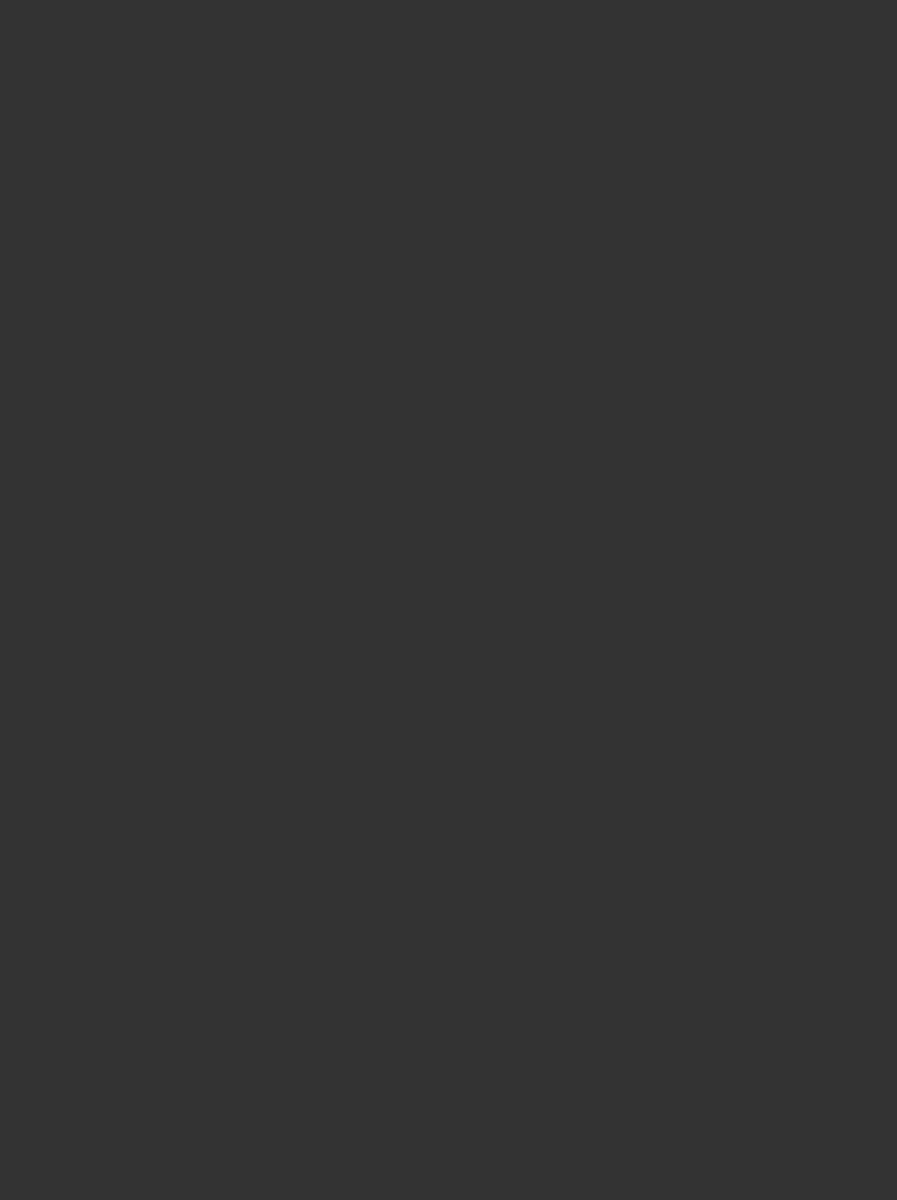

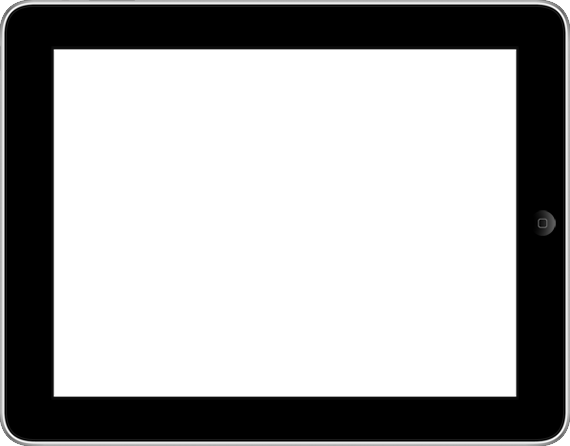

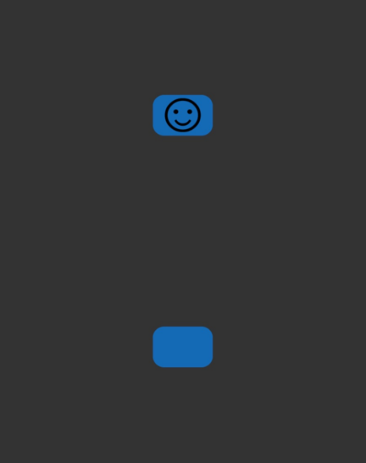

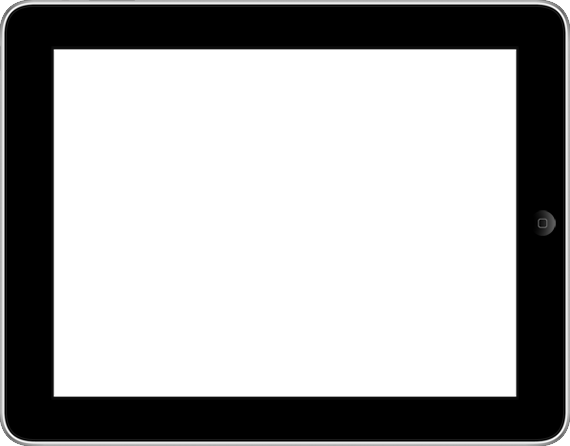


**
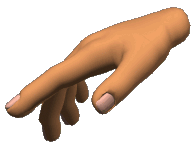
**


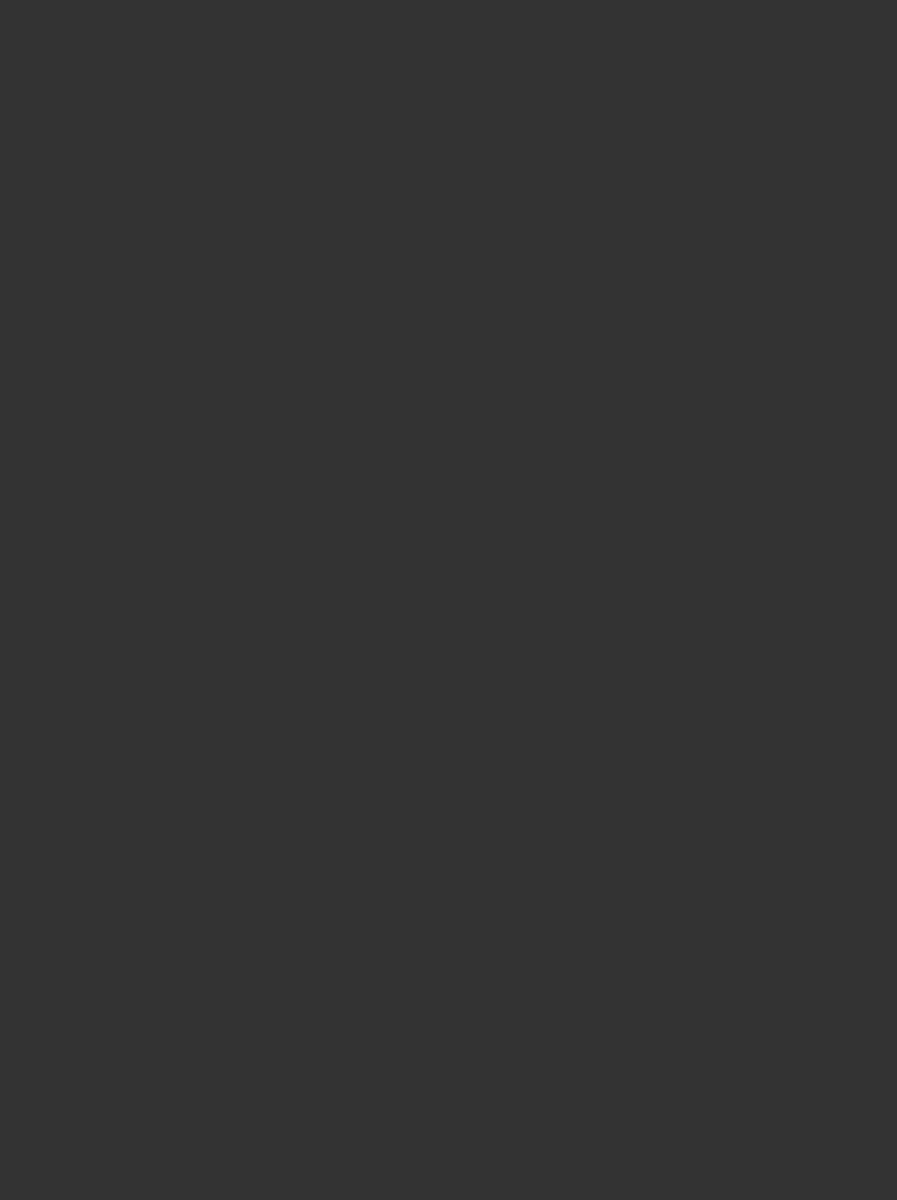

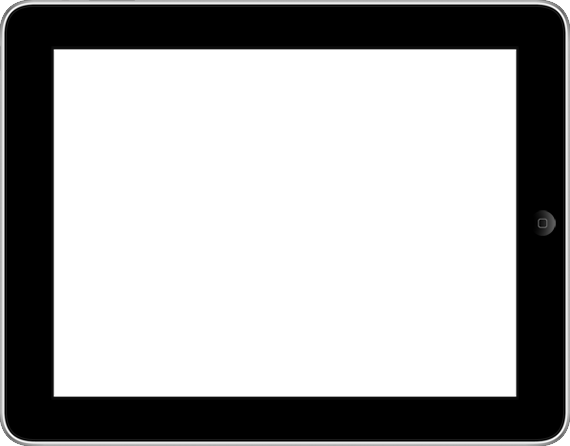

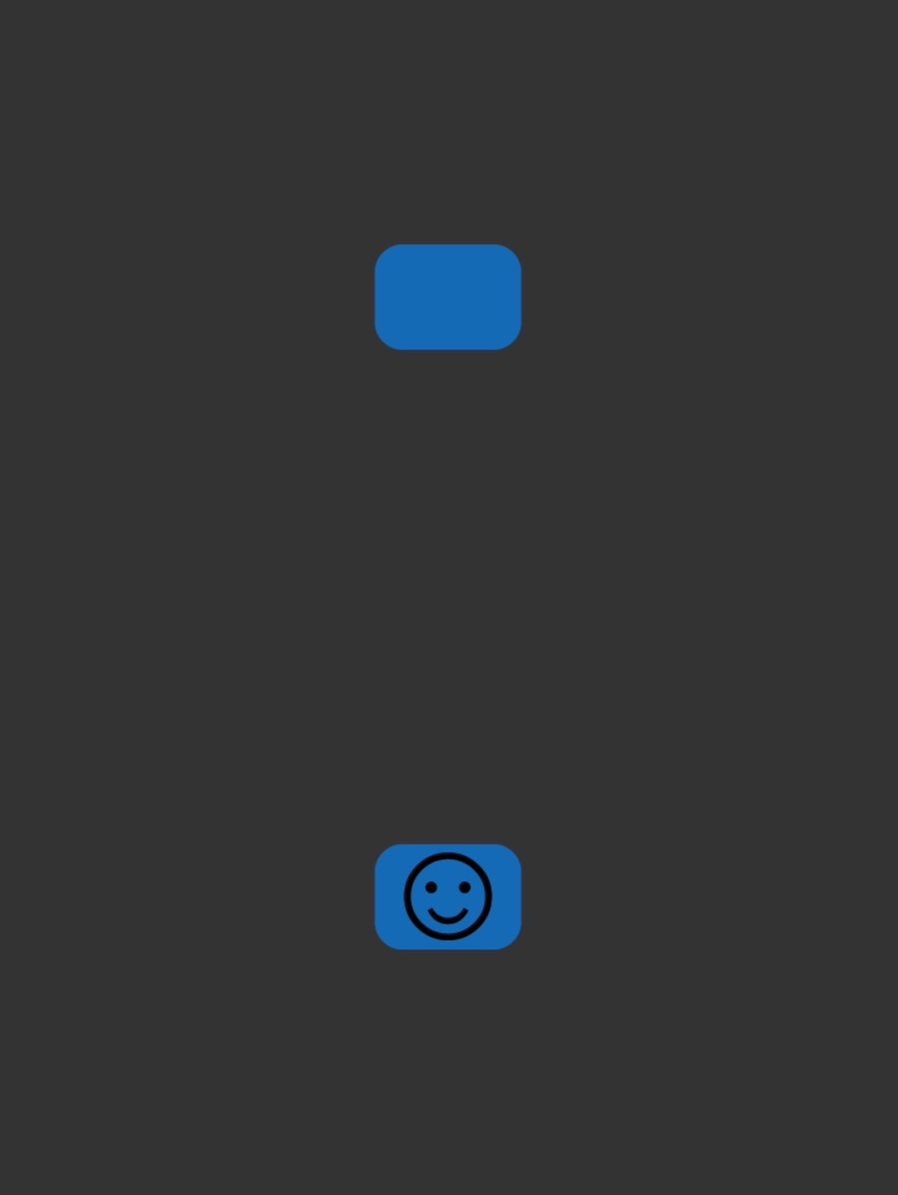

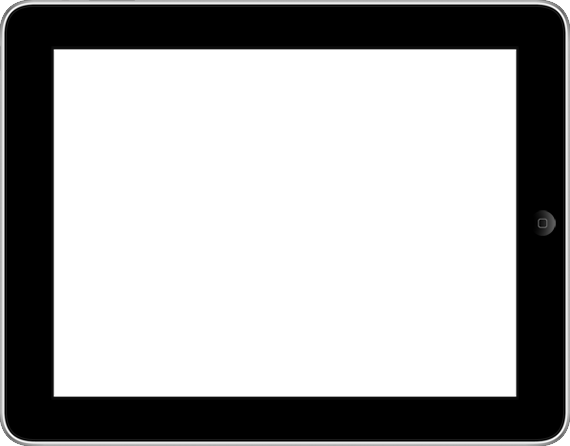


**
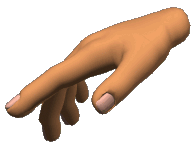
**


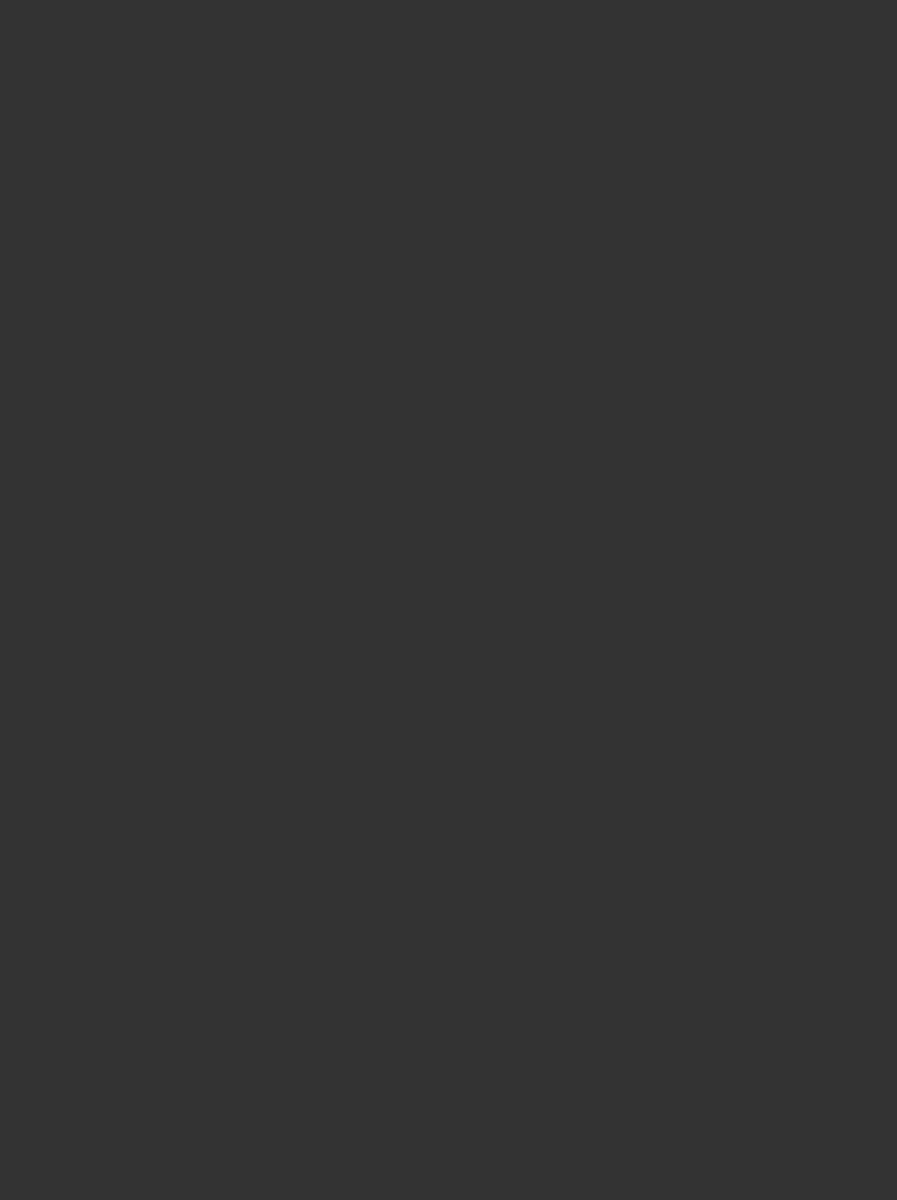

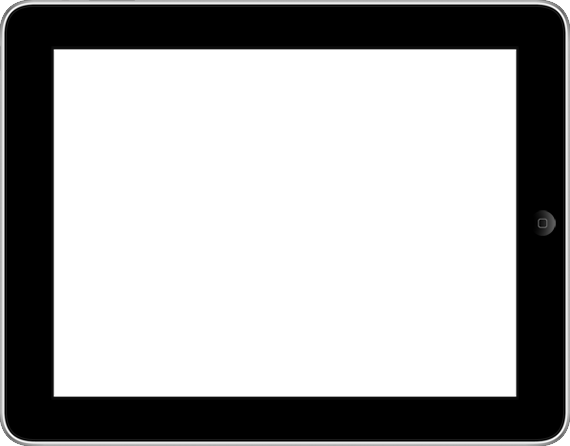


**
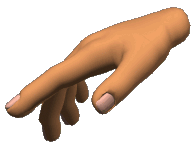
**

**Prepotent trial**

**1000 ms ITI**

**Finger on red dot**

**Prepotent trial**

**1000 ms ITI**

**Finger on red dot**

**Inhibitory trial**

**1000 ms ITI**

**Finger on red dot**

**S2 Figure.** Illustration of the Early Childhood Inhibitory Touchscreen Task – Adult version (ECITT-A). ITI: inter-trial interval.
