# Supplementary material for: The early childhood inhibitory touchscreen task: A new measure of response inhibition in toddlerhood and across the lifespan
Source: PLoS One. 2021 Dec 2;16(12):e0260695. doi: 10.1371/journal.pone.0260695 (PMC8638877; doi:10.1371/journal.pone.0260695)
Supplement: S3 Fig — (DOCX) [file pone.0260695.s013.docx]

**
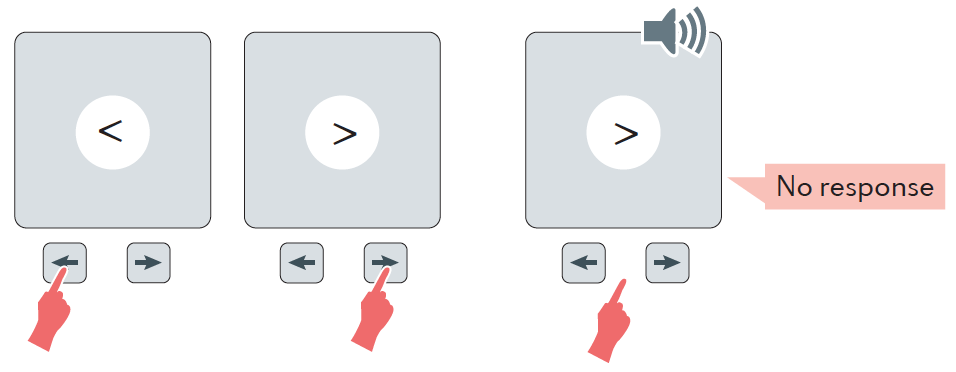
**

**S3 Figure.** Illustration of the Stop-signal task procedure. Participants have to press the right or left button corresponding to the arrow displayed on the screen. However, if a brief tone is played after the presentation of the arrow, the participant has to inhibit their response. Figure from: Dalley JW, Robbins TW. Fractionating impulsivity: neuropsychiatric implications. Nat Rev Neurosci. 2017;18(3):158-71 (p. 160, Figure 1d).
