# Supplementary material for: The early childhood inhibitory touchscreen task: A new measure of response inhibition in toddlerhood and across the lifespan
Source: PLoS One. 2021 Dec 2;16(12):e0260695. doi: 10.1371/journal.pone.0260695 (PMC8638877; doi:10.1371/journal.pone.0260695)

**S4 Figure.** Scatterplot showing the correlation (*r* = .35, *p* < .01) between ECITT accuracy difference (AccD) score and Stop-signal reaction time (SSRT) in children and adults in Study 3 (*N* = 62).


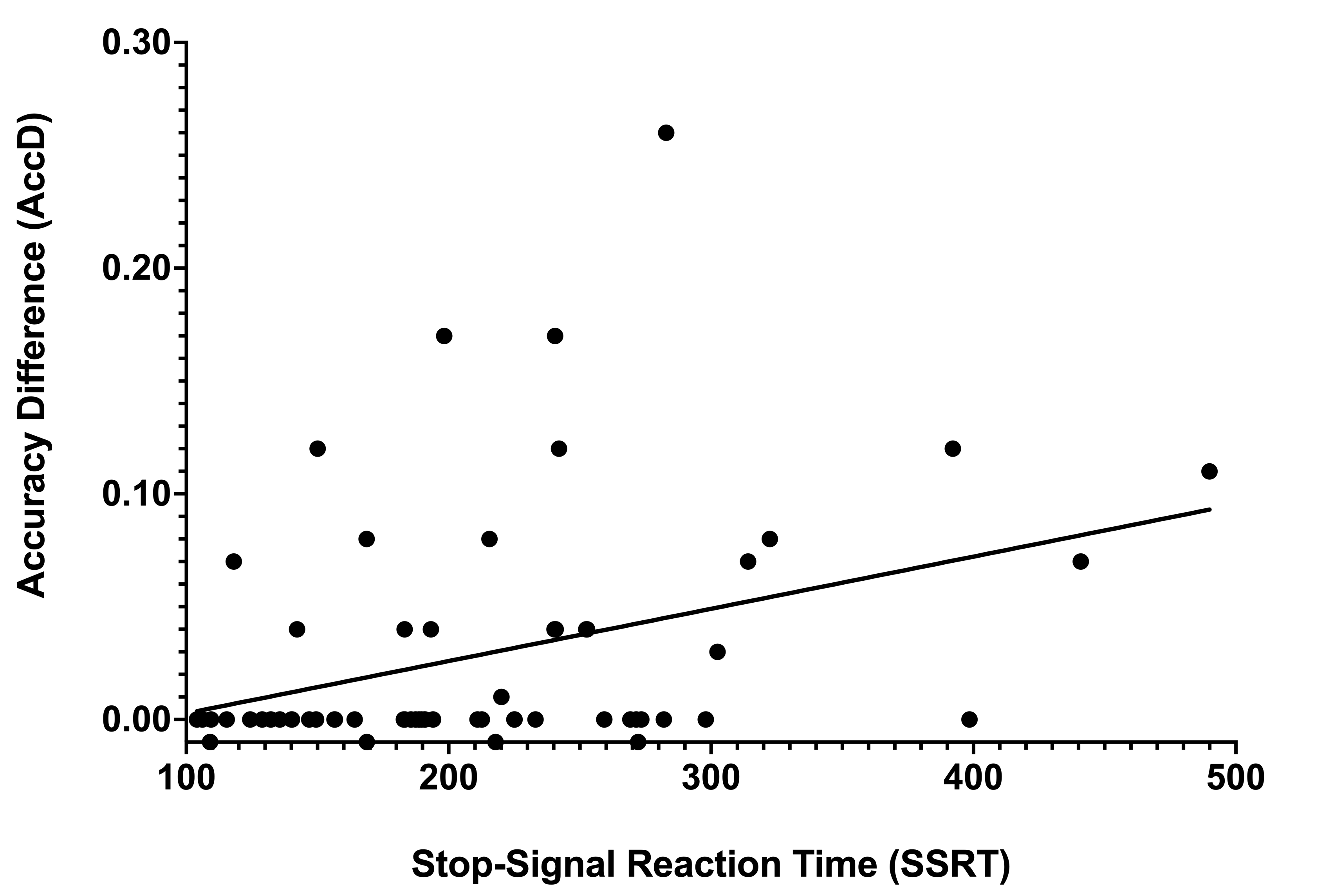

Supplement: S4 Fig — (DOCX) [file pone.0260695.s014.docx]
