# Supplementary material for: The early childhood inhibitory touchscreen task: A new measure of response inhibition in toddlerhood and across the lifespan
Source: PLoS One. 2021 Dec 2;16(12):e0260695. doi: 10.1371/journal.pone.0260695 (PMC8638877; doi:10.1371/journal.pone.0260695)
Supplement: S1 Table — (DOCX) [file pone.0260695.s015.docx]

**S1 Table.** Demographic information for participants in Studies 1, 2 and 3 (large version)

| Sample | Birkbeck Toddlers | Oxford Toddlers | VT Toddlers (longitudinal) | Children | Young Adults | Older Adults |
| --- | --- | --- | --- | --- | --- | --- |
| *N* | 47(44)* | 39(38)** | 38 | 27 | 17 | 20 |
|  | *Mean or %* | *Mean or %* | *Mean or %* | *Mean or %* | *Mean or %* | *Mean or %* |
| Age (months/years) | 29.68 mths | 24.03 mths | 18.27, 21.16 & 24.20 mths | 7.93 yrs | 22.88 yrs | 69.65 yrs |
| Sex |  |  |  |  |  |  |
| % Female | 56.82% (25/44) | 42.11% (16/38) | 44.74% (17/38) | 66.67% (18/27) | 82.35% (14/17) | 55.00% (11/20) |
| Ethnicity |  |  |  |  |  |  |
| White (British, Irish, American or Other) | 79.55% (35/44) | 76.32% (29/38) | 89.47% (34/38) | -- | -- | -- |
| Asian | 0.00% (0/44) | 0.00% (0/38) | 2.63% (1/38) |  |  |  |
| Afro-Caribbean | 2.27% (1/44) | 0.00% (0/38) | 0.00% (0/38) | -- | -- | -- |
| Other Black Background | 0.00% (0/44) | 0.00% (0/38) | 0.00% (0/38) | -- | -- | -- |
| Mixed - White and Asian | 9.09% (4/44) | 5.26% (2/38) | 0.00% (0/38) | -- | -- | -- |
| Mixed - White and Black | 2.27% (1/44) | 2.63% (1/38) | 7.89% (3/38) | -- | -- | -- |
| Other Mixed Background | 6.82% (3/44) | 2.63% (1/38) | 0.00% (0/38) | -- | -- | -- |
| Not provided | 0.00% (0/44) | 13.16% (5/38) | 0.00% (0/38) | -- | -- | -- |
| Highest level of education (adult participants) |  |  |  |  |  |  |
| GCSEs | -- | -- | -- | -- | 0.00% (0/17) | 70.00% (14/20) |
| A-levels | -- | -- | -- | -- | 82.35% (14/17) | 0.00% (0/20) |
| Degree / Higher National Diploma | -- | -- | -- | -- | 17.65% (3/17) | 15.00% (3/20) |
| Postgraduate degree / Doctorate | -- | -- | -- | -- | 0.00% (0/17) | 0.00% (0/20) |
| Not provided | -- | -- | -- | -- | 0.00% (0/17) | 15.00% (3/20) |
| Total years in education | -- | -- | -- | -- | 16 (17/17) | 12.78 (18/20) |
| Household Income |  |  |  |  |  |  |
| Under £15,000 | 2.27% (1/44) | 5.26% (2/38) | -- | 3.70% (1/27) | 29.41% (5/17) | 20.00% (4/20) |
| £15,000 - £30,000 | 0.00% (0/44) | 7.89% (3/38) | -- | 25.93% (7/27) | 35.29% (6/17) | 15.00% (3/20) |
| £30,000 - £45,000 | 0.00% (0/44) | 7.89% (3/38) | -- | 14.81% (4/27) | 23.53% (4/17) | 50.00% (10/20) |
| £45,000 - £60,000 | 9.09% (4/44) | 7.89% (3/38) | -- | 7.40% (2/27) | 5.88% (1/17) | 10.00% (2/20) |
| Over £60,000 | 68.18% (30/44) | 50.00% (19/38) | -- | 0.00% (0/27) | 0.00% (0/17) | 0.00% (0/20) |
| Not provided | 20.45% (9/44) | 21.05% (8/38) | -- | 48.15% (13/27) | 5.88% (1/17) | 5.00% (1/20) |
| Maternal Characteristics |  |  |  |  |  |  |
| Age (years)*** | 37.00 (36/44) | 36.03 (34/38) | 31.21 (38/38) | 35.00 (20/27) | -- | -- |
| Total years in education | 17.86 (44/44) | 17.27 (30/38) | 17.29 (38/38) | 14.42 (19/27) | -- | -- |

*Note.* Numbers in brackets indicate the frequency of a category/characteristic out of the total participant sample. *Three participants were excluded prior to analysis (2 boys and 1 girl), one due to experimenter error and two due to no video being recorded during the session; these participants are not included in the remainder of the table. **One participant (a girl) refused to engage with the Early Childhood Inhibitory Touchscreen task and is not included in the remainder of the table. ***For the Oxford and Birkbeck toddlers, maternal age was reported at the time of the test session (24-month and 30-month session, respectively); for the Virginia Tech (VT) toddlers, maternal age was reported at the child's birth. -- Indicates that this type of demographic data was not collected in this sample.
