# Supplementary material for: The early childhood inhibitory touchscreen task: A new measure of response inhibition in toddlerhood and across the lifespan
Source: PLoS One. 2021 Dec 2;16(12):e0260695. doi: 10.1371/journal.pone.0260695 (PMC8638877; doi:10.1371/journal.pone.0260695)
Supplement: S2 Table — (DOCX) [file pone.0260695.s016.docx]

**S2 Table.** Correlations between accuracy difference (AccD) scores at 18, 21 and 24 months of age in the longitudinal sample in Study 2 with one participant excluded due to having a reaction time difference (RTD) score more than 3 standard deviations above the group mean at 18 months (95% confidence intervals using bootstrapping with 1000 samples in brackets). ** *p* < .01.

|  | AccD 21 months | AccD 24 months |
| --- | --- | --- |
| AccD 18 months | *r* = .642** (.235; .814)  *p* = .001  *n* = 22 | *r* = .402 (-.387; .705)  *p* = .099  *n* = 18 |
| AccD 21 months |  | *r* = .658** (.024; .883)  *p* = .001  *n* = 23 |
