# Supplementary material for: The early childhood inhibitory touchscreen task: A new measure of response inhibition in toddlerhood and across the lifespan
Source: PLoS One. 2021 Dec 2;16(12):e0260695. doi: 10.1371/journal.pone.0260695 (PMC8638877; doi:10.1371/journal.pone.0260695)
Supplement: S3 Table — (DOCX) [file pone.0260695.s017.docx]

**S3 Table.** Correlations between reaction time difference (RTD) scores at 18, 21 and 24 months of age in the longitudinal sample in Study 2 with one participant excluded due to having a RTD score more than 3 standard deviations above the group mean at 18 months (95% confidence intervals using bootstrapping with 1000 samples in brackets).

|  | RTD 21 months | RTD 24 months |
| --- | --- | --- |
| RTD 18 months | *r* = .242 (-.187; .650)  *p* = .333  *n* = 18 | *r* = -.381 (-.794; .306)  *p* = .161  *n* = 15 |
| RTD 21 months |  | *r* = .099 (-.209; .655)  *p* = .669  *n* = 21 |
