# Supplementary material for: The early childhood inhibitory touchscreen task: A new measure of response inhibition in toddlerhood and across the lifespan
Source: PLoS One. 2021 Dec 2;16(12):e0260695. doi: 10.1371/journal.pone.0260695 (PMC8638877; doi:10.1371/journal.pone.0260695)
Supplement: S4 Table — (DOCX) [file pone.0260695.s018.docx]

**
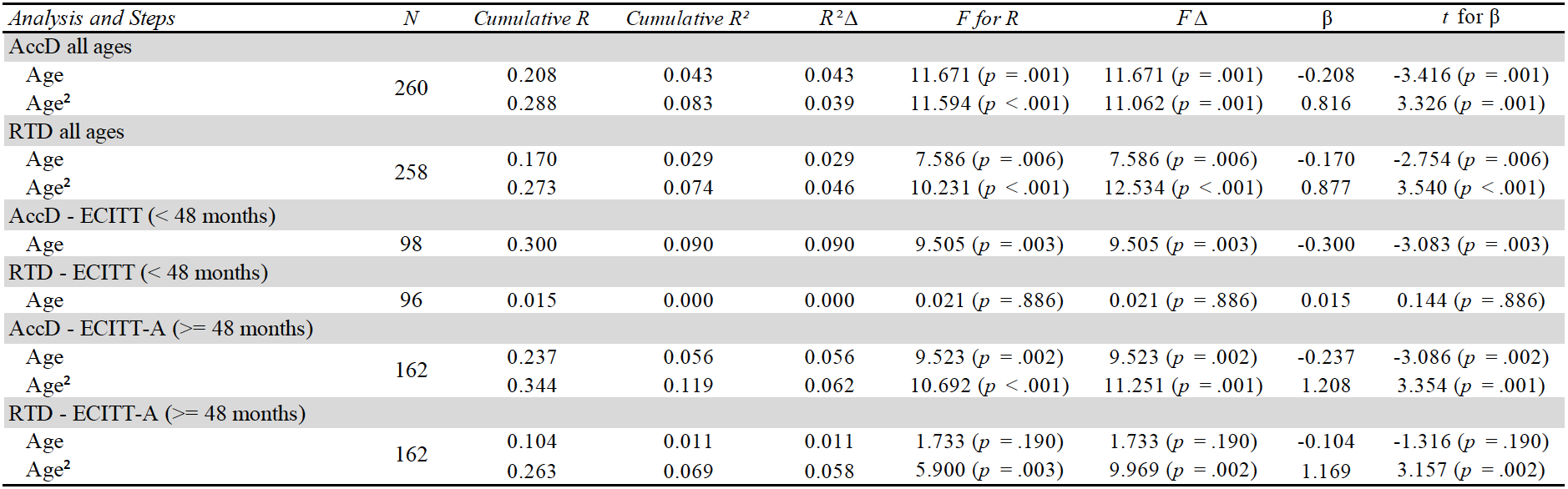
 S4 Table.** Hierarchical regression analyses of age as a predictor of Early Childhood Inhibitory Touchscreen Task (ECITT) and Early Childhood Inhibitory Touchscreen Task – Adult version (ECITT-A) accuracy difference (AccD) and reaction time difference (RTD) scores in participants with age in months available across Studies 1, 3, 4 and the Pilot Study. All participants under 4 years were administered the ECITT, and all participants aged 4 years and over were administered the ECITT-A. Thirty-three participants with information on age only in years were excluded. In addition to this, 7 toddlers were excluded from the sample because they had < 60% accuracy on prepotent trials.
